# Supplementary material for: Assessing self-selection biases in Facebook-recruited online surveys: Evidence from the COVID-19 Health Behavior Survey
Source: PLoS One. 2025 Jul 8;20(7):e0326884. doi: 10.1371/journal.pone.0326884 (PMC12237053; doi:10.1371/journal.pone.0326884)
Supplement: S1 File — (PDF) [file pone.0326884.s001.pdf]

Assessing self-selection biases in online surveys: Evidence from the COVID-19 Health Behavior Survey.

Supporting Information

List of Figures

S1 Overview of the study design used in this research 5
S2 Flowchart of the data preparation process of the COVID-19 Health Behavior Survey 6
S3 Respondents' educational status by advertisement image and country. 13
S4 Step-wise predicted probability of threat perception of COVID-19 to the family by advertisement image (on the y-axis) and Belgium, France, Germany and Italy. 14
S5 Step-wise predicted probability of threat perception of COVID-19 to the family by advertisement image (on the y-axis) and the Netherlands, Spain, the United Kingdom and the United States. 15
S6 Step-wise predicted probability of threat perception of COVID-19 to the oneself by advertisement image (on the y-axis) and Belgium, France, Germany and Italy. 16
S7 Step-wise predicted probability of threat perception of COVID-19 to the oneself by advertisement image (on the y-axis) and the Netherlands, Spain, the United Kingdom and the United States. 17
S8 Step-wise predicted probability of wearing a face mask by advertisement image (on the y-axis) and Belgium, France, Germany and Italy. 18
S9 Step-wise predicted probability of wearing a face mask by advertisement image (on the y-axis) and the Netherlands, Spain, the United Kingdom and the United States. 19
S10 Step-wise predicted probability of increased hand washing by advertisement image (on the y-axis) and Belgium, France, Germany and Italy. 20

S11 Step-wise predicted probability of increased hand washing by advertisement image (on the y-axis) and the Netherlands, Spain, the United Kingdom and the United States. 21

List of Tables

S1 Grouping of the education survey options in questionnaire in the United States to match the education categories in the other countries. 3
S2 Question wordings concerning preventive behaviors. 4
S3 Facebook reach and link clicks by advertisement image and country. . 7
S4 Descriptive statistics by country and advertising image, showing sex ratios and age distribution (median age and IQR in brackets). 9
S5 Facebook impressions by advertisement image and gender and age for Belgium, France, and Germany. 10
S6 Facebook impressions by advertisement image and gender and age for Italy, the Netherlands and Spain. 11
S7 Facebook impressions by advertisement image and gender and age for the United Kingdom and the United States. 12

Survey option	Education category
1 or more years of college credit, no degree	Secondary school or lower
12th grade - NO DIPLOMA	Secondary school or lower
Grade one through 11	Secondary school or lower
GED or alternative credential	Secondary school or lower
Kindergarten	Secondary school or lower
No formal education	Secondary school or lower
No schooling completed	Secondary school or lower
Primary school	Secondary school or lower
Regular high school diploma	Secondary school or lower
Secondary school	Secondary school or lower
Some college credit, but less than 1 year of college credit	Secondary school or lower
Associates degree (e.g., AA, AS)	University level
Bachelors degree (e.g., BA, BS)	University level
Masters degree (e.g., MA, MS, MEng, MEd, MSW, MBA)	University level
University-level education (e.g., bachelor's degree, master's degree)	University level
Doctorate degree (e.g., PhD, EdD)	Postgraduate Degree
Postgraduate degree (e.g., PhD, medical doctorate)	Postgraduate Degree
Professional degree beyond a bachelors degree (e.g., MD, DDS, DVM, LLB, JD)	Postgraduate Degree

Table S1: Grouping of the education survey options in questionnaire in the United States to match the education categories in the other countries.

Question wordings from March 12, 2020 to May 7, 2020	Question wordings from May 7, 2020 to August 12, 2020
Which of the following actions, if any, have you already taken to protect yourself from the coronavirus?	The list below shows again the same measures. This time, we would like to know which of these measures you have already taken to keep you and others from getting infected with the new coronavirus.
Action wordings from March 12, 2020 to May 7, 2020	Response wordings from May 7, 2020 to August 12, 2020
Worn a face mask	Wearing a protective face mask in public (especially when visiting busy, closed spaces, such as grocery stores, shopping centers, or when using public transport, etc.)
Washed hands more often	Washing your hands often with soap and water for at least 20 seconds
Response options from March 12, 2020 to May 7, 2020	Response wordings from May 7, 2020 to August 12, 2020
Yes / No	Yes/ No/ Don't know/ Prefer not to answer

Question wording for the outcome of wearing a face mask and increasing hand washing, in the "COVID-19 Health Behavior Survey", before and after implementing some changes to the questionnaire on May 7, 2020.

Table S2: Question wordings concerning preventive behaviors.

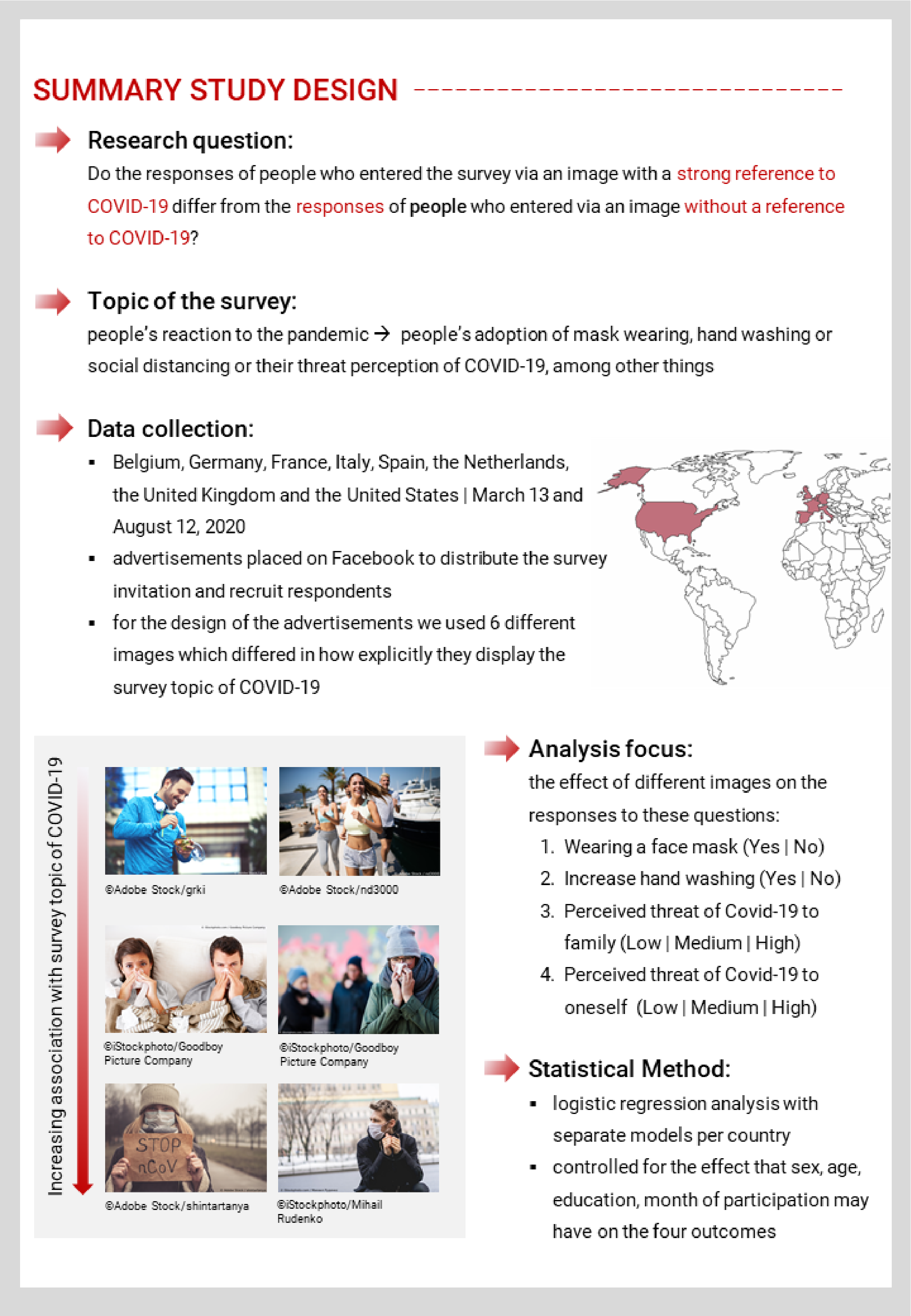

Figure S1: Overview of the study design used in this research

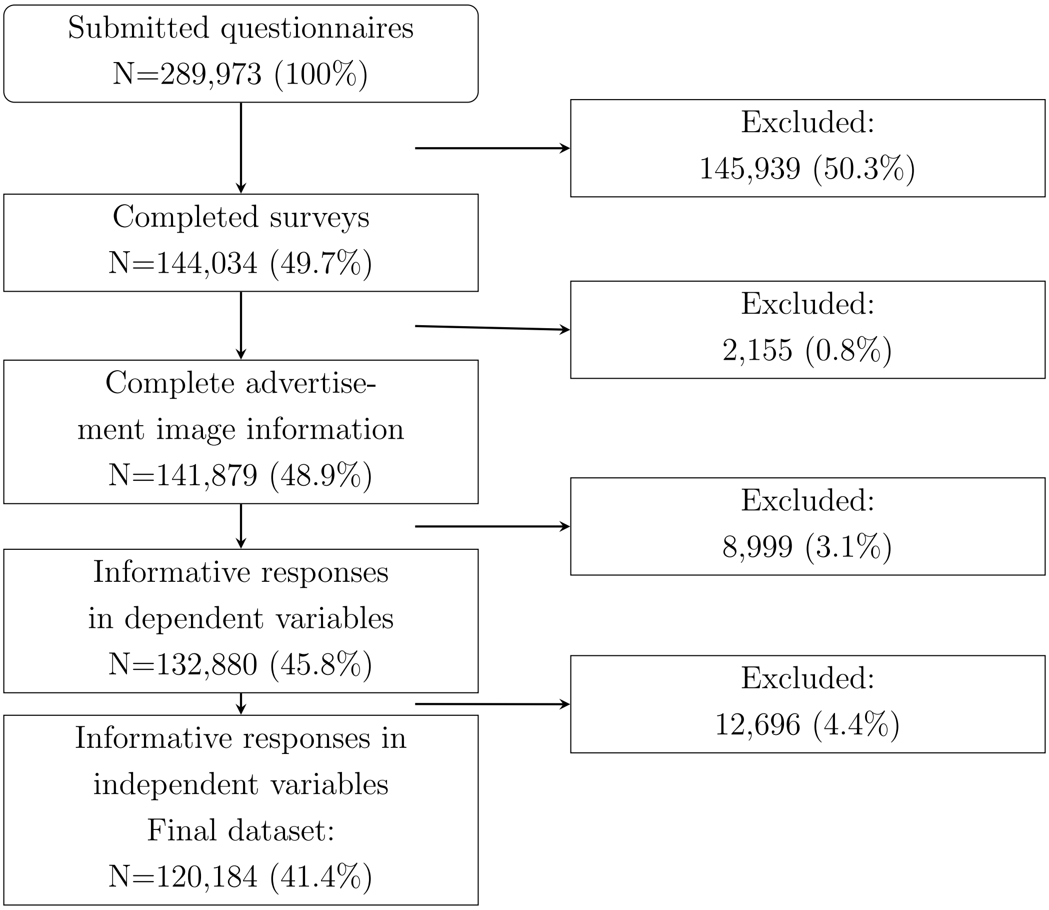

Figure S2: Flowchart of the data preparation process of the COVID-19 Health Behavior Survey

Country	1 - Male athlete	2 - Group of athletes	3 - Woman blowing nose	4 - Couple blowing noses	5 - Woman wearing mask	6 - Man wearing mask	Total
**Reach**							
BE	63345 (4.6%)	198689 (14.6%)	224747 (16.5%)	100394 (7.4%)	520814 (38.2%)	256952 (18.8%)	1364941 (100.0%)
FR	43968 (2.3%)	331656 (17.1%)	295546 (15.2%)	115462 (5.9%)	919925 (47.4%)	234058 (12.1%)	1940615 (100.0%)
DE	62662 (2.4%)	196876 (7.6%)	531648 (20.6%)	101132 (3.9%)	1283363 (49.8%)	400641 (15.6%)	2576322 (100.0%)
IT	264990 (4.7%)	923367 (16.4%)	878650 (15.6%)	336139 (6.0%)	2620848 (46.5%)	610514 (10.8%)	5634508 (100.0%)
NL	77492 (4.5%)	226966 (13.1%)	418282 (24.2%)	148299 (8.6%)	601557 (34.8%)	255274 (14.8%)	1727870 (100.0%)
ES	834902 (8.1%)	2210993 (21.5%)	1870019 (18.2%)	972593 (9.4%)	2736582 (26.6%)	1673384 (16.2%)	10298473 (100.0%)
UK	110195 (4.4%)	263026 (10.5%)	368143 (14.6%)	186986 (7.4%)	1282140 (51.0%)	304748 (12.1%)	2515238 (100.0%)
US	250864 (6.1%)	974485 (23.9%)	305529 (7.5%)	277172 (6.8%)	1855355 (45.4%)	419466 (10.3%)	4082871 (100.0%)
Total	1708418 (5.7%)	5326058 (17.7%)	4892564 (16.2%)	2238177 (7.4%)	11820584 (39.2%)	4155037 (13.8%)	30140838 (100.0%)
**Clicks**							
BE	1258 (1.3%)	5314 (5.4%)	19132 (19.3%)	3524 (3.5%)	41657 (42.0%)	28384 (28.6%)	99269 (100.0%)
FR	472 (0.4%)	10478 (8.7%)	17954 (14.9%)	2314 (1.9%)	72464 (60.2%)	16703 (13.9%)	120385 (100.0%)
DE	1358 (0.5%)	7346 (3.0%)	55478 (22.4%)	4743 (1.9%)	135521 (54.8%)	42859 (17.3%)	247305 (100.0%)
IT	5798 (2.0%)	39366 (13.7%)	51519 (18.0%)	8084 (2.8%)	152023 (53.1%)	29653 (10.4%)	286443 (100.0%)
NL	1785 (1.1%)	7022 (4.3%)	59985 (36.4%)	7024 (4.3%)	60014 (36.4%)	29076 (17.6%)	164906 (100.0%)
ES	39249 (7.6%)	113005 (21.9%)	102595 (19.9%)	25504 (4.9%)	145235 (28.1%)	91236 (17.7%)	516824 (100.0%)
UK	2203 (1.5%)	6594 (4.4%)	21493 (14.5%)	5283 (3.6%)	93467 (63.0%)	19241 (13.0%)	148281 (100.0%)
US	3863 (1.8%)	32434 (15.0%)	11094 (5.1%)	5533 (2.6%)	138274 (63.9%)	25080 (11.6%)	216278 (100.0%)
**Total**	55986 (3.1%)	221559 (12.3%)	339250 (18.9%)	62009 (3.4%)	838655 (46.6%)	282232 (15.7%)	1799691 (100.0%)

The Facebook reach is the number of unique Facebook users who saw the advertisement. The link clicks by advertisement are numbers of unique Facebook users who clicked on the advertisement.

Table S3: Facebook reach and link clicks by advertisement image and country.

1 Covariates by advertisement image

The majority of the respondents were women, however athlete images (images 1 and 2) recruited a higher share of men. Specifically, the "group of athletes" image (image 2) recruited more men in all countries, whereas the "male athlete" image (image 1) recruited more men than women in Belgium, France, the Netherlands, and the United Kingdom (Table S4).Certain images tended to attract younger participants. In all countries, the "woman wearing mask" images (image 5) recruited participants below the median age of the respective country. The "male athlete" (image 1) attracted younger participants in all countries, expect for Germany and Spain. Similarly, the "group of athletes" (image 2) attracted younger participants in Belgium and the Netherlands, and the "couple blowing nose" image (image 4) in France and the United States (Table S4). In general, the age and gender composition by ad image in each country aligns with the number of impressions (i.e., the number of times the ad was displayed) recorded for the ad sets targeting by gender and age (Tables S5 to S7). Taking the United States as an example, only 15% of the respondents recruited through the "group of athletes" image (image 2) reported being female in the survey, and the median age for this image is higher than the overall median age for the country in the survey–63 compared to 58. Looking at the number of impressions, we see that the "group of athletes" image (image 2) received a significantly higher share of impressions in the two older age groups (i.e., 45-65 and 65+) with about 54% compared to just 6% for the ad sets targeted to women in those same age groups. We observe that some images recruited higher shares of respondents with a university level education in comparison to the country average. The "male athlete" image (image 1) recruited a higher percentage of university-educated participants, ranging between six and 12 percentage points higher than the country total, in Germany, the Netherlands, the United States, and France. The "group of athletes" image (image 2) showed a similar pattern in Belgium, Germany and the Netherlands. The "woman wearing mask" image (image 5) correlated with higher university education in Belgium, France, Spain, the United Kingdom, and the Netherlands although with smaller differences with maximum eight percentages point above the total country (Figure S3).

	**IMG 1** Male athlete	**IMG 2** Group of athletes	**IMG 3** Woman blowing nose	**IMG 4** Couple blowing noses	**IMG 5** Woman wearing mask	**IMG 6** Man wearing mask	Country total
	**Female-to-male ratio in sample composition (%)**						
BE	45%	39%	66%	66%	63%	72%	66%
FR	17%	24%	69%	86%	69%	74%	70%
DE	58%	25%	73%	62%	60%	59%	60%
IT	66%	42%	73%	71%	65%	71%	67%
NL	40%	32%	71%	67%	58%	68%	62%
ES	69%	45%	72%	66%	68%	68%	68%
UK	46%	23%	72%	67%	65%	61%	65%
US	72%	15%	70%	67%	64%	63%	64%
	**Respondents' median age (IQR)**						
BE	43 (24-66)	44 (22-66)	60 (50-66)	53 (23-67)	43 (29-59)	60 (46-66)	51 (34-63)
FR	36 (24-51)	58 (34-66)	58 (44-64)	29 (22-54)	44 (29-69)	61 (47-69)	48 (30-62)
DE	56 (37-65)	52 (24-69)	58 (49-65)	55 (34-66)	40 (28-55)	56 (42-65)	42 (30-58)
IT	35 (24-48)	41 (24-64)	55 (40-63)	42 (28-61)	40 (28-55)	59 (43-67)	42 (29-58)
NL	56 (22-66)	52 (23-68)	62 (54-67)	58 (36-67)	51 (35-62)	62 (55-69)	57 (41-65)
ES	54 (38-60)	53 (36-64)	56 (45-63)	53 (38-62)	45 (36-58)	59 (48-65)	52 (39-61)
UK	40 (23-62)	61 (45-70)	62 (54-68)	60 (42-69)	56 (41-64)	64 (57-70)	58 (43-66)
US	41 (30-59)	63 (52-70)	63 (51-70)	54 (34-67)	57 (39-66)	64 (55-71)	58 (40-67)

Table S4: Descriptive statistics by country and advertising image, showing sex ratios and age distribution (median age and IQR in brackets).

Country	Age	1 - Male athlete	2 - Group of athletes	3 - Woman blowing nose	4 - Couple blowing noses	5 - Woman wearing mask	6 - Man wearing mask	Total
**Men**								
BE	18-24	68,999 (10.4%)	154,363 (23.3%)	79,964 (12.0%)	56,104 (8.5%)	216,820 (32.7%)	87,542 (13.2%)	663,792 (100.0%)
BE	25-44	22,508 (4.0%)	88,672 (15.8%)	95,550 (17.1%)	32,188 (5.8%)	247,981 (44.3%)	72,842 (13.0%)	559,741 (100.0%)
BE	45-64	3,075 (0.7%)	91,103 (19.5%)	88,690 (19.0%)	16,056 (3.4%)	188,797 (40.5%)	78,752 (16.9%)	466,473 (100.0%)
BE	65+	4,799 (1.2%)	117,148 (28.9%)	60,612 (14.9%)	16,996 (4.2%)	122,998 (30.3%)	83,310 (20.5%)	405,863 (100.0%)
**Women**								
BE	18-24	43,475 (8.0%)	67,519 (12.4%)	65,272 (12.0%)	60,312 (11.1%)	231,343 (42.4%)	77,541 (14.2%)	545,462 (100.0%)
BE	25-44	6,824 (1.6%)	14,770 (3.5%)	61,100 (14.4%)	14,882 (3.5%)	222,658 (52.5%)	104,234 (24.6%)	424,468 (100.0%)
BE	45-64	1,026 (0.3%)	3,873 (1.1%)	94,579 (26.1%)	12,220 (3.4%)	124,857 (34.4%)	126,459 (34.8%)	363,014 (100.0%)
BE	65+	2,275 (0.7%)	6,430 (2.0%)	78,474 (24.9%)	15,421 (4.9%)	78,620 (25.0%)	133,762 (42.5%)	314,982 (100.0%)
Total	-	152,981 (4.1%)	543,878 (14.5%)	624,241 (16.7%)	224,179 (6.0%)	1,434,074 (38.3%)	764,442 (20.4%)	3,743,795 (100.0%)
**Men**								
FR	18-24	70,349 (10.9%)	144,173 (22.4%)	85,786 (13.3%)	45,633 (7.1%)	241,129 (37.4%)	56,814 (8.8%)	643,884 (100.0%)
FR	25-44	4,196 (0.8%)	113,915 (21.0%)	112,688 (20.7%)	28,248 (5.2%)	248,111 (45.7%)	35,979 (6.6%)	543,137 (100.0%)
FR	45-64	266 (0.1%)	156,701 (30.5%)	93,342 (18.2%)	10,685 (2.1%)	217,426 (42.4%)	34,641 (6.8%)	513,061 (100.0%)
FR	65+	213 (0.0%)	173,981 (39.7%)	41,874 (9.6%)	10,687 (2.4%)	157,379 (35.9%)	54,087 (12.3%)	438,221 (100.0%)
**Women**								
FR	18-24	3,547 (0.8%)	39,569 (9.2%)	52,615 (12.2%)	45,138 (10.5%)	225,208 (52.2%)	65,518 (15.2%)	431,595 (100.0%)
FR	25-44	36 (0.0%)	10,935 (2.6%)	70,080 (16.8%)	23,862 (5.7%)	271,142 (65.1%)	40,707 (9.8%)	416,762 (100.0%)
FR	45-64	185 (0.0%)	6,029 (1.4%)	92,792 (22.3%)	7,376 (1.8%)	241,477 (58.0%)	68,581 (16.5%)	416,440 (100.0%)
FR	65+	422 (0.1%)	12,702 (3.6%)	59,968 (16.8%)	8,151 (2.3%)	180,001 (50.4%)	95,577 (26.8%)	356,821 (100.0%)
Total	-	79,214 (2.1%)	658,005 (17.5%)	609,145 (16.2%)	179,780 (4.8%)	1,781,873 (47.4%)	451,904 (12.0%)	3,759,921 (100.0%)
**Men**								
DE	18-24	109,359 (10.1%)	89,534 (8.2%)	195,837 (18.0%)	59,416 (5.5%)	492,483 (45.3%)	141,036 (13.0%)	1,087,665 (100.0%)
DE	25-44	4,576 (0.5%)	70,361 (8.0%)	177,002 (20.1%)	16,417 (1.9%)	514,430 (58.6%)	95,669 (10.9%)	878,455 (100.0%)
DE	45-64	2,013 (0.2%)	76,049 (9.1%)	223,043 (26.8%)	16,793 (2.0%)	389,794 (46.9%)	123,805 (14.9%)	831,497 (100.0%)
DE	65+	2,675 (0.4%)	188,356 (26.2%)	114,024 (15.8%)	23,697 (3.3%)	252,763 (35.1%)	138,141 (19.2%)	719,656 (100.0%)
**Women**								
DE	18-24	16,028 (2.2%)	35,008 (4.7%)	83,783 (11.3%)	40,961 (5.5%)	461,084 (62.4%)	101,671 (13.8%)	738,535 (100.0%)
DE	25-44	1,645 (0.2%)	5,301 (0.8%)	134,186 (19.4%)	9,132 (1.3%)	424,610 (61.5%)	115,790 (16.8%)	690,664 (100.0%)
DE	45-64	2,427 (0.3%)	3,680 (0.5%)	332,148 (43.6%)	18,326 (2.4%)	252,818 (33.2%)	151,605 (19.9%)	761,004 (100.0%)
DE	65+	3,688 (0.6%)	7,541 (1.2%)	209,348 (33.1%)	24,119 (3.8%)	244,564 (38.6%)	143,974 (22.7%)	633,234 (100.0%)
Total	-	142,411 (2.2%)	475,830 (7.5%)	1,469,371 (23.2%)	208,861 (3.3%)	3,032,546 (47.8%)	1,011,691 (16.0%)	6,340,710 (100.0%)

The Facebook impression is number of times the ad was displayed.

Table S5: Facebook impressions by advertisement image and gender and age for Belgium, France, and Germany.

Country	Age	1 - Male athlete	2 - Group of athletes	3 - Woman blowing nose	4 - Couple blowing noses	5 - Woman wearing mask	6 - Man wearing mask	Total
**Men**								
IT	18-24	429,556 (21.4%)	446,263 (22.3%)	197,759 (9.9%)	141,521 (7.1%)	656,367 (32.7%)	133,311 (6.6%)	2,004,777 (100.0%)
IT	25-44	132,174 (7.0%)	421,065 (22.2%)	225,110 (11.9%)	139,004 (7.3%)	861,345 (45.5%)	115,891 (6.1%)	1,894,589 (100.0%)
IT	45-64	6,118 (0.4%)	485,353 (35.1%)	183,206 (13.2%)	25,239 (1.8%)	596,648 (43.1%)	86,820 (6.3%)	1,383,384 (100.0%)
IT	65+	2,942 (0.3%)	513,151 (47.5%)	82,165 (7.6%)	20,526 (1.9%)	350,512 (32.5%)	110,859 (10.3%)	1,080,155 (100.0%)
**Women**								
IT	18-24	171,142 (10.7%)	208,285 (13.0%)	236,950 (14.8%)	147,940 (9.3%)	655,445 (41.0%)	177,923 (11.1%)	1,597,685 (100.0%)
IT	25-44	18,062 (1.3%)	78,899 (5.5%)	293,162 (20.6%)	79,018 (5.6%)	839,561 (59.0%)	113,551 (8.0%)	1,422,253 (100.0%)
IT	45-64	3,077 (0.2%)	41,589 (3.2%)	422,592 (32.1%)	38,620 (2.9%)	647,593 (49.2%)	163,901 (12.4%)	1,317,372 (100.0%)
IT	65+	6,142 (0.6%)	38,118 (3.7%)	248,473 (24.3%)	34,862 (3.4%)	484,635 (47.4%)	211,209 (20.6%)	1,023,439 (100.0%)
Total	-	769,213 (6.6%)	2,232,723 (19.0%)	1,889,417 (16.1%)	626,730 (5.3%)	5,092,106 (43.4%)	1,113,465 (9.5%)	11,723,654 (100.0%)
**Men**								
NL	18-24	147,495 (16.7%)	162,770 (18.4%)	142,676 (16.1%)	97,517 (11.0%)	255,262 (28.9%)	78,131 (8.8%)	883,851 (100.0%)
NL	25-44	32,915 (4.1%)	125,884 (15.6%)	228,166 (28.3%)	53,609 (6.7%)	309,456 (38.4%)	55,678 (6.9%)	805,708 (100.0%)
NL	45-64	3,352 (0.5%)	101,151 (16.3%)	196,145 (31.7%)	23,515 (3.8%)	218,404 (35.3%)	76,122 (12.3%)	618,689 (100.0%)
NL	65+	2,830 (0.6%)	123,411 (24.5%)	122,128 (24.2%)	25,587 (5.1%)	133,379 (26.5%)	96,434 (19.1%)	503,769 (100.0%)
**Women**								
NL	18-24	52,577 (7.6%)	71,672 (10.3%)	113,360 (16.3%)	100,366 (14.4%)	247,374 (35.6%)	109,229 (15.7%)	694,578 (100.0%)
NL	25-44	7,457 (1.3%)	25,055 (4.2%)	190,677 (32.3%)	38,867 (6.6%)	257,918 (43.7%)	69,974 (11.9%)	589,948 (100.0%)
NL	45-64	428 (0.1%)	1,145 (0.2%)	218,232 (44.7%)	22,085 (4.5%)	132,140 (27.1%)	114,079 (23.4%)	488,109 (100.0%)
NL	65+	960 (0.2%)	1,348 (0.3%)	185,364 (44.2%)	23,970 (5.7%)	82,742 (19.7%)	124,948 (29.8%)	419,332 (100.0%)
Total	-	248,014 (5.0%)	612,436 (12.2%)	1,396,748 (27.9%)	385,516 (7.7%)	1,636,675 (32.7%)	724,595 (14.5%)	5,003,984 (100.0%)
**Men**								
ES	18-24	1,527,181 (31.4%)	1,197,106 (24.6%)	483,987 (9.9%)	407,790 (8.4%)	722,885 (14.8%)	531,305 (10.9%)	4,870,254 (100.0%)
ES	25-44	900,612 (17.6%)	1,763,559 (34.4%)	676,948 (13.2%)	277,180 (5.4%)	1,067,293 (20.8%)	438,554 (8.6%)	5,124,146 (100.0%)
ES	45-64	115,922 (2.5%)	2,081,361 (44.9%)	559,466 (12.1%)	186,229 (4.0%)	1,168,184 (25.2%)	519,665 (11.2%)	4,630,827 (100.0%)
ES	65+	90,433 (2.7%)	1,501,592 (44.7%)	348,427 (10.4%)	196,068 (5.8%)	719,396 (21.4%)	505,695 (15.0%)	3,361,611 (100.0%)
**Women**								
ES	18-24	1,090,386 (23.6%)	882,821 (19.1%)	632,131 (13.7%)	517,065 (11.2%)	872,810 (18.9%)	620,931 (13.5%)	4,616,144 (100.0%)
ES	25-44	410,712 (9.5%)	679,759 (15.7%)	933,321 (21.5%)	336,294 (7.8%)	1,411,196 (32.5%)	564,197 (13.0%)	4,335,479 (100.0%)
ES	45-64	79,994 (1.9%)	525,059 (12.3%)	1,227,265 (28.7%)	227,547 (5.3%)	1,286,001 (30.1%)	930,435 (21.8%)	4,276,301 (100.0%)
ES	65+	79,410 (2.3%)	417,178 (12.0%)	707,247 (20.3%)	302,231 (8.7%)	904,325 (26.0%)	1,067,615 (30.7%)	3,478,006 (100.0%)
Total	-	4,294,650 (12.4%)	9,048,435 (26.1%)	5,568,792 (16.1%)	2,450,404 (7.1%)	8,152,090 (23.5%)	5,178,397 (14.9%)	34,692,768 (100.0%)

The Facebook impression is number of times the ad was displayed.

Table S6: Facebook impressions by advertisement image and gender and age for Italy, the Netherlands and Spain.

Country	Age	1 - Male athlete	2 - Group of athletes	3 - Woman blowing nose	4 - Couple blowing noses	5 - Woman wearing mask	6 - Man wearing mask	Total
**Men**								
UK	18-24	133,156 (15.6%)	149,960 (17.5%)	87,165 (10.2%)	91,140 (10.7%)	303,976 (35.5%)	90,184 (10.5%)	855,581 (100.0%)
UK	25-44	55,000 (6.7%)	128,038 (15.7%)	120,639 (14.8%)	52,620 (6.4%)	385,095 (47.1%)	75,485 (9.2%)	816,877 (100.0%)
UK	45-64	4,725 (0.8%)	108,678 (17.3%)	76,992 (12.3%)	18,847 (3.0%)	351,095 (55.9%)	67,324 (10.7%)	627,661 (100.0%)
UK	65+	1,775 (0.4%)	98,303 (20.0%)	60,284 (12.3%)	21,762 (4.4%)	235,884 (48.1%)	72,735 (14.8%)	490,743 (100.0%)
**Women**								
UK	18-24	75,126 (11.1%)	65,538 (9.7%)	96,642 (14.3%)	86,428 (12.8%)	261,995 (38.7%)	90,916 (13.4%)	676,645 (100.0%)
UK	25-44	11,899 (2.0%)	17,246 (2.8%)	127,118 (20.9%)	46,609 (7.7%)	346,552 (56.9%)	59,128 (9.7%)	608,552 (100.0%)
UK	45-64	247 (0.1%)	2,856 (0.6%)	114,979 (24.1%)	18,218 (3.8%)	272,573 (57.2%)	67,335 (14.1%)	476,208 (100.0%)
UK	65+	679 (0.2%)	1,947 (0.5%)	89,590 (24.0%)	21,879 (5.9%)	175,278 (46.9%)	84,126 (22.5%)	373,499 (100.0%)
Total	-	282,607 (5.7%)	572,566 (11.6%)	773,409 (15.7%)	357,503 (7.3%)	2,332,448 (47.4%)	607,233 (12.3%)	4,925,766 (100.0%)
**Men**								
US	18-24	200,447 (11.6%)	502,171 (29.1%)	112,871 (6.5%)	148,974 (8.6%)	593,595 (34.4%)	165,760 (9.6%)	1,723,818 (100.0%)
US	25-44	48,499 (4.3%)	377,446 (33.1%)	61,330 (5.4%)	50,629 (4.4%)	532,719 (46.7%)	69,785 (6.1%)	1,140,408 (100.0%)
US	45-64	4,020 (0.4%)	486,069 (51.8%)	22,134 (2.4%)	9,297 (1.0%)	368,927 (39.3%)	48,359 (5.2%)	938,806 (100.0%)
US	65+	2,695 (0.3%)	408,414 (52.3%)	22,271 (2.9%)	10,470 (1.3%)	270,321 (34.6%)	67,261 (8.6%)	781,432 (100.0%)
**Women**								
US	18-24	98,399 (8.4%)	195,154 (16.6%)	110,145 (9.4%)	130,496 (11.1%)	517,396 (43.9%)	126,106 (10.7%)	1,177,696 (100.0%)
US	25-44	46,662 (5.6%)	70,126 (8.5%)	61,420 (7.4%)	51,607 (6.2%)	510,941 (61.8%)	85,657 (10.4%)	826,413 (100.0%)
US	45-64	8,539 (1.3%)	37,427 (5.9%)	50,335 (7.9%)	16,662 (2.6%)	432,582 (67.8%)	92,299 (14.5%)	637,844 (100.0%)
US	65+	4,656 (0.9%)	32,962 (6.2%)	51,289 (9.6%)	16,154 (3.0%)	305,787 (57.3%)	123,069 (23.1%)	533,917 (100.0%)
Total	-	413,917 (5.3%)	2,109,769 (27.2%)	491,795 (6.3%)	434,289 (5.6%)	3,532,268 (45.5%)	778,296 (10.0%)	7,760,334 (100.0%)

The Facebook impression is number of times the ad was displayed.

Table S7: Facebook impressions by advertisement image and gender and age for the United Kingdom and the United States.

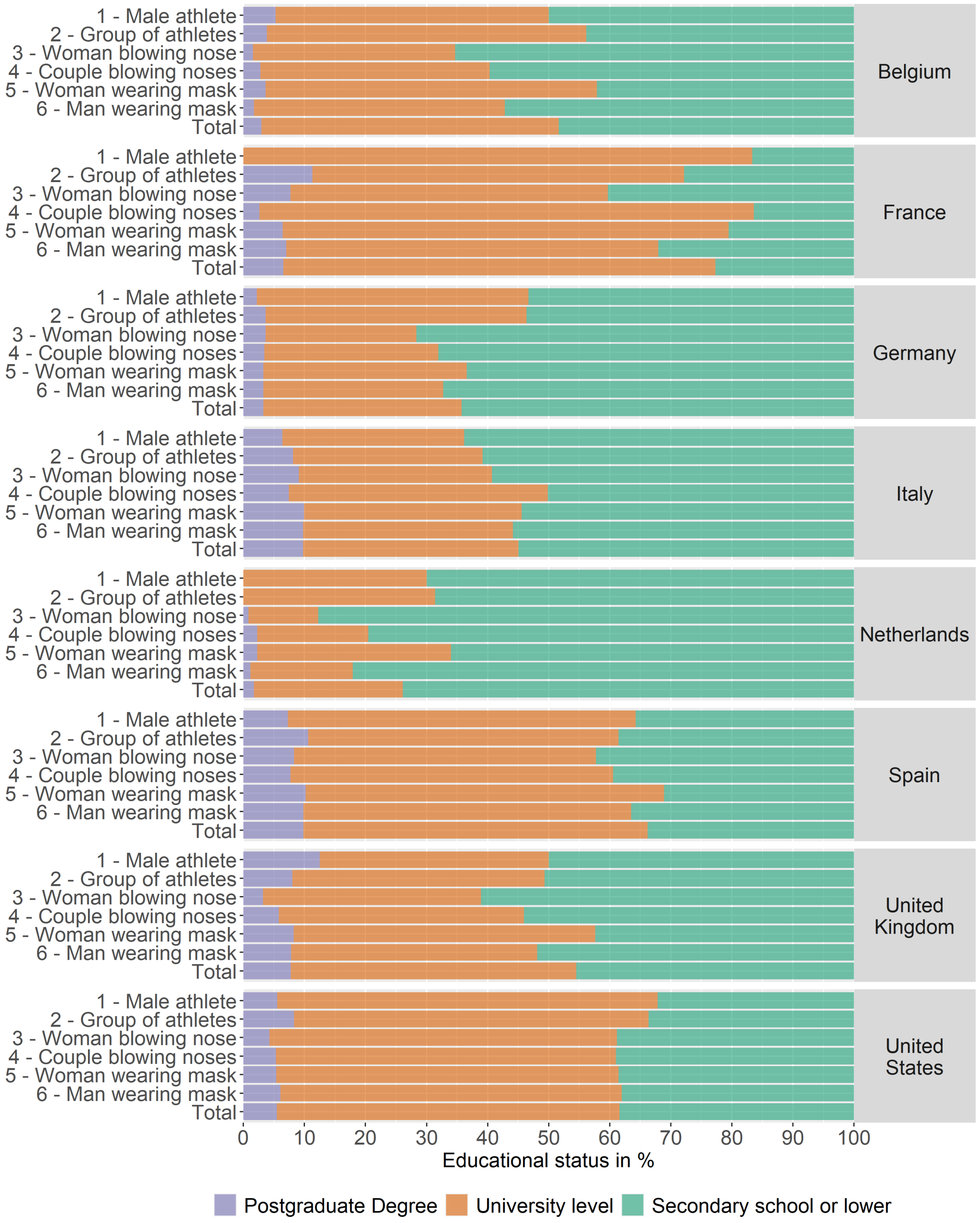

Figure S3: Respondents' educational status by advertisement image and country.

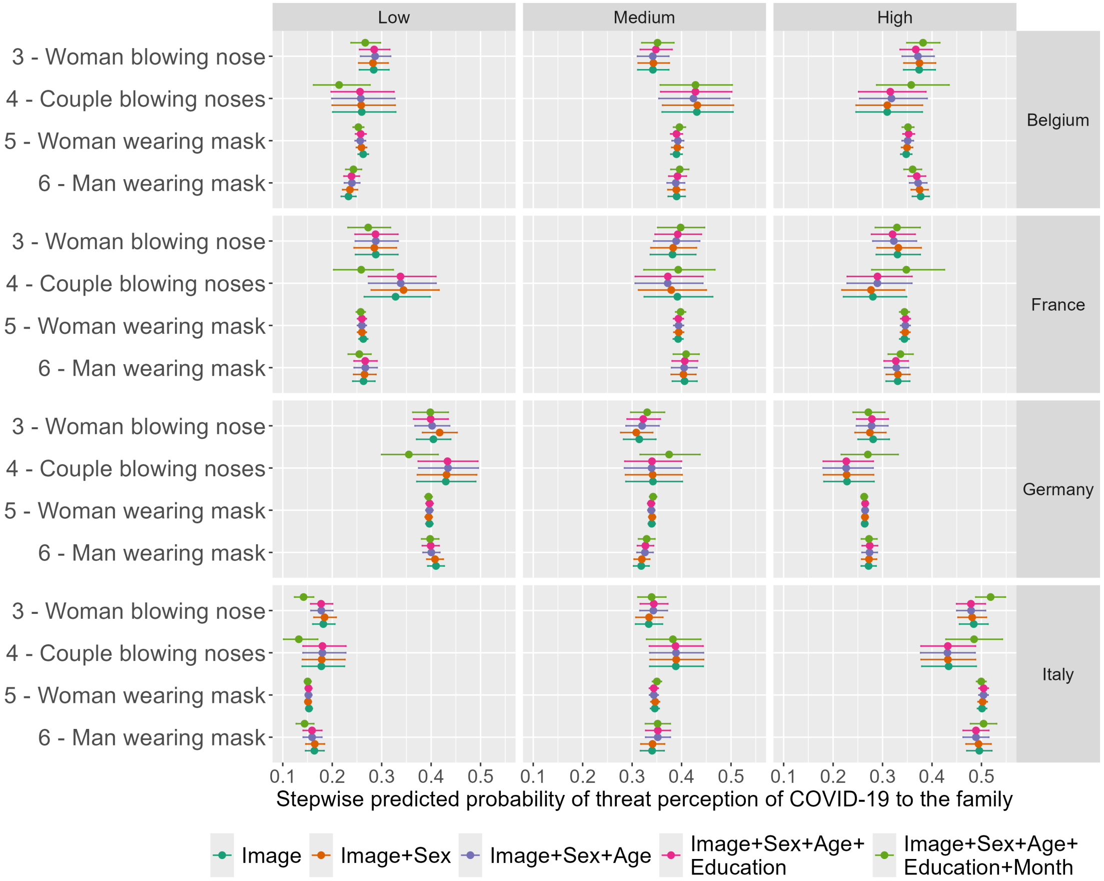

Figure S4: Step-wise predicted probability of threat perception of COVID-19 to the family by advertisement image (on the y-axis) and Belgium, France, Germany and Italy.

(a) Step-wise controlled for sex, age, education, and month of survey participation.

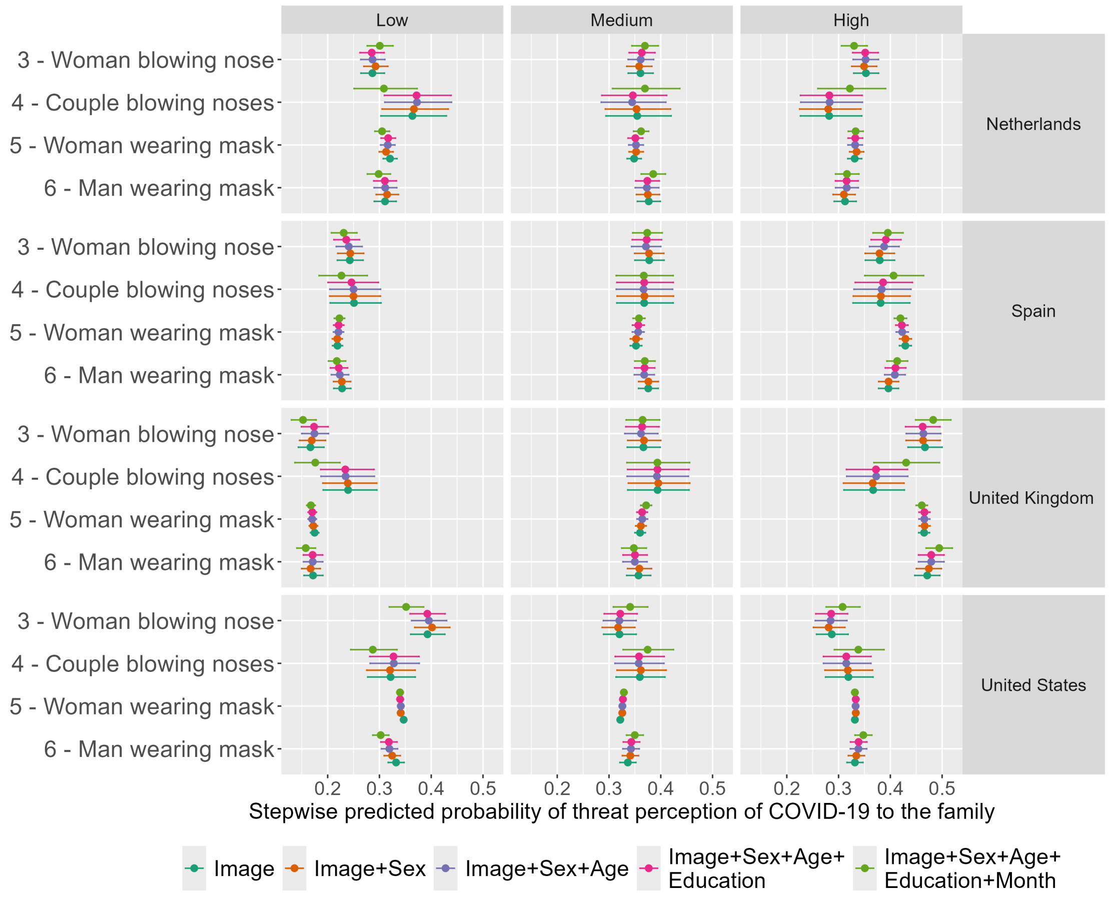

Figure S5: Step-wise predicted probability of threat perception of COVID-19 to the family by advertisement image (on the y-axis) and the Netherlands, Spain, the United Kingdom and the United States.

(a) Step-wise controlled for sex, age, education, and month of survey participation.

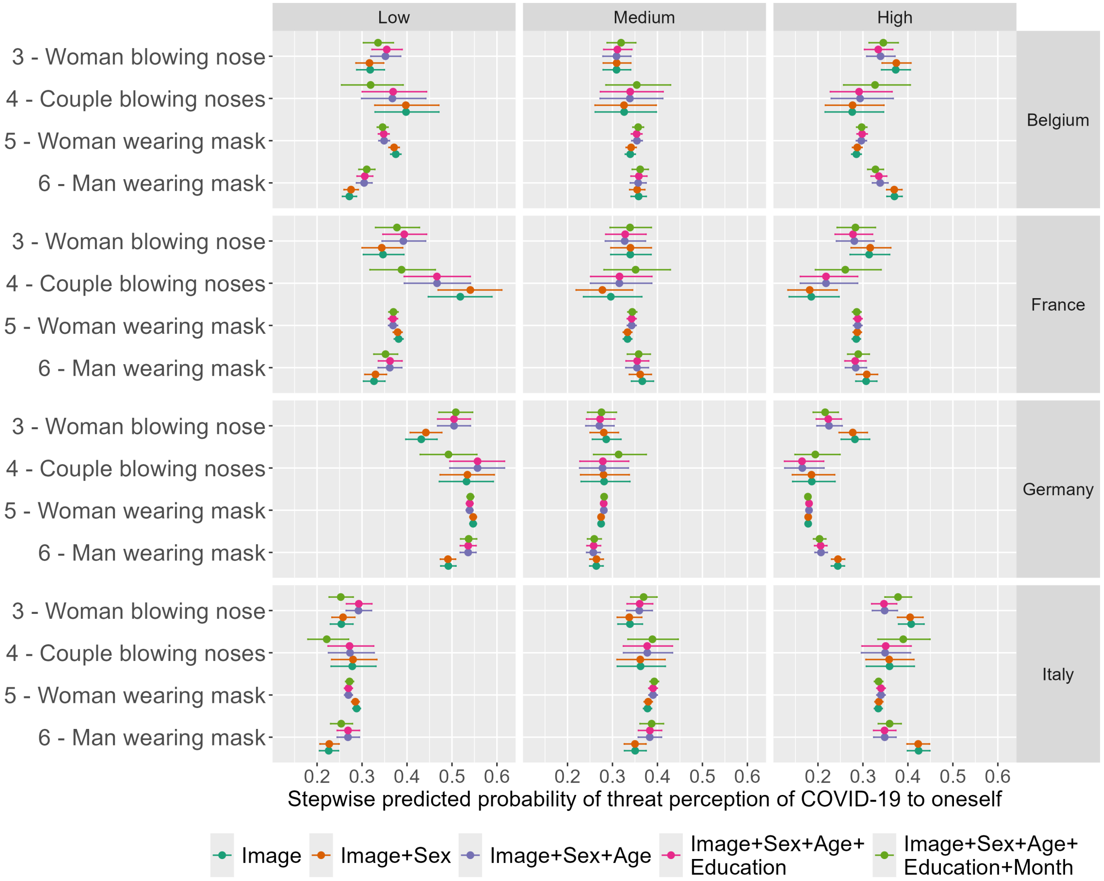

Figure S6: Step-wise predicted probability of threat perception of COVID-19 to the oneself by advertisement image (on the y-axis) and Belgium, France, Germany and Italy.

(a) Step-wise controlled for sex, age, education, and month of survey participation.

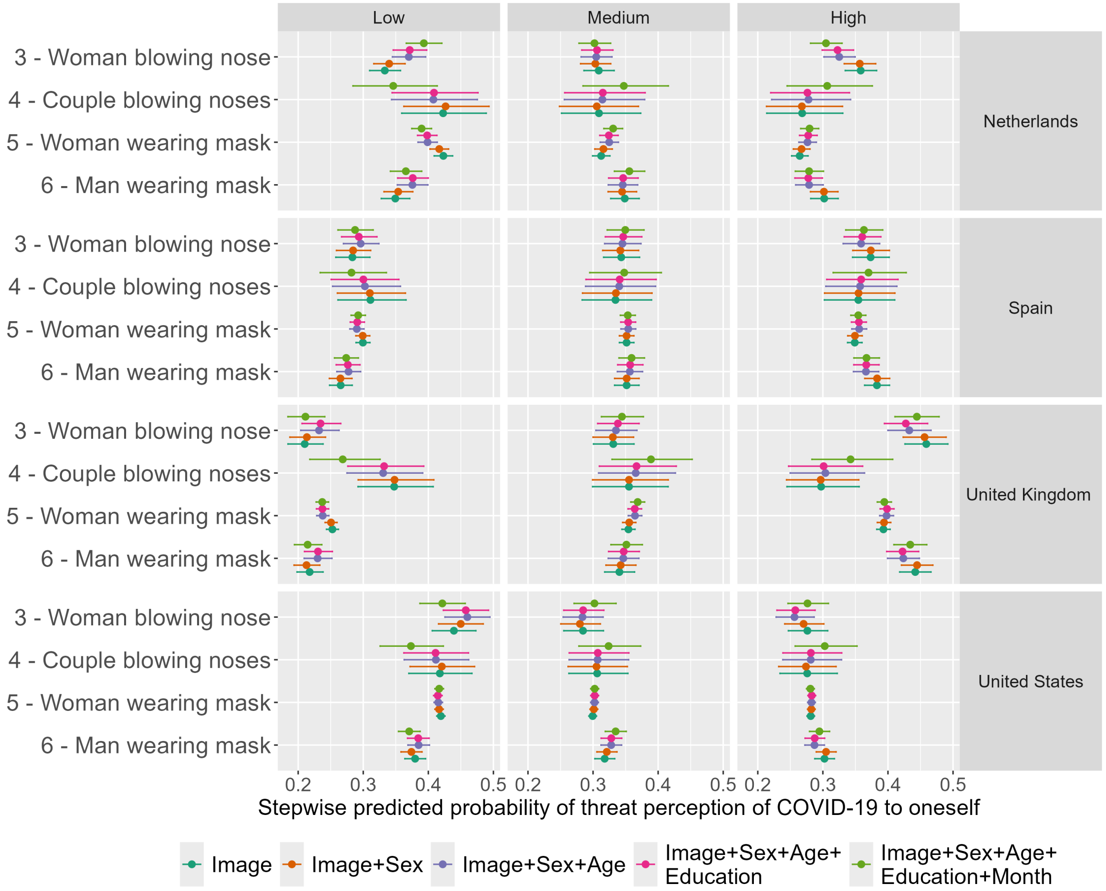

Figure S7: Step-wise predicted probability of threat perception of COVID-19 to the oneself by advertisement image (on the y-axis) and the Netherlands, Spain, the United Kingdom and the United States.

(a) Step-wise controlled for sex, age, education, and month of survey participation.

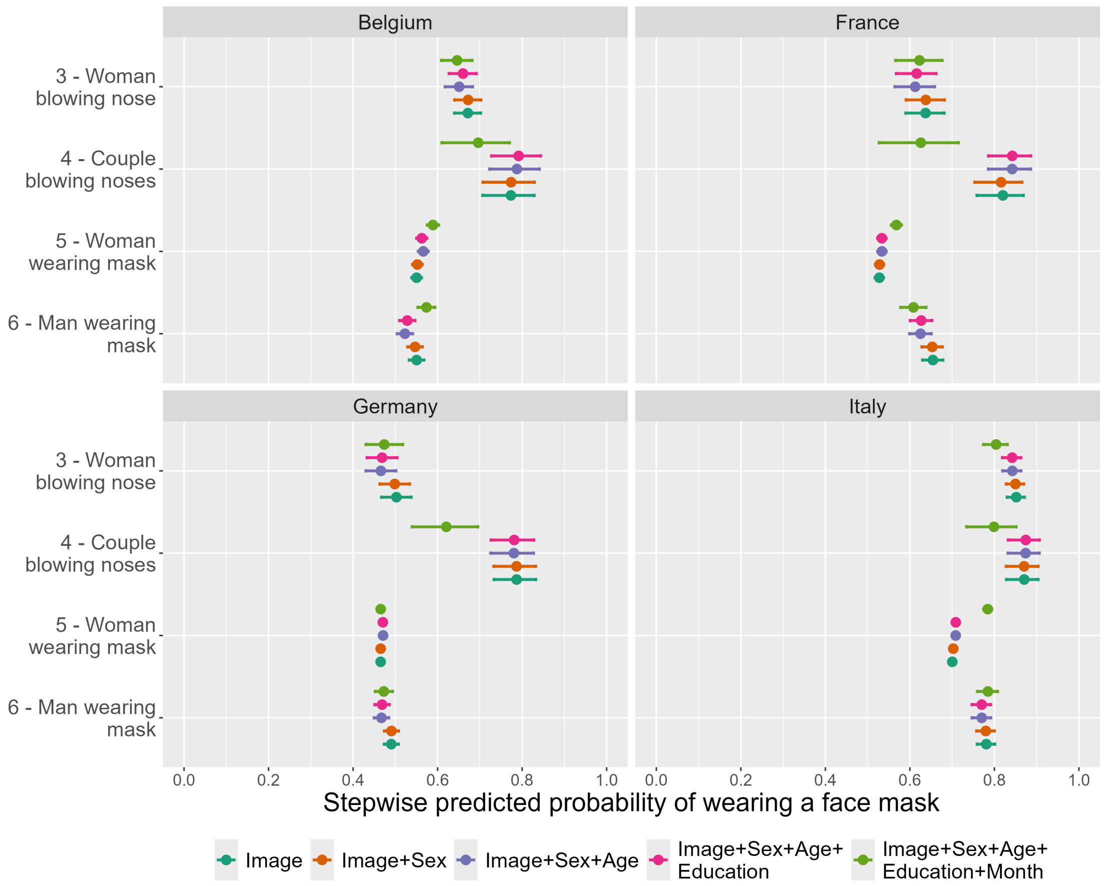

Figure S8: Step-wise predicted probability of wearing a face mask by advertisement image (on the y-axis) and Belgium, France, Germany and Italy.

(a) Step-wise controlled for sex, age, education, and month of survey participation.

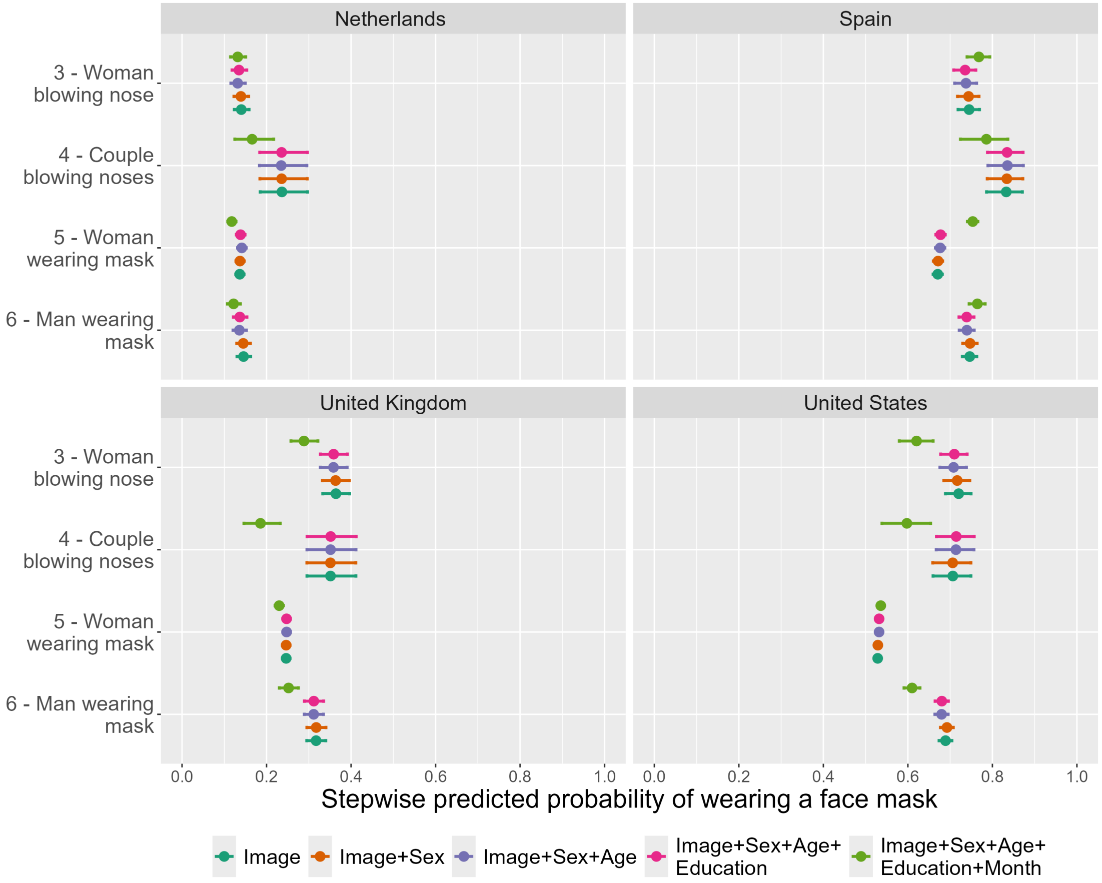

Figure S9: Step-wise predicted probability of wearing a face mask by advertisement image (on the y-axis) and the Netherlands, Spain, the United Kingdom and the United States.

(a) Step-wise controlled for sex, age, education, and month of survey participation.

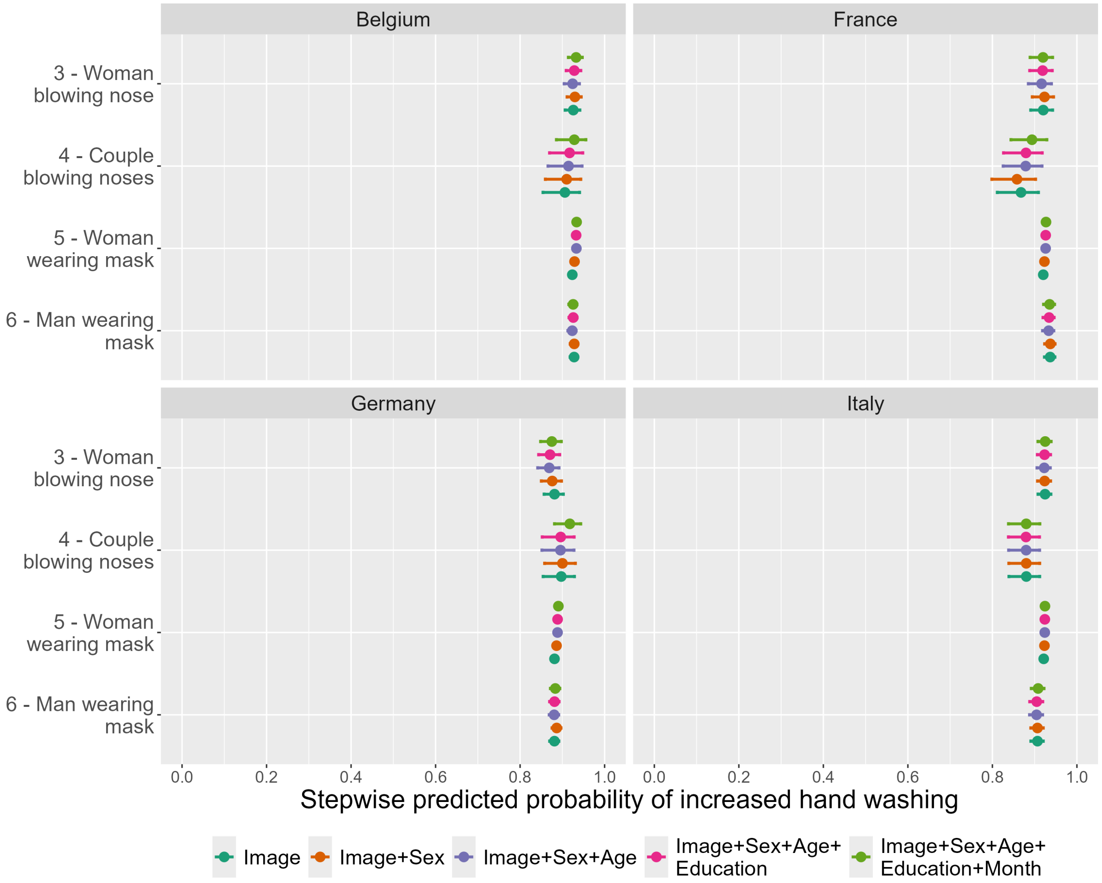

Figure S10: Step-wise predicted probability of increased hand washing by advertisement image (on the y-axis) and Belgium, France, Germany and Italy.

(a) Step-wise controlled for sex, age, education, and month of survey participation.

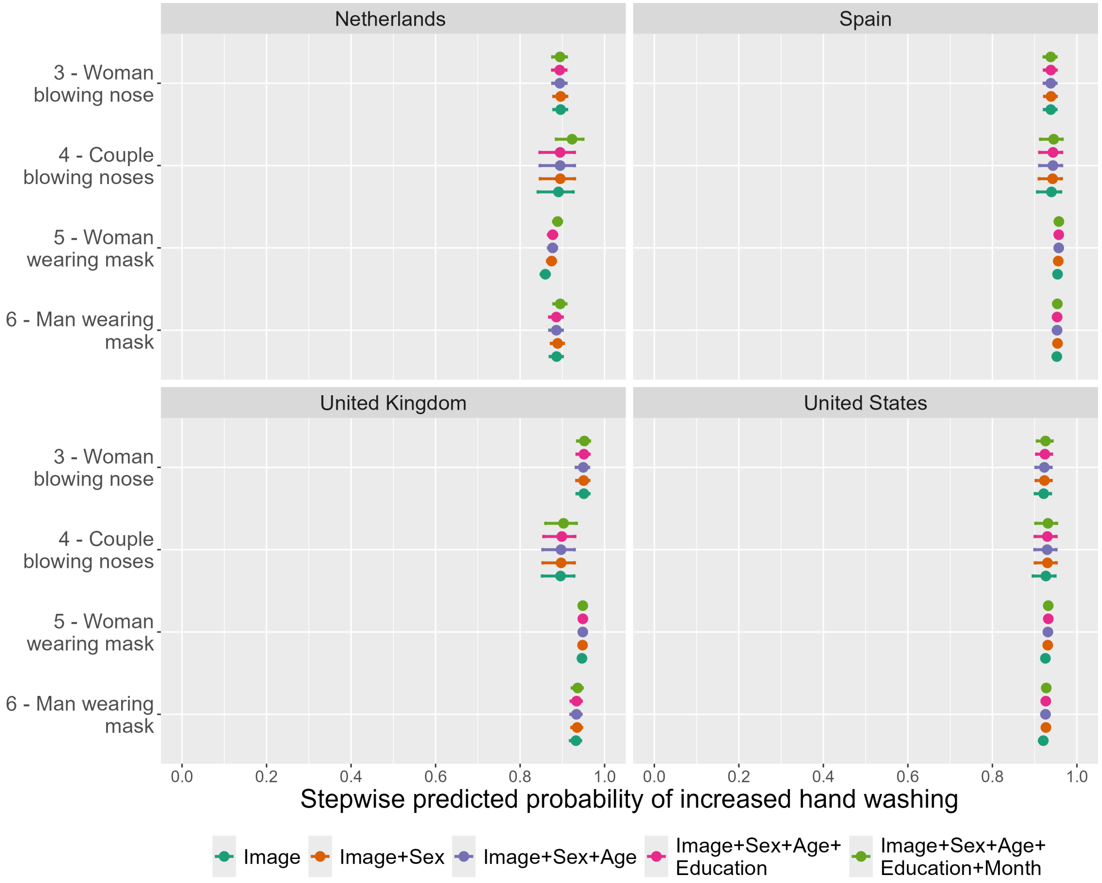

Figure S11: Step-wise predicted probability of increased hand washing by advertisement image (on the y-axis) and the Netherlands, Spain, the United Kingdom and the United States.

(a) Step-wise controlled for sex, age, education, and month of survey participation.
