# Supplementary material for: Assessing self-selection biases in Facebook-recruited online surveys: Evidence from the COVID-19 Health Behavior Survey
Source: PLoS One. 2025 Jul 8;20(7):e0326884. doi: 10.1371/journal.pone.0326884 (PMC12237053; doi:10.1371/journal.pone.0326884)
Supplement: S2 File — (PDF) [file pone.0326884.s002.pdf]

Assessing self-selection biases in online surveys: Evidence from the COVID-19 Health Behavior Survey.
Supporting Information: Regression Tables

List of Tables

Table 1 Step wise regression results for Belgium, Outcome: Wearing a face mask 1
Table 2 Step wise regression results for France, Outcome: Wearing a face mask 2
Table 3 Step wise regression results for Germany, Outcome: Wearing a face mask 3
Table 4 Step wise regression results for Italy, Outcome: Wearing a face mask 4
Table 5 Step wise regression results for the Netherlands, Outcome: Wearing a face mask 5
Table 6 Step wise regression results for Spain, Outcome: Wearing a face mask 6
Table 7 Step wise regression results for the United Kingdom, Outcome: Wearing a face mask 7
Table 8 Step wise regression results for the United States, Outcome: Wearing a face mask 8
Table 9 Step wise regression results for Belgium, Outcome: Increased hand hygiene 9
Table 10 Step wise regression results for France, Outcome: Increased hand hygiene 10
Table 11 Step wise regression results for Germany, Outcome: Increased hand hygiene 11
Table 12 Step wise regression results for Italy, Outcome: Increased hand hygiene 12
Table 13 Step wise regression results for the Netherlands, Outcome: Increased hand hygiene 13
Table 14 Step wise regression results for Spain, Outcome: Increased hand hygiene 14
Table 15 Step wise regression results for the United Kingdom, Outcome: Increased hand hygiene 15
Table 16 Step wise regression results for the United States, Outcome: Increased hand hygiene 16
Table 17 Step wise regression results for Belgium, Outcome: Threat perception of COVID-19 to the family 17
Table 18 Step wise regression results for France, Outcome: Threat perception of COVID-19 to the family 19
Table 19 Step wise regression results for Germany, Outcome: Threat perception of COVID-19 to the family 21
Table 20 Step wise regression results for Italy, Outcome: Threat perception of COVID-19 to the family 22
Table 21 Step wise regression results for the Netherlands, Outcome: Threat perception of COVID-19 to the family. 24
Table 22 Step wise regression results for Spain, Outcome: Threat perception of COVID-19 to the family 26
Table 23 Step wise regression results for the United Kingdom, Outcome: Threat perception of COVID-19 to the family 28
Table 24 Step wise regression results for the United States, Outcome: Threat perception of COVID-19 to the family 30
Table 25 Step wise regression results for Belgium, Outcome: Threat perception of COVID-19 to oneself 31
Table 26 Step wise regression results for France, Outcome: Threat perception of COVID-19 to oneself 33
Table 27 Step wise regression results for Germany, Outcome: Threat perception of COVID-19 to oneself 35
Table 28 Step wise regression results for Italy, Outcome: Threat perception of COVID-19 to oneself 37
Table 29 Step wise regression results for the Netherlands, Outcome: Threat perception of COVID-19 to oneself 39
Table 30 Step wise regression results for Spain, Outcome: Threat perception of COVID-19 to oneself 40
Table 31 Step wise regression results for the United Kingdom, Outcome: Threat perception of COVID-19 to oneself 42
Table 32 Step wise regression results for the United States, Outcome: Threat perception of COVID-19 to oneself ... 44

Table 1 Step wise regression results for Belgium, Outcome: Wearing a face mask

(Intercept)	2.045	2.235	1.182	1.122	0.583
	[1.778, 2.357]	[1.937, 2.585]	[0.990, 1.414]	[0.938, 1.343]	[0.477, 0.712]
	s.e. = 0.147	s.e. = 0.164	s.e. = 0.107	s.e. = 0.103	s.e. = 0.060
	p = <0.001	p = <0.001	p = 0.065	p = 0.210	p = <0.001
Image: Couple blowing nose	1.670	1.669	1.989	1.958	1.254
	[1.157, 2.456]	[1.156, 2.456]	[1.371, 2.941]	[1.349, 2.896]	[0.837, 1.911]
	s.e. = 0.320	s.e. = 0.320	s.e. = 0.386	s.e. = 0.381	s.e. = 0.264
	p = 0.007	p = 0.008	p = <0.001	p = <0.001	p = 0.282
image5 - Woman wearing mask	0.598	0.601	0.699	0.662	0.785
	[0.515, 0.694]	[0.518, 0.697]	[0.600, 0.814]	[0.567, 0.772]	[0.661, 0.930]

	s.e. = 0.045	s.e. = 0.046	s.e. = 0.054	s.e. = 0.052	s.e. = 0.068
	p = <0.001	p = <0.001	p = <0.001	p = <0.001	p = 0.005
Image: Man wearing mask	0.599	0.588	0.587	0.576	0.736
	[0.511, 0.700]	[0.501, 0.688]	[0.500, 0.687]	[0.491, 0.675]	[0.618, 0.877]
	s.e. = 0.048	s.e. = 0.047	s.e. = 0.048	s.e. = 0.047	s.e. = 0.066
	p = <0.001	p = <0.001	p = <0.001	p = <0.001	p = <0.001
Sex: Male		0.777	0.719	0.726	0.622
		[0.718, 0.841]	[0.663, 0.779]	[0.669, 0.787]	[0.568, 0.682]
		s.e. = 0.031	s.e. = 0.030	s.e. = 0.030	s.e. = 0.029
		p = <0.001	p = <0.001	p = <0.001	p = <0.001
Age: 25- 44			1.657	1.577	1.429
			[1.464, 1.876]	[1.392, 1.788]	[1.247, 1.638]
			s.e. = 0.105	s.e. = 0.101	s.e. = 0.100
			p = <0.001	p = <0.001	p = <0.001
Age: 45- 64			1.973	1.931	1.598
			[1.744, 2.233]	[1.706, 2.185]	[1.395, 1.830]
			s.e. = 0.124	s.e. = 0.122	s.e. = 0.111
			p = <0.001	p = <0.001	p = <0.001
Age: 65+			2.322	2.247	1.824
			[2.021, 2.670]	[1.954, 2.585]	[1.563, 2.129]
			s.e. = 0.165	s.e. = 0.160	s.e. = 0.144
			p = <0.001	p = <0.001	p = <0.001
Education: University level				1.250	1.163
				[1.155, 1.353]	[1.066, 1.268]
				s.e. = 0.050	s.e. = 0.052
				p = <0.001	p = <0.001
Education: Postgraduate Degree				1.408	1.405
				[1.116, 1.781]	[1.088, 1.819]
				s.e. = 0.168	s.e. = 0.184
				p = 0.004	p = 0.009
Month: May					4.417
					[3.972, 4.916]
					s.e. = 0.240
					p = <0.001
Month: June					7.779
					[6.338, 9.628]
					s.e. = 0.829
					p = <0.001
Month: July					12.098
					[10.100, 14.597]
					s.e. = 1.136
					p = <0.001
Month: August					14.806
					[10.898, 20.622]
					s.e. = 2.403
					p = <0.001
Num.Obs.	11144	11144	11144	11144	11144
AIC	15191.7	15155.0	15002.2	14971.7	12898.1
Log.Lik.	-7591.835	-7572.479	-7493.075	-7475.865	-6435.042
Odds Ratios and 95% confidence intervals.					

Table 2 Step wise regression results for France, Outcome: Wearing a face mask

(Intercept)	1.755	1.861	1.060	0.973	0.240
	[1.443, 2.142]	[1.528, 2.276]	[0.850, 1.325]	[0.771, 1.231]	[0.180, 0.319]
	s.e. = 0.177	s.e. = 0.189	s.e. = 0.120	s.e. = 0.116	s.e. = 0.035
	p = <0.001	p = <0.001	p = 0.608	p = 0.821	p = <0.001
Image: Couple blowing nose	2.598	2.516	3.372	3.331	1.013
	[1.724, 4.004]	[1.669, 3.879]	[2.225, 5.224]	[2.197, 5.163]	[0.638, 1.640]
	s.e. = 0.557	s.e. = 0.540	s.e. = 0.733	s.e. = 0.724	s.e. = 0.243
	p = <0.001	p = <0.001	p = <0.001	p = <0.001	p = 0.957
image5 - Woman wearing mask	0.637	0.636	0.725	0.713	0.797
	[0.520, 0.778]	[0.519, 0.777]	[0.590, 0.887]	[0.581, 0.873]	[0.626, 1.013]
	s.e. = 0.065	s.e. = 0.065	s.e. = 0.075	s.e. = 0.074	s.e. = 0.098
	p = <0.001	p = <0.001	p = 0.002	p = 0.001	p = 0.065
Image: Man wearing mask	1.080	1.069	1.056	1.047	0.942
	[0.862, 1.350]	[0.853, 1.337]	[0.840, 1.323]	[0.833, 1.312]	[0.721, 1.228]
	s.e. = 0.124	s.e. = 0.123	s.e. = 0.122	s.e. = 0.121	s.e. = 0.128

	p = 0.503	p = 0.559	p = 0.640	p = 0.692	p = 0.658
Sex: Male		0.830	0.796	0.792	0.655
		[0.767, 0.900]	[0.734, 0.863]	[0.730, 0.859]	[0.596, 0.720]
		s.e. = 0.034	s.e. = 0.033	s.e. = 0.033	s.e. = 0.032
		p = <0.001	p = <0.001	p = <0.001	p = <0.001
Age: 25- 44			1.438	1.427	1.321
			[1.285, 1.610]	[1.274, 1.598]	[1.162, 1.503]
			s.e. = 0.083	s.e. = 0.082	s.e. = 0.087
			p = <0.001	p = <0.001	p = <0.001
Age: 45- 64			1.817	1.833	1.701
			[1.626, 2.032]	[1.635, 2.055]	[1.493, 1.939]
			s.e. = 0.104	s.e. = 0.107	s.e. = 0.113
			p = <0.001	p = <0.001	p = <0.001
Age: 65+			2.271	2.289	2.145
			[1.997, 2.583]	[2.007, 2.613]	[1.845, 2.496]
			s.e. = 0.149	s.e. = 0.154	s.e. = 0.166
			p = <0.001	p = <0.001	p = <0.001
Education: University level				1.114	1.042
				[1.015, 1.223]	[0.937, 1.160]
				s.e. = 0.053	s.e. = 0.057
				p = 0.024	p = 0.448
Education: Postgraduate Degree				1.417	1.212
				[1.198, 1.678]	[1.000, 1.472]
				s.e. = 0.122	s.e. = 0.120
				p = <0.001	p = 0.051
Month: April					2.662
					[2.364, 3.001]
					s.e. = 0.162
					p = <0.001
Month: May					13.397
					[11.618, 15.478]
					s.e. = 0.980
					p = <0.001
Month: June					24.230
					[18.905, 31.402]
					s.e. = 3.134
					p = <0.001
Month: July					24.880
					[20.549, 30.276]
					s.e. = 2.459
					p = <0.001
Month: August					19.690
					[15.344, 25.525]
					s.e. = 2.554
					p = <0.001
Num.Obs.	11670	11670	11670	11670	11670
AIC	15896.0	15877.3	15699.5	15686.4	12775.5
Log.Lik.	-7943.998	-7933.627	-7841.769	-7833.218	-6372.756
Odds Ratios and 95% confidence intervals.					

Table 3 Step wise regression results for Germany, Outcome: Wearing a face mask

(Intercept)	1.010	1.045	0.679	0.670	0.131
	[0.878, 1.163]	[0.907, 1.204]	[0.580, 0.795]	[0.572, 0.784]	[0.106, 0.162]
	s.e. = 0.073	s.e. = 0.075	s.e. = 0.055	s.e. = 0.054	s.e. = 0.014
	p = 0.886	p = 0.541	p = <0.001	p = <0.001	p = <0.001
Image: Couple blowing nose	3.659	3.711	4.081	4.051	1.817
	[2.655, 5.111]	[2.692, 5.186]	[2.955, 5.712]	[2.934, 5.671]	[1.256, 2.659]
	s.e. = 0.610	s.e. = 0.619	s.e. = 0.685	s.e. = 0.680	s.e. = 0.347
	p = <0.001	p = <0.001	p = <0.001	p = <0.001	p = 0.002
image5 - Woman wearing mask	0.861	0.875	1.021	1.008	0.967
	[0.746, 0.994]	[0.758, 1.011]	[0.882, 1.183]	[0.870, 1.167]	[0.807, 1.157]
	s.e. = 0.063	s.e. = 0.064	s.e. = 0.076	s.e. = 0.076	s.e. = 0.089
	p = 0.041	p = 0.069	p = 0.779	p = 0.918	p = 0.711
Image: Man wearing mask	0.953	0.969	1.006	1.000	0.996
	[0.815, 1.114]	[0.829, 1.133]	[0.860, 1.177]	[0.854, 1.170]	[0.822, 1.206]
	s.e. = 0.076	s.e. = 0.077	s.e. = 0.081	s.e. = 0.080	s.e. = 0.097
	p = 0.543	p = 0.695	p = 0.941	p = 1.000	p = 0.968

Sex: Male		0.884	0.831	0.827	0.668
		[0.837, 0.932]	[0.786, 0.878]	[0.783, 0.873]	[0.625, 0.713]
		s.e. = 0.024	s.e. = 0.023	s.e. = 0.023	s.e. = 0.022
		p = <0.001	p = <0.001	p = <0.001	p = <0.001
Age: 25- 44			1.313	1.265	1.038
			[1.216, 1.419]	[1.169, 1.368]	[0.947, 1.138]
			s.e. = 0.052	s.e. = 0.051	s.e. = 0.049
			p = <0.001	p = <0.001	p = 0.427
Age: 45- 64			1.543	1.505	1.050
			[1.422, 1.674]	[1.386, 1.634]	[0.952, 1.157]
			s.e. = 0.064	s.e. = 0.063	s.e. = 0.052
			p = <0.001	p = <0.001	p = 0.328
Age: 65+			1.871	1.810	1.604
			[1.695, 2.065]	[1.639, 2.000]	[1.427, 1.804]
			s.e. = 0.094	s.e. = 0.092	s.e. = 0.096
			p = <0.001	p = <0.001	p = <0.001
Education: University level				1.158	1.123
				[1.093, 1.227]	[1.049, 1.202]
				s.e. = 0.034	s.e. = 0.039
				p = <0.001	p = <0.001
Education: Postgraduate Degree				1.229	1.307
				[1.057, 1.429]	[1.095, 1.561]
				s.e. = 0.094	s.e. = 0.118
				p = 0.007	p = 0.003
Month: April					3.868
					[3.461, 4.332]
					s.e. = 0.221
					p = <0.001
Month: May					34.289
					[30.248, 38.956]
					s.e. = 2.213
					p = <0.001
Month: June					30.270
					[25.510, 36.036]
					s.e. = 2.667
					p = <0.001
Month: July					28.114
					[23.913, 33.144]
					s.e. = 2.341
					p = <0.001
Month: August					31.754
					[25.787, 39.319]
					s.e. = 3.416
					p = <0.001
Num.Obs.	22363	22363	22363	22363	22363
AIC	30829.2	30810.8	30637.5	30612.8	23902.3
Log.Lik.	-15410.592	-15400.412	-15310.771	-15296.393	-11936.142
Odds Ratios and 95% confidence intervals.					

Table 4 Step wise regression results for Italy, Outcome: Wearing a face mask

(Intercept)	5.749	6.424	3.650	3.720	1.094
	[4.902, 6.784]	[5.465, 7.597]	[3.042, 4.401]	[3.097, 4.489]	[0.888, 1.352]
	s.e. = 0.476	s.e. = 0.539	s.e. = 0.344	s.e. = 0.352	s.e. = 0.117
	p = <0.001	p = <0.001	p = <0.001	p = <0.001	p = 0.401
Image: Couple blowing nose	1.170	1.185	1.295	1.304	0.969
	[0.816, 1.713]	[0.826, 1.735]	[0.901, 1.900]	[0.907, 1.914]	[0.651, 1.469]
	s.e. = 0.221	s.e. = 0.224	s.e. = 0.246	s.e. = 0.248	s.e. = 0.201
	p = 0.406	p = 0.370	p = 0.174	p = 0.162	p = 0.880
image5 - Woman wearing mask	0.406	0.418	0.454	0.456	0.886
	[0.343, 0.479]	[0.352, 0.492]	[0.382, 0.537]	[0.384, 0.539]	[0.733, 1.067]
	s.e. = 0.035	s.e. = 0.036	s.e. = 0.039	s.e. = 0.040	s.e. = 0.085
	p = <0.001	p = <0.001	p = <0.001	p = <0.001	p = 0.207
Image: Man wearing mask	0.620	0.625	0.626	0.628	0.888
	[0.505, 0.758]	[0.509, 0.765]	[0.509, 0.768]	[0.511, 0.770]	[0.710, 1.109]
	s.e. = 0.064	s.e. = 0.065	s.e. = 0.066	s.e. = 0.066	s.e. = 0.101
	p = <0.001	p = <0.001	p = <0.001	p = <0.001	p = 0.298
Sex: Male		0.683	0.664	0.660	0.712
		[0.633, 0.738]	[0.614, 0.718]	[0.611, 0.714]	[0.654, 0.776]

		s.e. = 0.027	s.e. = 0.026	s.e. = 0.026	s.e. = 0.031
		p = <0.001	p = <0.001	p = <0.001	p = <0.001
Age: 25- 44			1.711	1.770	1.372
			[1.543, 1.897]	[1.592, 1.969]	[1.222, 1.540]
			s.e. = 0.090	s.e. = 0.096	s.e. = 0.081
			p = <0.001	p = <0.001	p = <0.001
Age: 45- 64			2.089	2.137	1.555
			[1.868, 2.335]	[1.909, 2.394]	[1.375, 1.760]
			s.e. = 0.119	s.e. = 0.123	s.e. = 0.098
			p = <0.001	p = <0.001	p = <0.001
Age: 65+			1.808	1.856	1.320
			[1.576, 2.076]	[1.615, 2.135]	[1.132, 1.541]
			s.e. = 0.127	s.e. = 0.132	s.e. = 0.104
			p = <0.001	p = <0.001	p = <0.001
Education: University level				0.912	0.897
				[0.840, 0.991]	[0.820, 0.982]
				s.e. = 0.038	s.e. = 0.041
				p = 0.029	p = 0.018
Education: Postgraduate Degree				0.880	0.857
				[0.772, 1.006]	[0.741, 0.992]
				s.e. = 0.059	s.e. = 0.064
				p = 0.060	p = 0.038
Month: April					4.398
					[3.972, 4.876]
					s.e. = 0.230
					p = <0.001
Month: May					13.671
					[11.539, 16.311]
					s.e. = 1.206
					p = <0.001
Month: June					17.197
					[11.851, 26.072]
					s.e. = 3.445
					p = <0.001
Month: July					17.721
					[12.813, 25.328]
					s.e. = 3.071
					p = <0.001
Month: August					23.412
					[13.735, 44.215]
					s.e. = 6.917
					p = <0.001
Num.Obs.	14202	14202	14202	14202	14202
AIC	16517.1	16426.3	16257.9	16255.4	13778.5
Log.Lik.	-8254.570	-8208.129	-8120.960	-8117.692	-6874.242
Odds Ratios and 95% confidence intervals.					

Table 5 Step wise regression results for the Netherlands, Outcome: Wearing a face mask

(Intercept)	0.163	0.167	0.121	0.116	0.089
	[0.141, 0.188]	[0.144, 0.194]	[0.093, 0.157]	[0.088, 0.151]	[0.067, 0.117]
	s.e. = 0.012	s.e. = 0.013	s.e. = 0.016	s.e. = 0.016	s.e. = 0.013
	p = <0.001	p = <0.001	p = <0.001	p = <0.001	p = <0.001
Image: Couple blowing nose	1.898	1.905	2.023	1.978	1.310
	[1.337, 2.659]	[1.342, 2.669]	[1.421, 2.843]	[1.388, 2.784]	[0.904, 1.876]
	s.e. = 0.332	s.e. = 0.334	s.e. = 0.357	s.e. = 0.351	s.e. = 0.243
	p = <0.001	p = <0.001	p = <0.001	p = <0.001	p = 0.146
image5 - Woman wearing mask	0.970	0.981	1.086	1.032	0.878
	[0.822, 1.149]	[0.831, 1.163]	[0.914, 1.295]	[0.866, 1.233]	[0.732, 1.056]
	s.e. = 0.083	s.e. = 0.084	s.e. = 0.097	s.e. = 0.093	s.e. = 0.082
	p = 0.722	p = 0.825	p = 0.352	p = 0.730	p = 0.165
Image: Man wearing mask	1.044	1.047	1.036	1.017	0.915
	[0.860, 1.270]	[0.862, 1.272]	[0.852, 1.260]	[0.836, 1.238]	[0.745, 1.125]
	s.e. = 0.104	s.e. = 0.104	s.e. = 0.103	s.e. = 0.102	s.e. = 0.096
	p = 0.663	p = 0.647	p = 0.726	p = 0.867	p = 0.399
Sex: Male		0.917	0.872	0.855	0.780
		[0.804, 1.044]	[0.764, 0.995]	[0.748, 0.976]	[0.679, 0.894]

		s.e. = 0.061	s.e. = 0.059	s.e. = 0.058	s.e. = 0.055
		p = 0.193	p = 0.043	p = 0.021	p = <0.001
Age: 25- 44			1.165	1.104	0.916
			[0.907, 1.506]	[0.858, 1.429]	[0.706, 1.196]
			s.e. = 0.150	s.e. = 0.143	s.e. = 0.123
			p = 0.237	p = 0.447	p = 0.515
Age: 45- 64			1.283	1.307	1.000
			[1.017, 1.631]	[1.035, 1.664]	[0.786, 1.282]
			s.e. = 0.154	s.e. = 0.158	s.e. = 0.125
			p = 0.039	p = 0.027	p = 0.999
Age: 65+			1.669	1.700	1.299
			[1.310, 2.142]	[1.334, 2.185]	[1.012, 1.680]
			s.e. = 0.209	s.e. = 0.214	s.e. = 0.168
			p = <0.001	p = <0.001	p = 0.043
Education: University level				1.312	1.154
				[1.131, 1.520]	[0.989, 1.344]
				s.e. = 0.099	s.e. = 0.090
				p = <0.001	p = 0.067
Education: Postgraduate Degree				1.940	1.645
				[1.274, 2.877]	[1.062, 2.484]
				s.e. = 0.402	s.e. = 0.355
				p = 0.001	p = 0.021
Month: May					2.368
					[2.012, 2.788]
					s.e. = 0.197
					p = <0.001
Month: June					4.197
					[3.357, 5.229]
					s.e. = 0.474
					p = <0.001
Month: July					5.516
					[4.554, 6.675]
					s.e. = 0.538
					p = <0.001
Month: August					6.899
					[5.215, 9.089]
					s.e. = 0.977
					p = <0.001
Num.Obs.	8223	8223	8223	8223	8223
AIC	6709.9	6710.2	6691.9	6676.2	6230.6
Log.Lik.	-3350.938	-3350.084	-3337.940	-3328.080	-3101.301
Odds Ratios and 95% confidence intervals.					

Table 6 Step wise regression results for Spain, Outcome: Wearing a face mask

(Intercept)	2.920	3.135	2.160	2.371	0.934
	[2.559, 3.343]	[2.739, 3.598]	[1.751, 2.670]	[1.910, 2.951]	[0.731, 1.195]
	s.e. = 0.199	s.e. = 0.218	s.e. = 0.232	s.e. = 0.263	s.e. = 0.117
	p = <0.001	p = <0.001	p = <0.001	p = <0.001	p = 0.585
Image: Couple blowing nose	1.705	1.733	1.808	1.817	1.108
	[1.233, 2.398]	[1.253, 2.438]	[1.306, 2.546]	[1.312, 2.558]	[0.773, 1.608]
	s.e. = 0.289	s.e. = 0.294	s.e. = 0.307	s.e. = 0.309	s.e. = 0.206
	p = 0.002	p = 0.001	p = <0.001	p = <0.001	p = 0.583
image5 - Woman wearing mask	0.698	0.704	0.744	0.755	0.923
	[0.604, 0.803]	[0.610, 0.811]	[0.643, 0.859]	[0.652, 0.872]	[0.786, 1.082]
	s.e. = 0.051	s.e. = 0.051	s.e. = 0.055	s.e. = 0.056	s.e. = 0.075
	p = <0.001	p = <0.001	p = <0.001	p = <0.001	p = 0.326
Image: Man wearing mask	1.008	1.019	1.011	1.019	0.980
	[0.857, 1.183]	[0.866, 1.197]	[0.859, 1.188]	[0.866, 1.197]	[0.820, 1.170]
	s.e. = 0.083	s.e. = 0.084	s.e. = 0.084	s.e. = 0.084	s.e. = 0.089
	p = 0.926	p = 0.820	p = 0.895	p = 0.820	p = 0.825
Sex: Male		0.783	0.750	0.741	0.762
		[0.718, 0.854]	[0.686, 0.820]	[0.677, 0.810]	[0.692, 0.840]
		s.e. = 0.035	s.e. = 0.034	s.e. = 0.034	s.e. = 0.038
		p = <0.001	p = <0.001	p = <0.001	p = <0.001
Age: 25- 44			1.304	1.309	1.083
			[1.092, 1.555]	[1.096, 1.561]	[0.895, 1.310]
			s.e. = 0.117	s.e. = 0.118	s.e. = 0.105
			p = 0.003	p = 0.003	p = 0.410

Age: 45- 64			1.525	1.516	1.182
			[1.279, 1.817]	[1.270, 1.806]	[0.977, 1.427]
			s.e. = 0.137	s.e. = 0.136	s.e. = 0.114
			p = <0.001	p = <0.001	p = 0.084
Age: 65+			1.617	1.627	1.276
			[1.325, 1.972]	[1.333, 1.985]	[1.028, 1.582]
			s.e. = 0.164	s.e. = 0.165	s.e. = 0.140
			p = <0.001	p = <0.001	p = 0.027
Education: University level				0.850	0.866
				[0.776, 0.931]	[0.784, 0.955]
				s.e. = 0.040	s.e. = 0.044
				p = <0.001	p = 0.004
Education: Postgraduate Degree				0.924	0.935
				[0.795, 1.075]	[0.794, 1.101]
				s.e. = 0.071	s.e. = 0.078
				p = 0.304	p = 0.417
Month: April					1.963
					[1.762, 2.187]
					s.e. = 0.108
					p = <0.001
Month: May					13.528
					[11.296, 16.285]
					s.e. = 1.262
					p = <0.001
Month: June					35.710
					[22.770, 59.840]
					s.e. = 8.751
					p = <0.001
Month: July					23.770
					[16.655, 35.198]
					s.e. = 4.522
					p = <0.001
Month: August					17.868
					[11.037, 31.110]
					s.e. = 4.693
					p = <0.001
Num.Obs.	10964	10964	10964	10964	10964
AIC	13310.6	13282.4	13257.0	13248.7	11454.3
Log.Lik.	-6651.308	-6636.213	-6620.518	-6614.348	-5712.147
Odds Ratios and 95% confidence intervals.					

Table 7 Step wise regression results for the United Kingdom, Outcome: Wearing a face mask

(Intercept)	0.573	0.580	0.470	0.463	0.204
	[0.499, 0.657]	[0.504, 0.666]	[0.374, 0.588]	[0.368, 0.580]	[0.158, 0.264]
	s.e. = 0.040	s.e. = 0.041	s.e. = 0.054	s.e. = 0.054	s.e. = 0.027
	p = <0.001	p = <0.001	p = <0.001	p = <0.001	p = <0.001
Image: Couple blowing nose	0.946	0.948	0.970	0.970	0.562
	[0.706, 1.261]	[0.708, 1.264]	[0.724, 1.295]	[0.724, 1.295]	[0.407, 0.772]
	s.e. = 0.140	s.e. = 0.140	s.e. = 0.144	s.e. = 0.144	s.e. = 0.092
	p = 0.706	p = 0.718	p = 0.838	p = 0.839	p = <0.001
image5 - Woman wearing mask	0.571	0.573	0.589	0.588	0.736
	[0.494, 0.661]	[0.495, 0.663]	[0.508, 0.683]	[0.507, 0.682]	[0.625, 0.868]
	s.e. = 0.043	s.e. = 0.043	s.e. = 0.044	s.e. = 0.044	s.e. = 0.061
	p = <0.001	p = <0.001	p = <0.001	p = <0.001	p = <0.001
Image: Man wearing mask	0.811	0.815	0.810	0.810	0.831
	[0.683, 0.964]	[0.686, 0.969]	[0.681, 0.963]	[0.681, 0.964]	[0.687, 1.005]
	s.e. = 0.071	s.e. = 0.072	s.e. = 0.072	s.e. = 0.072	s.e. = 0.081
	p = 0.018	p = 0.020	p = 0.017	p = 0.017	p = 0.056
Sex: Male		0.956	0.948	0.949	0.907
		[0.876, 1.043]	[0.868, 1.035]	[0.869, 1.036]	[0.822, 1.000]
		s.e. = 0.043	s.e. = 0.043	s.e. = 0.043	s.e. = 0.045
		p = 0.313	p = 0.232	p = 0.241	p = 0.050
Age: 25- 44			1.160	1.160	1.013
			[0.954, 1.415]	[0.953, 1.417]	[0.819, 1.257]
			s.e. = 0.117	s.e. = 0.117	s.e. = 0.111
			p = 0.141	p = 0.142	p = 0.909

Age: 45- 64			1.204	1.209	0.836
			[1.001, 1.454]	[1.005, 1.461]	[0.684, 1.027]
			s.e. = 0.115	s.e. = 0.115	s.e. = 0.087
			p = 0.051	p = 0.047	p = 0.084
Age: 65+			1.317	1.323	0.848
			[1.089, 1.600]	[1.094, 1.607]	[0.689, 1.048]
			s.e. = 0.129	s.e. = 0.130	s.e. = 0.091
			p = 0.005	p = 0.004	p = 0.124
Education: University level				1.037	0.965
				[0.949, 1.133]	[0.875, 1.064]
				s.e. = 0.047	s.e. = 0.048
				p = 0.420	p = 0.475
Education: Postgraduate Degree				0.956	0.880
				[0.808, 1.128]	[0.730, 1.058]
				s.e. = 0.081	s.e. = 0.083
				p = 0.597	p = 0.177
Month: April					2.302
					[2.030, 2.612]
					s.e. = 0.148
					p = <0.001
Month: May					4.698
					[4.137, 5.338]
					s.e. = 0.305
					p = <0.001
Month: June					9.798
					[8.096, 11.873]
					s.e. = 0.957
					p = <0.001
Month: July					19.603
					[16.351, 23.577]
					s.e. = 1.830
					p = <0.001
Month: August					32.779
					[23.725, 46.168]
					s.e. = 5.555
					p = <0.001
Num.Obs.	11401	11401	11401	11401	11401
AIC	13173.0	13174.0	13170.1	13172.8	11254.3
Log.Lik.	-6582.521	-6582.010	-6577.044	-6576.403	-5612.153
Odds Ratios and 95% confidence intervals.					

Table 8 Step wise regression results for the United States, Outcome: Wearing a face mask

(Intercept)	2.574	3.044	1.860	1.660	0.232
	[2.218, 2.998]	[2.617, 3.552]	[1.566, 2.215]	[1.396, 1.980]	[0.189, 0.285]
	s.e. = 0.198	s.e. = 0.237	s.e. = 0.164	s.e. = 0.148	s.e. = 0.024
	p = <0.001	p = <0.001	p = <0.001	p = <0.001	p = <0.001
Image: Couple blowing nose	0.934	0.949	1.028	1.022	0.908
	[0.719, 1.218]	[0.729, 1.240]	[0.788, 1.345]	[0.783, 1.338]	[0.679, 1.220]
	s.e. = 0.126	s.e. = 0.129	s.e. = 0.140	s.e. = 0.139	s.e. = 0.136
	p = 0.613	p = 0.699	p = 0.840	p = 0.873	p = 0.520
image5 - Woman wearing mask	0.435	0.444	0.468	0.464	0.706
	[0.373, 0.507]	[0.380, 0.517]	[0.401, 0.546]	[0.397, 0.541]	[0.593, 0.838]
	s.e. = 0.034	s.e. = 0.035	s.e. = 0.037	s.e. = 0.037	s.e. = 0.062
	p = <0.001	p = <0.001	p = <0.001	p = <0.001	p = <0.001
Image: Man wearing mask	0.861	0.890	0.875	0.870	0.956
	[0.728, 1.016]	[0.752, 1.051]	[0.739, 1.034]	[0.734, 1.028]	[0.792, 1.151]
	s.e. = 0.073	s.e. = 0.076	s.e. = 0.075	s.e. = 0.075	s.e. = 0.091
	p = 0.079	p = 0.172	p = 0.120	p = 0.104	p = 0.638
Sex: Male		0.597	0.573	0.582	0.497
		[0.568, 0.627]	[0.545, 0.602]	[0.554, 0.612]	[0.469, 0.527]
		s.e. = 0.015	s.e. = 0.015	s.e. = 0.015	s.e. = 0.015
		p = <0.001	p = <0.001	p = <0.001	p = <0.001
Age: 25- 44			1.450	1.335	0.977
			[1.317, 1.596]	[1.211, 1.471]	[0.871, 1.095]
			s.e. = 0.071	s.e. = 0.066	s.e. = 0.057
			p = <0.001	p = <0.001	p = 0.685

Age: 45- 64			1.559	1.469	0.911
			[1.422, 1.710]	[1.339, 1.613]	[0.816, 1.017]
			s.e. = 0.074	s.e. = 0.070	s.e. = 0.051
			p = <0.001	p = <0.001	p = 0.096
Age: 65+			1.969	1.831	1.190
			[1.791, 2.164]	[1.665, 2.015]	[1.063, 1.331]
			s.e. = 0.095	s.e. = 0.089	s.e. = 0.068
			p = <0.001	p = <0.001	p = 0.002
Education: University level				1.325	1.407
				[1.260, 1.393]	[1.329, 1.490]
				s.e. = 0.034	s.e. = 0.041
				p = <0.001	p = <0.001
Education: Postgraduate Degree				1.530	1.837
				[1.369, 1.712]	[1.613, 2.093]
				s.e. = 0.087	s.e. = 0.122
				p = <0.001	p = <0.001
Month: April					9.633
					[8.895, 10.441]
					s.e. = 0.394
					p = <0.001
Month: May					16.063
					[14.731, 17.530]
					s.e. = 0.713
					p = <0.001
Month: June					17.147
					[14.824, 19.879]
					s.e. = 1.283
					p = <0.001
Month: July					21.363
					[19.044, 23.995]
					s.e. = 1.259
					p = <0.001
Month: August					21.786
					[17.985, 26.518]
					s.e. = 2.157
					p = <0.001
Num.Obs.	28400	28400	28400	28400	28400
AIC	38539.8	38119.3	37901.3	37763.9	31133.5
Log.Lik.	-19265.922	-19054.668	-18942.656	-18871.971	-15551.747
Odds Ratios and 95% confidence intervals.					

Table 9 Step wise regression results for Belgium, Outcome: Increased hand hygiene

(Intercept)	12.492	17.045	10.409	9.821	11.657
	[9.788, 16.236]	[13.206, 22.382]	[7.653, 14.361]	[7.204, 13.578]	[8.482, 16.238]
	s.e. = 1.610	s.e. = 2.291	s.e. = 1.670	s.e. = 1.587	s.e. = 1.929
	p = <0.001	p = <0.001	p = <0.001	p = <0.001	p = <0.001
Image: Couple blowing nose	0.772	0.766	0.877	0.855	0.937
	[0.451, 1.391]	[0.446, 1.384]	[0.509, 1.590]	[0.496, 1.551]	[0.541, 1.702]
	s.e. = 0.221	s.e. = 0.220	s.e. = 0.254	s.e. = 0.248	s.e. = 0.272
	p = 0.365	p = 0.354	p = 0.650	p = 0.589	p = 0.822
image5 - Woman wearing mask	0.966	0.986	1.144	1.069	1.020
	[0.733, 1.253]	[0.747, 1.281]	[0.862, 1.496]	[0.804, 1.401]	[0.766, 1.338]
	s.e. = 0.132	s.e. = 0.135	s.e. = 0.161	s.e. = 0.151	s.e. = 0.145
	p = 0.803	p = 0.918	p = 0.339	p = 0.637	p = 0.890
Image: Man wearing mask	1.030	0.976	0.987	0.965	0.899
	[0.767, 1.363]	[0.727, 1.295]	[0.734, 1.310]	[0.717, 1.281]	[0.667, 1.195]
	s.e. = 0.151	s.e. = 0.144	s.e. = 0.146	s.e. = 0.142	s.e. = 0.133
	p = 0.842	p = 0.871	p = 0.930	p = 0.808	p = 0.473
Sex: Male		0.475	0.442	0.449	0.456
		[0.412, 0.548]	[0.383, 0.511]	[0.388, 0.518]	[0.394, 0.527]
		s.e. = 0.034	s.e. = 0.033	s.e. = 0.033	s.e. = 0.034
		p = <0.001	p = <0.001	p = <0.001	p = <0.001
Age: 25- 44			1.382	1.293	1.332
			[1.115, 1.707]	[1.041, 1.602]	[1.071, 1.652]
			s.e. = 0.150	s.e. = 0.142	s.e. = 0.147
			p = 0.003	p = 0.019	p = 0.010
Age: 45- 64			1.747	1.694	1.792

			[1.407, 2.163]	[1.363, 2.098]	[1.440, 2.224]
			s.e. = 0.192	s.e. = 0.186	s.e. = 0.199
			p = <0.001	p = <0.001	p = <0.001
Age: 65+			1.971	1.882	1.998
			[1.538, 2.528]	[1.466, 2.416]	[1.555, 2.569]
			s.e. = 0.250	s.e. = 0.240	s.e. = 0.256
			p = <0.001	p = <0.001	p = <0.001
Education: University level				1.328	1.366
				[1.146, 1.540]	[1.177, 1.585]
				s.e. = 0.100	s.e. = 0.104
				p = <0.001	p = <0.001
Education: Postgraduate Degree				1.590	1.630
				[1.013, 2.650]	[1.037, 2.720]
				s.e. = 0.388	s.e. = 0.399
				p = 0.057	p = 0.046
Month: May					0.707
					[0.593, 0.846]
					s.e. = 0.064
					p = <0.001
Month: June					0.645
					[0.490, 0.863]
					s.e. = 0.093
					p = 0.002
Month: July					0.714
					[0.570, 0.900]
					s.e. = 0.083
					p = 0.004
Month: August					0.479
					[0.356, 0.657]
					s.e. = 0.075
					p = <0.001
Num.Obs.	11144	11144	11144	11144	11144
AIC	5964.8	5862.7	5834.3	5822.2	5795.0
Log.Lik.	-2978.404	-2926.366	-2909.171	-2901.093	-2883.484
Odds Ratios and 95% confidence intervals.					

Table 10 Step wise regression results for France, Outcome: Increased hand hygiene

(Intercept)	11.559	14.403	8.202	7.037	9.041
	[8.275, 16.713]	[10.245, 20.941]	[5.641, 12.277]	[4.734, 10.740]	[5.894, 14.213]
	s.e. = 2.066	s.e. = 2.619	s.e. = 1.623	s.e. = 1.467	s.e. = 2.027
	p = <0.001	p = <0.001	p = <0.001	p = <0.001	p = <0.001
Image: Couple blowing nose	0.568	0.503	0.662	0.644	0.733
	[0.329, 0.989]	[0.291, 0.879]	[0.380, 1.164]	[0.370, 1.134]	[0.417, 1.301]
	s.e. = 0.159	s.e. = 0.141	s.e. = 0.188	s.e. = 0.183	s.e. = 0.212
	p = 0.043	p = 0.014	p = 0.146	p = 0.122	p = 0.282
image5 - Woman wearing mask	1.000	0.999	1.141	1.106	1.105
	[0.687, 1.409]	[0.685, 1.408]	[0.781, 1.615]	[0.756, 1.568]	[0.753, 1.570]
	s.e. = 0.183	s.e. = 0.183	s.e. = 0.211	s.e. = 0.205	s.e. = 0.206
	p = 1.000	p = 0.994	p = 0.475	p = 0.587	p = 0.594
Image: Man wearing mask	1.280	1.238	1.275	1.253	1.262
	[0.841, 1.906]	[0.812, 1.846]	[0.835, 1.907]	[0.819, 1.874]	[0.823, 1.891]
	s.e. = 0.266	s.e. = 0.259	s.e. = 0.268	s.e. = 0.264	s.e. = 0.267
	p = 0.235	p = 0.306	p = 0.247	p = 0.284	p = 0.272
Sex: Male		0.550	0.529	0.530	0.540
		[0.479, 0.631]	[0.461, 0.609]	[0.461, 0.609]	[0.470, 0.622]
		s.e. = 0.039	s.e. = 0.038	s.e. = 0.038	s.e. = 0.039
		p = <0.001	p = <0.001	p = <0.001	p = <0.001
Age: 25- 44			1.551	1.549	1.592
			[1.287, 1.868]	[1.284, 1.867]	[1.318, 1.921]
			s.e. = 0.147	s.e. = 0.148	s.e. = 0.153
			p = <0.001	p = <0.001	p = <0.001
Age: 45- 64			1.893	1.955	2.025
			[1.566, 2.288]	[1.607, 2.377]	[1.661, 2.468]
			s.e. = 0.183	s.e. = 0.195	s.e. = 0.204
			p = <0.001	p = <0.001	p = <0.001
Age: 65+			1.992	2.059	2.157
			[1.592, 2.502]	[1.634, 2.603]	[1.709, 2.733]
			s.e. = 0.230	s.e. = 0.244	s.e. = 0.258

			p = <0.001	p = <0.001	p = <0.001
Education: University level				1.219	1.237
				[1.029, 1.441]	[1.043, 1.462]
				s.e. = 0.105	s.e. = 0.107
				p = 0.021	p = 0.014
Education: Postgraduate Degree				1.564	1.627
				[1.128, 2.215]	[1.173, 2.307]
				s.e. = 0.268	s.e. = 0.280
				p = 0.009	p = 0.005
Month: April					0.777
					[0.628, 0.957]
					s.e. = 0.083
					p = 0.019
Month: May					0.669
					[0.530, 0.842]
					s.e. = 0.079
					p = <0.001
Month: June					0.723
					[0.517, 1.026]
					s.e. = 0.126
					p = 0.063
Month: July					0.647
					[0.496, 0.844]
					s.e. = 0.088
					p = 0.001
Month: August					0.449
					[0.328, 0.619]
					s.e. = 0.072
					p = <0.001
Num.Obs.	11670	11670	11670	11670	11670
AIC	6411.6	6343.9	6298.9	6293.7	6275.3
Log.Lik.	-3201.810	-3166.962	-3141.436	-3136.862	-3122.635
Odds Ratios and 95% confidence intervals.					

Table 11 Step wise regression results for Germany, Outcome: Increased hand hygiene

(Intercept)	7.435	9.324	7.918	7.784	11.322
	[6.015, 9.301]	[7.516, 11.704]	[6.232, 10.162]	[6.126, 9.991]	[8.720, 14.840]
	s.e. = 0.826	s.e. = 1.052	s.e. = 0.987	s.e. = 0.971	s.e. = 1.535
	p = <0.001	p = <0.001	p = <0.001	p = <0.001	p = <0.001
Image: Couple blowing nose	1.176	1.276	1.293	1.279	1.591
	[0.757, 1.882]	[0.819, 2.048]	[0.829, 2.077]	[0.819, 2.054]	[1.016, 2.563]
	s.e. = 0.272	s.e. = 0.297	s.e. = 0.302	s.e. = 0.299	s.e. = 0.374
	p = 0.485	p = 0.295	p = 0.271	p = 0.293	p = 0.048
image5 - Woman wearing mask	0.999	1.102	1.203	1.184	1.162
	[0.795, 1.241]	[0.876, 1.371]	[0.953, 1.502]	[0.938, 1.479]	[0.918, 1.454]
	s.e. = 0.113	s.e. = 0.126	s.e. = 0.140	s.e. = 0.138	s.e. = 0.136
	p = 0.995	p = 0.395	p = 0.111	p = 0.145	p = 0.200
Image: Man wearing mask	0.998	1.106	1.113	1.103	1.081
	[0.780, 1.265]	[0.863, 1.405]	[0.867, 1.414]	[0.860, 1.402]	[0.841, 1.377]
	s.e. = 0.123	s.e. = 0.137	s.e. = 0.138	s.e. = 0.137	s.e. = 0.136
	p = 0.988	p = 0.416	p = 0.392	p = 0.431	p = 0.536
Sex: Male		0.501	0.487	0.485	0.493
		[0.462, 0.544]	[0.448, 0.529]	[0.446, 0.527]	[0.453, 0.536]
		s.e. = 0.021	s.e. = 0.021	s.e. = 0.021	s.e. = 0.021
		p = <0.001	p = <0.001	p = <0.001	p = <0.001
Age: 25- 44			1.055	1.011	1.071
			[0.937, 1.187]	[0.896, 1.139]	[0.949, 1.209]
			s.e. = 0.064	s.e. = 0.062	s.e. = 0.066
			p = 0.374	p = 0.860	p = 0.263
Age: 45- 64			1.082	1.052	1.162
			[0.956, 1.224]	[0.928, 1.191]	[1.023, 1.318]
			s.e. = 0.068	s.e. = 0.067	s.e. = 0.075
			p = 0.210	p = 0.426	p = 0.020
Age: 65+			1.607	1.549	1.677
			[1.369, 1.889]	[1.318, 1.823]	[1.425, 1.976]
			s.e. = 0.132	s.e. = 0.128	s.e. = 0.140
			p = <0.001	p = <0.001	p = <0.001
Education: University level				1.210	1.233

				[1.105, 1.327]	[1.125, 1.352]
				s.e. = 0.056	s.e. = 0.058
				p = <0.001	p = <0.001
Education: Postgraduate Degree				1.117	1.133
				[0.890, 1.417]	[0.902, 1.439]
				s.e. = 0.132	s.e. = 0.135
				p = 0.352	p = 0.294
Month: April					0.701
					[0.610, 0.803]
					s.e. = 0.049
					p = <0.001
Month: May					0.589
					[0.507, 0.682]
					s.e. = 0.044
					p = <0.001
Month: June					0.451
					[0.371, 0.549]
					s.e. = 0.045
					p = <0.001
Month: July					0.395
					[0.329, 0.475]
					s.e. = 0.037
					p = <0.001
Month: August					0.477
					[0.377, 0.607]
					s.e. = 0.058
					p = <0.001
Num.Obs.	22363	22363	22363	22363	22363
AIC	16280.2	16007.5	15970.4	15957.2	15831.0
Log.Lik.	-8136.100	-7998.739	-7977.185	-7968.613	-7900.504
Odds Ratios and 95% confidence intervals.					

Table 12 Step wise regression results for Italy, Outcome: Increased hand hygiene

(Intercept)	12.264	13.999	11.200	10.834	11.967
	[9.921, 15.368]	[11.273, 17.614]	[8.657, 14.650]	[8.363, 14.189]	[9.074, 15.939]
	s.e. = 1.367	s.e. = 1.592	s.e. = 1.502	s.e. = 1.460	s.e. = 1.719
	p = <0.001	p = <0.001	p = <0.001	p = <0.001	p = <0.001
Image: Couple blowing nose	0.599	0.607	0.613	0.604	0.596
	[0.402, 0.909]	[0.406, 0.921]	[0.410, 0.932]	[0.404, 0.919]	[0.397, 0.908]
	s.e. = 0.124	s.e. = 0.126	s.e. = 0.128	s.e. = 0.126	s.e. = 0.125
	p = 0.014	p = 0.016	p = 0.019	p = 0.016	p = 0.014
image5 - Woman wearing mask	0.956	0.993	1.017	1.008	0.992
	[0.756, 1.195]	[0.784, 1.242]	[0.801, 1.275]	[0.794, 1.265]	[0.778, 1.251]
	s.e. = 0.112	s.e. = 0.116	s.e. = 0.121	s.e. = 0.120	s.e. = 0.120
	p = 0.702	p = 0.949	p = 0.889	p = 0.945	p = 0.949
Image: Man wearing mask	0.793	0.802	0.793	0.788	0.803
	[0.598, 1.046]	[0.605, 1.059]	[0.597, 1.048]	[0.593, 1.041]	[0.604, 1.063]
	s.e. = 0.113	s.e. = 0.115	s.e. = 0.114	s.e. = 0.113	s.e. = 0.116
	p = 0.103	p = 0.123	p = 0.105	p = 0.096	p = 0.128
Sex: Male		0.646	0.641	0.646	0.640
		[0.572, 0.731]	[0.567, 0.725]	[0.571, 0.732]	[0.566, 0.725]
		s.e. = 0.041	s.e. = 0.040	s.e. = 0.041	s.e. = 0.041
		p = <0.001	p = <0.001	p = <0.001	p = <0.001
Age: 25- 44			1.298	1.229	1.247
			[1.092, 1.538]	[1.030, 1.464]	[1.043, 1.487]
			s.e. = 0.113	s.e. = 0.110	s.e. = 0.113
			p = 0.003	p = 0.021	p = 0.015
Age: 45- 64			1.237	1.196	1.201
			[1.034, 1.478]	[0.997, 1.433]	[0.999, 1.442]
			s.e. = 0.113	s.e. = 0.110	s.e. = 0.112
			p = 0.019	p = 0.052	p = 0.051
Age: 65+			1.380	1.326	1.336
			[1.105, 1.729]	[1.059, 1.665]	[1.065, 1.680]
			s.e. = 0.158	s.e. = 0.153	s.e. = 0.155
			p = 0.005	p = 0.014	p = 0.013
Education: University level				1.190	1.190
				[1.040, 1.364]	[1.039, 1.364]
				s.e. = 0.082	s.e. = 0.082

				p = 0.012	p = 0.012
Education: Postgraduate Degree				1.184	1.176
				[0.952, 1.487]	[0.945, 1.478]
				s.e. = 0.135	s.e. = 0.134
				p = 0.137	p = 0.154
Month: April					0.758
					[0.653, 0.880]
					s.e. = 0.058
					p = <0.001
Month: May					0.926
					[0.777, 1.108]
					s.e. = 0.084
					p = 0.396
Month: June					1.409
					[0.978, 2.101]
					s.e. = 0.274
					p = 0.078
Month: July					0.742
					[0.575, 0.970]
					s.e. = 0.099
					p = 0.025
Month: August					1.133
					[0.737, 1.837]
					s.e. = 0.263
					p = 0.591
Num.Obs.	14202	14202	14202	14202	14202
AIC	7969.2	7923.9	7919.0	7915.8	7902.2
Log.Lik.	-3980.608	-3956.942	-3951.503	-3947.893	-3936.101
Odds Ratios and 95% confidence intervals.					

Table 13 Step wise regression results for the Netherlands, Outcome: Increased hand hygiene

(Intercept)	8.614	12.290	11.296	11.349	13.865
	[7.331, 10.194]	[10.327, 14.728]	[8.608, 14.952]	[8.637, 15.042]	[10.489, 18.486]
	s.e. = 0.724	s.e. = 1.112	s.e. = 1.590	s.e. = 1.606	s.e. = 2.003
	p = <0.001	p = <0.001	p = <0.001	p = <0.001	p = <0.001
Image: Couple blowing nose	0.948	0.990	1.008	1.010	1.415
	[0.613, 1.527]	[0.636, 1.602]	[0.647, 1.635]	[0.648, 1.638]	[0.899, 2.313]
	s.e. = 0.220	s.e. = 0.232	s.e. = 0.238	s.e. = 0.238	s.e. = 0.340
	p = 0.818	p = 0.965	p = 0.971	p = 0.966	p = 0.149
image5 - Woman wearing mask	0.712	0.809	0.845	0.850	0.944
	[0.590, 0.853]	[0.669, 0.973]	[0.696, 1.023]	[0.698, 1.029]	[0.772, 1.149]
	s.e. = 0.067	s.e. = 0.077	s.e. = 0.083	s.e. = 0.084	s.e. = 0.096
	p = <0.001	p = 0.026	p = 0.087	p = 0.100	p = 0.568
Image: Man wearing mask	0.905	0.928	0.919	0.921	1.006
	[0.726, 1.126]	[0.743, 1.158]	[0.735, 1.148]	[0.737, 1.151]	[0.799, 1.265]
	s.e. = 0.101	s.e. = 0.105	s.e. = 0.104	s.e. = 0.105	s.e. = 0.118
	p = 0.373	p = 0.510	p = 0.459	p = 0.471	p = 0.959
Sex: Male		0.385	0.377	0.377	0.392
		[0.337, 0.440]	[0.329, 0.431]	[0.330, 0.432]	[0.342, 0.450]
		s.e. = 0.026	s.e. = 0.026	s.e. = 0.026	s.e. = 0.028
		p = <0.001	p = <0.001	p = <0.001	p = <0.001
Age: 25- 44			0.967	0.971	1.128
			[0.751, 1.237]	[0.754, 1.244]	[0.871, 1.454]
			s.e. = 0.123	s.e. = 0.124	s.e. = 0.147
			p = 0.790	p = 0.818	p = 0.357
Age: 45- 64			1.075	1.073	1.331
			[0.847, 1.355]	[0.845, 1.353]	[1.043, 1.688]
			s.e. = 0.129	s.e. = 0.129	s.e. = 0.163
			p = 0.544	p = 0.556	p = 0.020
Age: 65+			1.193	1.190	1.483
			[0.924, 1.530]	[0.922, 1.528]	[1.144, 1.913]
			s.e. = 0.153	s.e. = 0.153	s.e. = 0.194
			p = 0.170	p = 0.177	p = 0.003
Education: University level				0.971	1.083
				[0.832, 1.136]	[0.924, 1.271]
				s.e. = 0.077	s.e. = 0.088

				p = 0.709	p = 0.330
Education: Postgraduate Degree				0.957	1.127
				[0.611, 1.569]	[0.712, 1.864]
				s.e. = 0.229	s.e. = 0.276
				p = 0.855	p = 0.624
Month: May					0.504
					[0.427, 0.597]
					s.e. = 0.043
					p = <0.001
Month: June					0.284
					[0.226, 0.358]
					s.e. = 0.033
					p = <0.001
Month: July					0.276
					[0.225, 0.339]
					s.e. = 0.029
					p = <0.001
Month: August					0.254
					[0.189, 0.345]
					s.e. = 0.039
					p = <0.001
Num.Obs.	8223	8223	8223	8223	8223
AIC	6244.5	6046.6	6047.9	6051.8	5813.8
Log.Lik.	-3118.237	-3018.298	-3015.960	-3015.880	-2892.917
Odds Ratios and 95% confidence intervals.					

Table 14 Step wise regression results for Spain, Outcome: Increased hand hygiene

(Intercept)	15.186	18.499	10.943	10.447	11.576
	[12.021, 19.514]	[14.491, 24.006]	[7.482, 16.370]	[7.037, 15.846]	[7.485, 18.279]
	s.e. = 1.874	s.e. = 2.379	s.e. = 2.183	s.e. = 2.161	s.e. = 2.635
	p = <0.001	p = <0.001	p = <0.001	p = <0.001	p = <0.001
Image: Couple blowing nose	1.028	1.069	1.097	1.095	1.135
	[0.616, 1.804]	[0.639, 1.879]	[0.655, 1.931]	[0.654, 1.926]	[0.675, 2.002]
	s.e. = 0.280	s.e. = 0.292	s.e. = 0.301	s.e. = 0.300	s.e. = 0.313
	p = 0.919	p = 0.807	p = 0.735	p = 0.741	p = 0.647
image5 - Woman wearing mask	1.372	1.412	1.467	1.460	1.482
	[1.044, 1.779]	[1.073, 1.833]	[1.111, 1.913]	[1.105, 1.905]	[1.119, 1.939]
	s.e. = 0.186	s.e. = 0.192	s.e. = 0.203	s.e. = 0.203	s.e. = 0.207
	p = 0.020	p = 0.011	p = 0.006	p = 0.006	p = 0.005
Image: Man wearing mask	1.318	1.358	1.341	1.338	1.364
	[0.968, 1.782]	[0.997, 1.838]	[0.984, 1.815]	[0.982, 1.812]	[0.999, 1.849]
	s.e. = 0.205	s.e. = 0.212	s.e. = 0.209	s.e. = 0.209	s.e. = 0.214
	p = 0.075	p = 0.049	p = 0.060	p = 0.062	p = 0.048
Sex: Male		0.552	0.534	0.538	0.538
		[0.463, 0.659]	[0.446, 0.640]	[0.449, 0.645]	[0.449, 0.645]
		s.e. = 0.050	s.e. = 0.049	s.e. = 0.050	s.e. = 0.050
		p = <0.001	p = <0.001	p = <0.001	p = <0.001
Age: 25- 44			1.720	1.733	1.788
			[1.208, 2.402]	[1.217, 2.422]	[1.254, 2.503]
			s.e. = 0.301	s.e. = 0.304	s.e. = 0.315
			p = 0.002	p = 0.002	p = <0.001
Age: 45- 64			1.661	1.678	1.739
			[1.176, 2.299]	[1.187, 2.323]	[1.228, 2.413]
			s.e. = 0.283	s.e. = 0.287	s.e. = 0.299
			p = 0.003	p = 0.002	p = 0.001
Age: 65+			2.044	2.066	2.137
			[1.377, 3.004]	[1.390, 3.040]	[1.435, 3.151]
			s.e. = 0.406	s.e. = 0.412	s.e. = 0.428
			p = <0.001	p = <0.001	p = <0.001
Education: University level				1.093	1.092
				[0.902, 1.321]	[0.901, 1.320]
				s.e. = 0.106	s.e. = 0.106
				p = 0.361	p = 0.367
Education: Postgraduate Degree				0.907	0.914
				[0.673, 1.240]	[0.678, 1.249]
				s.e. = 0.141	s.e. = 0.142
				p = 0.532	p = 0.561
Month: April					0.801

					[0.616, 1.032]
					s.e. = 0.105
					p = 0.092
Month: May					1.051
					[0.765, 1.441]
					s.e. = 0.169
					p = 0.759
Month: June					0.838
					[0.545, 1.321]
					s.e. = 0.189
					p = 0.432
Month: July					0.696
					[0.468, 1.050]
					s.e. = 0.143
					p = 0.077
Month: August					0.655
					[0.386, 1.183]
					s.e. = 0.186
					p = 0.137
Num.Obs.	10964	10964	10964	10964	10964
AIC	4241.3	4201.1	4194.7	4196.8	4197.7
Log.Lik.	-2116.635	-2095.565	-2089.329	-2088.383	-2083.849
Odds Ratios and 95% confidence intervals.					

Table 15 Step wise regression results for the United Kingdom, Outcome: Increased hand hygiene

(Intercept)	19.372	22.957	18.717	18.276	20.327
	[14.450, 26.716]	[17.007, 31.851]	[12.297, 29.235]	[11.948, 28.672]	[13.109, 32.295]
	s.e. = 3.029	s.e. = 3.666	s.e. = 4.130	s.e. = 4.076	s.e. = 4.670
	p = <0.001	p = <0.001	p = <0.001	p = <0.001	p = <0.001
Image: Couple blowing nose	0.444	0.455	0.463	0.456	0.467
	[0.270, 0.741]	[0.276, 0.760]	[0.281, 0.775]	[0.277, 0.764]	[0.283, 0.784]
	s.e. = 0.114	s.e. = 0.117	s.e. = 0.119	s.e. = 0.118	s.e. = 0.121
	p = 0.002	p = 0.002	p = 0.003	p = 0.002	p = 0.003
image5 - Woman wearing mask	0.911	0.947	0.976	0.948	0.922
	[0.652, 1.240]	[0.678, 1.291]	[0.697, 1.333]	[0.676, 1.296]	[0.656, 1.264]
	s.e. = 0.149	s.e. = 0.155	s.e. = 0.161	s.e. = 0.157	s.e. = 0.154
	p = 0.567	p = 0.741	p = 0.883	p = 0.746	p = 0.627
Image: Man wearing mask	0.709	0.750	0.745	0.725	0.738
	[0.489, 1.011]	[0.517, 1.071]	[0.513, 1.063]	[0.499, 1.036]	[0.508, 1.055]
	s.e. = 0.131	s.e. = 0.139	s.e. = 0.138	s.e. = 0.135	s.e. = 0.137
	p = 0.063	p = 0.121	p = 0.112	p = 0.084	p = 0.103
Sex: Male		0.598	0.593	0.590	0.587
		[0.510, 0.702]	[0.505, 0.697]	[0.502, 0.693]	[0.499, 0.690]
		s.e. = 0.049	s.e. = 0.049	s.e. = 0.048	s.e. = 0.048
		p = <0.001	p = <0.001	p = <0.001	p = <0.001
Age: 25- 44			1.184	1.128	1.145
			[0.834, 1.653]	[0.794, 1.578]	[0.805, 1.602]
			s.e. = 0.206	s.e. = 0.197	s.e. = 0.201
			p = 0.333	p = 0.491	p = 0.441
Age: 45- 64			1.165	1.143	1.177
			[0.838, 1.587]	[0.822, 1.557]	[0.845, 1.607]
			s.e. = 0.189	s.e. = 0.186	s.e. = 0.193
			p = 0.348	p = 0.413	p = 0.318
Age: 65+			1.344	1.333	1.380
			[0.953, 1.864]	[0.944, 1.849]	[0.976, 1.918]
			s.e. = 0.230	s.e. = 0.228	s.e. = 0.237
			p = 0.083	p = 0.093	p = 0.061
Education: University level				1.091	1.092
				[0.924, 1.290]	[0.924, 1.291]
				s.e. = 0.093	s.e. = 0.093
				p = 0.305	p = 0.303
Education: Postgraduate Degree				1.650	1.655
				[1.163, 2.417]	[1.166, 2.425]
				s.e. = 0.307	s.e. = 0.308
				p = 0.007	p = 0.007
Month: April					0.805

					[0.656, 0.991]
					s.e. = 0.085
					p = 0.039
Month: May					0.844
					[0.674, 1.063]
					s.e. = 0.098
					p = 0.146
Month: June					1.057
					[0.716, 1.615]
					s.e. = 0.218
					p = 0.790
Month: July					0.771
					[0.565, 1.071]
					s.e. = 0.126
					p = 0.110
Month: August					0.654
					[0.407, 1.115]
					s.e. = 0.167
					p = 0.096
Num.Obs.	11401	11401	11401	11401	11401
AIC	4945.3	4908.4	4910.7	4906.5	4908.4
Log.Lik.	-2468.666	-2449.216	-2447.352	-2443.242	-2439.195
Odds Ratios and 95% confidence intervals.					

Table 16 Step wise regression results for the United States, Outcome: Increased hand hygiene

(Intercept)	11.727	16.568	17.193	15.004	16.391
	[9.199, 15.219]	[12.922, 21.611]	[12.781, 23.470]	[11.128, 20.523]	[12.016, 22.664]
	s.e. = 1.504	s.e. = 2.170	s.e. = 2.663	s.e. = 2.340	s.e. = 2.651
	p = <0.001	p = <0.001	p = <0.001	p = <0.001	p = <0.001
Image: Couple blowing nose	1.076	1.111	1.104	1.090	1.091
	[0.690, 1.717]	[0.710, 1.777]	[0.705, 1.768]	[0.696, 1.747]	[0.696, 1.748]
	s.e. = 0.249	s.e. = 0.259	s.e. = 0.258	s.e. = 0.255	s.e. = 0.255
	p = 0.751	p = 0.653	p = 0.672	p = 0.712	p = 0.711
image5 - Woman wearing mask	1.058	1.121	1.133	1.128	1.102
	[0.812, 1.356]	[0.859, 1.440]	[0.867, 1.456]	[0.863, 1.450]	[0.841, 1.422]
	s.e. = 0.138	s.e. = 0.148	s.e. = 0.149	s.e. = 0.149	s.e. = 0.147
	p = 0.663	p = 0.385	p = 0.343	p = 0.361	p = 0.466
Image: Man wearing mask	0.987	1.051	1.041	1.031	1.027
	[0.741, 1.296]	[0.788, 1.384]	[0.780, 1.371]	[0.773, 1.359]	[0.767, 1.355]
	s.e. = 0.141	s.e. = 0.151	s.e. = 0.149	s.e. = 0.148	s.e. = 0.149
	p = 0.926	p = 0.727	p = 0.779	p = 0.829	p = 0.857
Sex: Male		0.408	0.407	0.417	0.416
		[0.373, 0.446]	[0.372, 0.446]	[0.381, 0.456]	[0.380, 0.455]
		s.e. = 0.019	s.e. = 0.019	s.e. = 0.019	s.e. = 0.019
		p = <0.001	p = <0.001	p = <0.001	p = <0.001
Age: 25- 44			0.973	0.880	0.894
			[0.803, 1.174]	[0.725, 1.062]	[0.736, 1.081]
			s.e. = 0.094	s.e. = 0.086	s.e. = 0.088
			p = 0.780	p = 0.189	p = 0.255
Age: 45- 64			0.872	0.811	0.829
			[0.727, 1.040]	[0.675, 0.968]	[0.689, 0.991]
			s.e. = 0.080	s.e. = 0.075	s.e. = 0.077
			p = 0.134	p = 0.023	p = 0.043
Age: 65+			1.047	0.954	0.977
			[0.869, 1.255]	[0.791, 1.146]	[0.808, 1.174]
			s.e. = 0.098	s.e. = 0.090	s.e. = 0.093
			p = 0.622	p = 0.621	p = 0.805
Education: University level				1.427	1.426
				[1.301, 1.564]	[1.301, 1.564]
				s.e. = 0.067	s.e. = 0.067
				p = <0.001	p = <0.001
Education: Postgraduate Degree				1.654	1.647
				[1.330, 2.082]	[1.324, 2.072]
				s.e. = 0.189	s.e. = 0.188
				p = <0.001	p = <0.001
Month: April					0.873

					[0.772, 0.986]
					s.e. = 0.054
					p = 0.029
Month: May					0.939
					[0.825, 1.070]
					s.e. = 0.062
					p = 0.346
Month: June					0.878
					[0.700, 1.110]
					s.e. = 0.103
					p = 0.266
Month: July					0.921
					[0.778, 1.095]
					s.e. = 0.080
					p = 0.348
Month: August					0.693
					[0.536, 0.906]
					s.e. = 0.092
					p = 0.006
Num.Obs.	28400	28400	28400	28400	28400
AIC	15177.6	14789.7	14783.8	14722.5	14722.4
Log.Lik.	-7584.781	-7389.841	-7383.915	-7351.239	-7346.210
Odds Ratios and 95% confidence intervals.					

Table 17 Step wise regression results for Belgium, Outcome: Threat perception of COVID-19 to the family

	response					
(Intercept)	Low	0.759	0.657	0.837	0.836	0.607
		[0.644, 0.895]	[0.554, 0.778]	[0.674, 1.039]	[0.672, 1.040]	[0.484, 0.760]
		s.e. = 0.064	s.e. = 0.057	s.e. = 0.092	s.e. = 0.093	s.e. = 0.070
		p = 0.001	p = <0.001	p = 0.107	p = 0.108	p = <0.001
	Medium	0.915	0.900	1.076	1.027	0.899
		[0.782, 1.070]	[0.768, 1.056]	[0.880, 1.316]	[0.839, 1.258]	[0.731, 1.105]
		s.e. = 0.073	s.e. = 0.073	s.e. = 0.110	s.e. = 0.106	s.e. = 0.095
		p = 0.264	p = 0.195	p = 0.474	p = 0.797	p = 0.311
Image: Couple blowing nose	Low	1.106	1.108	1.050	1.047	0.855
		[0.726, 1.685]	[0.726, 1.690]	[0.687, 1.605]	[0.685, 1.599]	[0.557, 1.315]
		s.e. = 0.238	s.e. = 0.239	s.e. = 0.227	s.e. = 0.226	s.e. = 0.188
		p = 0.640	p = 0.635	p = 0.821	p = 0.833	p = 0.476
	Medium	1.523	1.523	1.449	1.431	1.300
		[1.044, 2.221]	[1.044, 2.221]	[0.992, 2.116]	[0.979, 2.091]	[0.888, 1.904]
		s.e. = 0.293	s.e. = 0.293	s.e. = 0.280	s.e. = 0.277	s.e. = 0.253
		p = 0.029	p = 0.029	p = 0.055	p = 0.064	p = 0.177
image5 - Woman wearing mask	Low	0.995	0.983	0.948	0.941	1.030
		[0.834, 1.185]	[0.825, 1.173]	[0.791, 1.135]	[0.785, 1.129]	[0.856, 1.239]
		s.e. = 0.089	s.e. = 0.088	s.e. = 0.087	s.e. = 0.087	s.e. = 0.097
		p = 0.951	p = 0.852	p = 0.560	p = 0.514	p = 0.754
	Medium	1.225	1.223	1.216	1.166	1.224
		[1.038, 1.446]	[1.036, 1.444]	[1.026, 1.440]	[0.983, 1.383]	[1.030, 1.455]
		s.e. = 0.104	s.e. = 0.104	s.e. = 0.105	s.e. = 0.102	s.e. = 0.108
		p = 0.017	p = 0.017	p = 0.024	p = 0.078	p = 0.022
Image: Man wearing mask	Low	0.813	0.834	0.835	0.835	0.964
		[0.673, 0.983]	[0.690, 1.009]	[0.691, 1.010]	[0.690, 1.009]	[0.794, 1.170]
		s.e. = 0.078	s.e. = 0.081	s.e. = 0.081	s.e. = 0.081	s.e. = 0.095
		p = 0.032	p = 0.061	p = 0.063	p = 0.062	p = 0.709
	Medium	1.131	1.134	1.135	1.120	1.194
		[0.948, 1.348]	[0.951, 1.352]	[0.951, 1.353]	[0.939, 1.336]	[0.999, 1.426]
		s.e. = 0.102	s.e. = 0.102	s.e. = 0.102	s.e. = 0.101	s.e. = 0.108
		p = 0.171	p = 0.161	p = 0.160	p = 0.208	p = 0.051
Sex: Male	Low		1.499	1.530	1.528	1.493
			[1.355, 1.658]	[1.381, 1.694]	[1.379, 1.693]	[1.345, 1.656]
			s.e. = 0.077	s.e. = 0.080	s.e. = 0.080	s.e. = 0.079
			p = <0.001	p = <0.001	p = <0.001	p = <0.001
	Medium		1.052	1.058	1.069	1.061
			[0.958, 1.154]	[0.963, 1.162]	[0.972, 1.174]	[0.965, 1.167]
			s.e. = 0.050	s.e. = 0.051	s.e. = 0.051	s.e. = 0.051
			p = 0.288	p = 0.240	p = 0.167	p = 0.218
Age: 25- 44	Low			0.777	0.770	0.711

	response					
				[0.662, 0.911]	[0.655, 0.904]	[0.604, 0.837]
				s.e. = 0.063	s.e. = 0.063	s.e. = 0.059
				p = 0.002	p = 0.001	p = <0.001
	Medium			0.804	0.773	0.747
				[0.696, 0.928]	[0.669, 0.894]	[0.646, 0.864]
				s.e. = 0.059	s.e. = 0.057	s.e. = 0.055
				p = 0.003	p = <0.001	p = <0.001
Age: 45- 64	Low			0.776	0.772	0.672
				[0.663, 0.908]	[0.659, 0.904]	[0.572, 0.790]
				s.e. = 0.062	s.e. = 0.062	s.e. = 0.055
				p = 0.002	p = 0.001	p = <0.001
	Medium			0.779	0.765	0.722
				[0.675, 0.898]	[0.663, 0.883]	[0.625, 0.834]
				s.e. = 0.057	s.e. = 0.056	s.e. = 0.053
				p = <0.001	p = <0.001	p = <0.001
Age: 65+	Low			0.750	0.744	0.636
				[0.627, 0.896]	[0.622, 0.890]	[0.530, 0.763]
				s.e. = 0.068	s.e. = 0.068	s.e. = 0.059
				p = 0.002	p = 0.001	p = <0.001
	Medium			0.906	0.883	0.822
				[0.773, 1.061]	[0.753, 1.035]	[0.700, 0.966]
				s.e. = 0.073	s.e. = 0.072	s.e. = 0.068
				p = 0.220	p = 0.126	p = 0.017
Education: University level	Low				1.015	0.962
					[0.918, 1.122]	[0.869, 1.065]
					s.e. = 0.052	s.e. = 0.050
					p = 0.777	p = 0.457
	Medium				1.204	1.178
					[1.101, 1.317]	[1.076, 1.289]
					s.e. = 0.055	s.e. = 0.054
					p = <0.001	p = <0.001
Education: Postgraduate Degree	Low				1.185	1.153
					[0.888, 1.582]	[0.860, 1.547]
					s.e. = 0.175	s.e. = 0.173
					p = 0.249	p = 0.342
	Medium				1.198	1.183
					[0.917, 1.564]	[0.905, 1.546]
					s.e. = 0.163	s.e. = 0.162
					p = 0.185	p = 0.218
Month: May	Low					2.040
						[1.802, 2.310]
						s.e. = 0.129
						p = <0.001
	Medium					1.344
						[1.200, 1.506]
						s.e. = 0.078
						p = <0.001
Month: June	Low					2.279
						[1.851, 2.806]
						s.e. = 0.242
						p = <0.001
	Medium					1.649
						[1.362, 1.995]
						s.e. = 0.160
						p = <0.001
Month: July	Low					2.476
						[2.106, 2.911]
						s.e. = 0.205
						p = <0.001
	Medium					1.599
						[1.375, 1.861]
						s.e. = 0.124
						p = <0.001
Month: August	Low					4.069
						[3.159, 5.242]
						s.e. = 0.526
						p = <0.001
	Medium					1.588
						[1.220, 2.067]
						s.e. = 0.213
						p = <0.001

	response					
Num.Obs.		11144	11144	11144	11144	11144
AIC		24151.3	24084.2	24071.3	24058.3	23759.3
Odds Ratios and 95% confidence intervals.						

Table 18 Step wise regression results for France, Outcome: Threat perception of COVID-19 to the family

	response					
(Intercept)	Low	0.872	0.760	0.965	0.931	0.546
		[0.685, 1.111]	[0.595, 0.971]	[0.733, 1.271]	[0.697, 1.244]	[0.399, 0.745]
		s.e. = 0.108	s.e. = 0.095	s.e. = 0.135	s.e. = 0.138	s.e. = 0.087
		p = 0.268	p = 0.028	p = 0.801	p = 0.628	p = <0.001
	Medium	1.156	1.191	1.517	1.369	1.178
		[0.923, 1.448]	[0.949, 1.494]	[1.177, 1.956]	[1.048, 1.788]	[0.892, 1.557]
		s.e. = 0.133	s.e. = 0.138	s.e. = 0.196	s.e. = 0.187	s.e. = 0.168
		p = 0.207	p = 0.132	p = 0.001	p = 0.021	p = 0.249
Image: Couple blowing nose	Low	1.341	1.448	1.310	1.299	0.897
		[0.864, 2.081]	[0.932, 2.250]	[0.841, 2.043]	[0.833, 2.026]	[0.570, 1.410]
		s.e. = 0.301	s.e. = 0.326	s.e. = 0.297	s.e. = 0.295	s.e. = 0.207
		p = 0.190	p = 0.099	p = 0.233	p = 0.248	p = 0.636
	Medium	1.208	1.187	1.066	1.049	0.934
		[0.795, 1.836]	[0.781, 1.805]	[0.699, 1.627]	[0.687, 1.600]	[0.609, 1.432]
		s.e. = 0.258	s.e. = 0.254	s.e. = 0.230	s.e. = 0.226	s.e. = 0.204
		p = 0.377	p = 0.422	p = 0.765	p = 0.826	p = 0.754
image5 - Woman wearing mask	Low	0.875	0.875	0.842	0.836	0.903
		[0.684, 1.121]	[0.683, 1.122]	[0.656, 1.080]	[0.651, 1.074]	[0.699, 1.166]
		s.e. = 0.110	s.e. = 0.111	s.e. = 0.107	s.e. = 0.107	s.e. = 0.118
		p = 0.292	p = 0.293	p = 0.176	p = 0.161	p = 0.433
	Medium	0.988	0.988	0.945	0.928	0.956
		[0.785, 1.244]	[0.785, 1.244]	[0.750, 1.191]	[0.735, 1.170]	[0.756, 1.208]
		s.e. = 0.116	s.e. = 0.116	s.e. = 0.112	s.e. = 0.110	s.e. = 0.114
		p = 0.918	p = 0.918	p = 0.631	p = 0.526	p = 0.704
Image: Man wearing mask	Low	0.913	0.933	0.912	0.908	0.914
		[0.693, 1.203]	[0.707, 1.230]	[0.691, 1.205]	[0.688, 1.200]	[0.689, 1.214]
		s.e. = 0.129	s.e. = 0.132	s.e. = 0.129	s.e. = 0.129	s.e. = 0.132
		p = 0.517	p = 0.623	p = 0.518	p = 0.499	p = 0.536
	Medium	1.062	1.057	1.025	1.015	1.006
		[0.823, 1.371]	[0.819, 1.364]	[0.794, 1.325]	[0.785, 1.311]	[0.777, 1.302]
		s.e. = 0.138	s.e. = 0.138	s.e. = 0.134	s.e. = 0.133	s.e. = 0.132
		p = 0.642	p = 0.670	p = 0.847	p = 0.911	p = 0.963
Sex: Male	Low		1.504	1.517	1.525	1.486
			[1.361, 1.664]	[1.372, 1.678]	[1.378, 1.687]	[1.341, 1.648]
			s.e. = 0.077	s.e. = 0.078	s.e. = 0.079	s.e. = 0.078
			p = <0.001	p = <0.001	p = <0.001	p = <0.001
	Medium		0.901	0.909	0.911	0.911
			[0.819, 0.991]	[0.826, 1.000]	[0.828, 1.003]	[0.827, 1.003]
			s.e. = 0.044	s.e. = 0.044	s.e. = 0.045	s.e. = 0.045
			p = 0.032	p = 0.050	p = 0.057	p = 0.057
Age: 25- 44	Low			0.797	0.804	0.752
				[0.689, 0.923]	[0.694, 0.930]	[0.649, 0.873]
				s.e. = 0.059	s.e. = 0.060	s.e. = 0.057
				p = 0.002	p = 0.003	p = <0.001
	Medium			0.817	0.819	0.809
				[0.716, 0.931]	[0.718, 0.935]	[0.709, 0.924]
				s.e. = 0.055	s.e. = 0.055	s.e. = 0.055
				p = 0.003	p = 0.003	p = 0.002
Age: 45- 64	Low			0.764	0.776	0.705
				[0.662, 0.881]	[0.671, 0.899]	[0.607, 0.819]
				s.e. = 0.056	s.e. = 0.058	s.e. = 0.054
				p = <0.001	p = <0.001	p = <0.001
	Medium			0.738	0.756	0.744
				[0.649, 0.841]	[0.662, 0.864]	[0.651, 0.850]
				s.e. = 0.049	s.e. = 0.051	s.e. = 0.051
				p = <0.001	p = <0.001	p = <0.001
Age: 65+	Low			0.796	0.812	0.720
				[0.676, 0.938]	[0.687, 0.960]	[0.607, 0.854]
				s.e. = 0.066	s.e. = 0.069	s.e. = 0.063

	response					
				p = 0.006	p = 0.015	p = <0.001
	Medium			0.822	0.845	0.828
				[0.709, 0.953]	[0.727, 0.983]	[0.711, 0.964]
				s.e. = 0.062	s.e. = 0.065	s.e. = 0.064
				p = 0.009	p = 0.029	p = 0.015
Education: University level	Low				1.048	1.013
					[0.932, 1.178]	[0.900, 1.141]
					s.e. = 0.063	s.e. = 0.061
					p = 0.434	p = 0.831
	Medium				1.139	1.131
					[1.024, 1.268]	[1.016, 1.259]
					s.e. = 0.062	s.e. = 0.062
					p = 0.017	p = 0.024
Education: Postgraduate Degree	Low				0.940	0.855
					[0.760, 1.163]	[0.689, 1.061]
					s.e. = 0.102	s.e. = 0.094
					p = 0.569	p = 0.155
	Medium				1.203	1.179
					[0.996, 1.452]	[0.976, 1.424]
					s.e. = 0.116	s.e. = 0.114
					p = 0.055	p = 0.087
Month: April	Low					1.428
						[1.238, 1.648]
						s.e. = 0.104
						p = <0.001
	Medium					1.109
						[0.990, 1.244]
						s.e. = 0.065
						p = 0.075
Month: May	Low					2.449
						[2.089, 2.870]
						s.e. = 0.199
						p = <0.001
	Medium					1.305
						[1.139, 1.494]
						s.e. = 0.090
						p = <0.001
Month: June	Low					3.260
						[2.587, 4.109]
						s.e. = 0.385
						p = <0.001
	Medium					1.545
						[1.248, 1.911]
						s.e. = 0.168
						p = <0.001
Month: July	Low					2.669
						[2.221, 3.206]
						s.e. = 0.250
						p = <0.001
	Medium					1.325
						[1.124, 1.561]
						s.e. = 0.111
						p = <0.001
Month: August	Low					3.999
						[3.145, 5.086]
						s.e. = 0.490
						p = <0.001
	Medium					1.097
						[0.856, 1.406]
						s.e. = 0.139
						p = 0.465
Num.Obs.		11670	11670	11670	11670	11670
AIC		25348.8	25241.4	25228.4	25226.8	24928.4
Odds Ratios and 95% confidence intervals.						

Table 19 Step wise regression results for Germany, Outcome: Threat perception of COVID-19 to the family

	response					
(Intercept)	Low	1.440	1.273	1.012	1.023	0.529
		[1.212, 1.712]	[1.070, 1.516]	[0.833, 1.230]	[0.841, 1.243]	[0.429, 0.653]
		s.e. = 0.127	s.e. = 0.113	s.e. = 0.101	s.e. = 0.102	s.e. = 0.057
		p = <0.001	p = 0.007	p = 0.902	p = 0.822	p = <0.001
	Medium	1.119	1.113	1.274	1.266	1.028
		[0.932, 1.344]	[0.926, 1.337]	[1.041, 1.560]	[1.034, 1.550]	[0.833, 1.269]
		s.e. = 0.104	s.e. = 0.104	s.e. = 0.132	s.e. = 0.131	s.e. = 0.111
		p = 0.227	p = 0.253	p = 0.019	p = 0.023	p = 0.796
Image: Couple blowing nose	Low	1.308	1.248	1.326	1.334	0.894
		[0.914, 1.870]	[0.871, 1.786]	[0.925, 1.900]	[0.931, 1.911]	[0.621, 1.289]
		s.e. = 0.239	s.e. = 0.228	s.e. = 0.243	s.e. = 0.245	s.e. = 0.167
		p = 0.142	p = 0.227	p = 0.124	p = 0.117	p = 0.550
	Medium	1.340	1.337	1.301	1.296	1.139
		[0.922, 1.949]	[0.920, 1.944]	[0.894, 1.893]	[0.891, 1.886]	[0.780, 1.661]
		s.e. = 0.256	s.e. = 0.255	s.e. = 0.249	s.e. = 0.248	s.e. = 0.219
		p = 0.125	p = 0.128	p = 0.169	p = 0.175	p = 0.501
image5 - Woman wearing mask	Low	1.043	0.983	1.036	1.046	1.023
		[0.874, 1.245]	[0.823, 1.174]	[0.865, 1.240]	[0.873, 1.253]	[0.850, 1.231]
		s.e. = 0.094	s.e. = 0.089	s.e. = 0.095	s.e. = 0.096	s.e. = 0.097
		p = 0.638	p = 0.851	p = 0.704	p = 0.625	p = 0.812
	Medium	1.150	1.147	1.111	1.104	1.069
		[0.954, 1.386]	[0.951, 1.382]	[0.918, 1.343]	[0.913, 1.335]	[0.883, 1.295]
		s.e. = 0.109	s.e. = 0.109	s.e. = 0.108	s.e. = 0.107	s.e. = 0.104
		p = 0.142	p = 0.151	p = 0.279	p = 0.309	p = 0.491
Image: Man wearing mask	Low	1.047	0.984	1.012	1.016	0.994
		[0.864, 1.269]	[0.812, 1.194]	[0.834, 1.227]	[0.838, 1.233]	[0.815, 1.212]
		s.e. = 0.103	s.e. = 0.097	s.e. = 0.100	s.e. = 0.100	s.e. = 0.101
		p = 0.639	p = 0.871	p = 0.908	p = 0.869	p = 0.953
	Medium	1.047	1.044	1.033	1.030	0.992
		[0.855, 1.283]	[0.852, 1.279]	[0.843, 1.267]	[0.840, 1.262]	[0.808, 1.217]
		s.e. = 0.108	s.e. = 0.108	s.e. = 0.107	s.e. = 0.107	s.e. = 0.104
		p = 0.657	p = 0.679	p = 0.751	p = 0.775	p = 0.936
Sex: Male	Low		1.555	1.515	1.520	1.490
			[1.454, 1.664]	[1.415, 1.623]	[1.419, 1.628]	[1.390, 1.598]
			s.e. = 0.054	s.e. = 0.053	s.e. = 0.053	s.e. = 0.053
			p = <0.001	p = <0.001	p = <0.001	p = <0.001
	Medium		1.024	1.041	1.040	1.038
			[0.954, 1.100]	[0.968, 1.119]	[0.968, 1.118]	[0.965, 1.115]
			s.e. = 0.037	s.e. = 0.038	s.e. = 0.038	s.e. = 0.038
			p = 0.509	p = 0.277	p = 0.283	p = 0.316
Age: 25- 44	Low			1.208	1.242	1.140
				[1.097, 1.332]	[1.126, 1.371]	[1.031, 1.260]
				s.e. = 0.060	s.e. = 0.063	s.e. = 0.058
				p = <0.001	p = <0.001	p = 0.011
	Medium			0.874	0.863	0.843
				[0.795, 0.962]	[0.783, 0.951]	[0.764, 0.929]
				s.e. = 0.043	s.e. = 0.043	s.e. = 0.042
				p = 0.006	p = 0.003	p = <0.001
Age: 45- 64	Low			1.398	1.424	1.236
				[1.262, 1.549]	[1.285, 1.579]	[1.112, 1.373]
				s.e. = 0.073	s.e. = 0.075	s.e. = 0.066
				p = <0.001	p = <0.001	p = <0.001
	Medium			0.866	0.859	0.835
				[0.782, 0.960]	[0.775, 0.953]	[0.752, 0.927]
				s.e. = 0.045	s.e. = 0.045	s.e. = 0.044
				p = 0.006	p = 0.004	p = <0.001
Age: 65+	Low			1.121	1.148	1.033
				[0.991, 1.268]	[1.014, 1.299]	[0.910, 1.172]
				s.e. = 0.070	s.e. = 0.073	s.e. = 0.067
				p = 0.069	p = 0.029	p = 0.615
	Medium			0.855	0.846	0.831
				[0.756, 0.967]	[0.747, 0.959]	[0.733, 0.942]
				s.e. = 0.054	s.e. = 0.054	s.e. = 0.053
				p = 0.013	p = 0.009	p = 0.004
Education: University level	Low				0.892	0.864
					[0.830, 0.959]	[0.802, 0.930]

	response					
					s.e. = 0.033	s.e. = 0.032
					p = 0.002	p = <0.001
	Medium				1.069	1.061
					[0.992, 1.151]	[0.984, 1.142]
					s.e. = 0.040	s.e. = 0.040
					p = 0.080	p = 0.122
Education: Postgraduate Degree	Low				0.898	0.884
					[0.746, 1.082]	[0.731, 1.069]
					s.e. = 0.085	s.e. = 0.085
					p = 0.259	p = 0.203
	Medium				0.970	0.970
					[0.797, 1.181]	[0.796, 1.180]
					s.e. = 0.097	s.e. = 0.097
					p = 0.764	p = 0.758
Month: April	Low					1.980
						[1.795, 2.185]
						s.e. = 0.099
						p = <0.001
	Medium					1.340
						[1.223, 1.468]
						s.e. = 0.062
						p = <0.001
Month: May	Low					3.114
						[2.786, 3.481]
						s.e. = 0.177
						p = <0.001
	Medium					1.430
						[1.283, 1.595]
						s.e. = 0.080
						p = <0.001
Month: June	Low					4.469
						[3.759, 5.314]
						s.e. = 0.395
						p = <0.001
	Medium					1.537
						[1.276, 1.850]
						s.e. = 0.146
						p = <0.001
Month: July	Low					4.264
						[3.620, 5.023]
						s.e. = 0.356
						p = <0.001
	Medium					1.561
						[1.312, 1.857]
						s.e. = 0.138
						p = <0.001
Month: August	Low					3.695
						[2.989, 4.569]
						s.e. = 0.400
						p = <0.001
	Medium					1.502
						[1.196, 1.885]
						s.e. = 0.174
						p = <0.001
Num.Obs.		22363	22363	22363	22363	22363
AIC		48534.1	48300.9	48202.4	48181.6	47445.1
Odds Ratios and 95% confidence intervals.						

Table 20 Step wise regression results for Italy, Outcome: Threat perception of COVID-19 to the family

	response					
(Intercept)	Low	0.376	0.334	0.317	0.318	0.164
		[0.321, 0.440]	[0.284, 0.393]	[0.258, 0.389]	[0.259, 0.391]	[0.131, 0.206]
		s.e. = 0.030	s.e. = 0.028	s.e. = 0.033	s.e. = 0.034	s.e. = 0.019
		p = <0.001	p = <0.001	p = <0.001	p = <0.001	p = <0.001
	Medium	0.689	0.670	0.894	0.880	0.692
		[0.605, 0.784]	[0.587, 0.764]	[0.763, 1.047]	[0.750, 1.031]	[0.584, 0.819]
		s.e. = 0.046	s.e. = 0.045	s.e. = 0.072	s.e. = 0.071	s.e. = 0.060

	response					
		p = <0.001	p = <0.001	p = 0.165	p = 0.114	p = <0.001
Image: Couple blowing nose	Low	1.093	1.079	1.124	1.125	0.997
		[0.769, 1.553]	[0.759, 1.536]	[0.790, 1.601]	[0.790, 1.602]	[0.697, 1.425]
		s.e. = 0.196	s.e. = 0.194	s.e. = 0.203	s.e. = 0.203	s.e. = 0.182
		p = 0.621	p = 0.671	p = 0.516	p = 0.514	p = 0.985
	Medium	1.300	1.296	1.259	1.250	1.205
		[0.984, 1.718]	[0.981, 1.713]	[0.952, 1.665]	[0.945, 1.653]	[0.910, 1.596]
		s.e. = 0.185	s.e. = 0.184	s.e. = 0.180	s.e. = 0.178	s.e. = 0.173
		p = 0.065	p = 0.068	p = 0.106	p = 0.118	p = 0.194
image5 - Woman wearing mask	Low	0.812	0.784	0.811	0.812	1.098
		[0.687, 0.961]	[0.663, 0.928]	[0.684, 0.962]	[0.685, 0.963]	[0.921, 1.310]
		s.e. = 0.069	s.e. = 0.067	s.e. = 0.071	s.e. = 0.071	s.e. = 0.099
		p = 0.015	p = 0.005	p = 0.016	p = 0.017	p = 0.296
	Medium	1.002	0.993	0.953	0.949	1.071
		[0.875, 1.148]	[0.867, 1.138]	[0.830, 1.094]	[0.827, 1.090]	[0.929, 1.234]
		s.e. = 0.070	s.e. = 0.069	s.e. = 0.067	s.e. = 0.067	s.e. = 0.077
		p = 0.976	p = 0.921	p = 0.493	p = 0.460	p = 0.344
Image: Man wearing mask	Low	0.880	0.869	0.877	0.878	1.043
		[0.710, 1.091]	[0.701, 1.078]	[0.707, 1.088]	[0.708, 1.089]	[0.838, 1.299]
		s.e. = 0.096	s.e. = 0.095	s.e. = 0.097	s.e. = 0.097	s.e. = 0.117
		p = 0.243	p = 0.202	p = 0.233	p = 0.237	p = 0.706
	Medium	0.997	0.994	1.005	1.002	1.065
		[0.840, 1.184]	[0.837, 1.181]	[0.846, 1.195]	[0.843, 1.191]	[0.895, 1.268]
		s.e. = 0.087	s.e. = 0.087	s.e. = 0.089	s.e. = 0.088	s.e. = 0.095
		p = 0.974	p = 0.947	p = 0.951	p = 0.978	p = 0.476
Sex: Male	Low		1.508	1.498	1.496	1.618
			[1.366, 1.665]	[1.357, 1.654]	[1.354, 1.652]	[1.462, 1.791]
			s.e. = 0.076	s.e. = 0.076	s.e. = 0.076	s.e. = 0.084
			p = <0.001	p = <0.001	p = <0.001	p = <0.001
	Medium		1.117	1.130	1.135	1.171
			[1.033, 1.208]	[1.045, 1.222]	[1.049, 1.227]	[1.082, 1.267]
			s.e. = 0.044	s.e. = 0.045	s.e. = 0.045	s.e. = 0.047
			p = 0.005	p = 0.002	p = 0.002	p = <0.001
Age: 25- 44	Low			0.951	0.963	0.810
				[0.820, 1.103]	[0.828, 1.121]	[0.694, 0.946]
				s.e. = 0.072	s.e. = 0.074	s.e. = 0.064
				p = 0.507	p = 0.628	p = 0.008
	Medium			0.749	0.732	0.690
				[0.674, 0.832]	[0.657, 0.816]	[0.618, 0.769]
				s.e. = 0.040	s.e. = 0.040	s.e. = 0.038
				p = <0.001	p = <0.001	p = <0.001
Age: 45- 64	Low			1.163	1.174	0.936
				[0.999, 1.353]	[1.007, 1.369]	[0.799, 1.096]
				s.e. = 0.090	s.e. = 0.092	s.e. = 0.075
				p = 0.052	p = 0.041	p = 0.411
	Medium			0.731	0.722	0.672
				[0.654, 0.818]	[0.644, 0.808]	[0.599, 0.754]
				s.e. = 0.042	s.e. = 0.042	s.e. = 0.039
				p = <0.001	p = <0.001	p = <0.001
Age: 65+	Low			0.998	1.008	0.803
				[0.831, 1.198]	[0.838, 1.213]	[0.665, 0.970]
				s.e. = 0.093	s.e. = 0.095	s.e. = 0.077
				p = 0.980	p = 0.931	p = 0.023
	Medium			0.680	0.669	0.623
				[0.593, 0.780]	[0.583, 0.768]	[0.542, 0.717]
				s.e. = 0.047	s.e. = 0.047	s.e. = 0.044
				p = <0.001	p = <0.001	p = <0.001
Education: University level	Low				0.979	0.981
					[0.881, 1.088]	[0.881, 1.091]
					s.e. = 0.053	s.e. = 0.053
					p = 0.696	p = 0.720
	Medium				1.083	1.082
					[0.999, 1.173]	[0.999, 1.173]
					s.e. = 0.044	s.e. = 0.044
					p = 0.053	p = 0.054
Education: Postgraduate Degree	Low				0.927	0.932
					[0.783, 1.099]	[0.784, 1.107]
					s.e. = 0.080	s.e. = 0.082
					p = 0.384	p = 0.422
	Medium				1.053	1.055
					[0.926, 1.198]	[0.927, 1.201]

	response					
					s.e. = 0.069	s.e. = 0.070
					p = 0.430	p = 0.416
Month: April	Low					1.857
						[1.634, 2.111]
						s.e. = 0.121
						p = <0.001
	Medium					1.303
						[1.188, 1.429]
						s.e. = 0.061
						p = <0.001
Month: May	Low					3.295
						[2.881, 3.769]
						s.e. = 0.226
						p = <0.001
	Medium					1.582
						[1.421, 1.760]
						s.e. = 0.086
						p = <0.001
Month: June	Low					3.494
						[2.780, 4.391]
						s.e. = 0.407
						p = <0.001
	Medium					1.484
						[1.214, 1.815]
						s.e. = 0.152
						p = <0.001
Month: July	Low					3.005
						[2.452, 3.682]
						s.e. = 0.312
						p = <0.001
	Medium					1.394
						[1.170, 1.662]
						s.e. = 0.125
						p = <0.001
Month: August	Low					1.940
						[1.404, 2.681]
						s.e. = 0.320
						p = <0.001
	Medium					1.437
						[1.125, 1.836]
						s.e. = 0.180
						p = 0.004
Num.Obs.		14202	14202	14202	14202	14202
AIC		28553.4	28491.8	28441.1	28443.3	28066.9
Odds Ratios and 95% confidence intervals.						

Table 21 Step wise regression results for the Netherlands, Outcome: Threat perception of COVID-19 to the family

	response					
(Intercept)	Low	0.812	0.694	0.601	0.602	0.507
		[0.715, 0.921]	[0.609, 0.792]	[0.479, 0.754]	[0.480, 0.756]	[0.401, 0.641]
		s.e. = 0.052	s.e. = 0.047	s.e. = 0.069	s.e. = 0.070	s.e. = 0.061
		p = 0.001	p = <0.001	p = <0.001	p = <0.001	p = <0.001
	Medium	1.022	1.015	1.239	1.218	1.187
		[0.908, 1.152]	[0.898, 1.147]	[1.009, 1.522]	[0.990, 1.497]	[0.964, 1.462]
		s.e. = 0.062	s.e. = 0.063	s.e. = 0.130	s.e. = 0.129	s.e. = 0.126
		p = 0.715	p = 0.811	p = 0.041	p = 0.062	p = 0.106
Image: Couple blowing nose	Low	1.590	1.563	1.619	1.624	1.052
		[1.115, 2.267]	[1.094, 2.232]	[1.131, 2.317]	[1.134, 2.324]	[0.726, 1.525]
		s.e. = 0.288	s.e. = 0.284	s.e. = 0.296	s.e. = 0.297	s.e. = 0.199
		p = 0.010	p = 0.014	p = 0.008	p = 0.008	p = 0.789
	Medium	1.231	1.229	1.189	1.186	1.025
		[0.864, 1.753]	[0.863, 1.752]	[0.832, 1.697]	[0.831, 1.694]	[0.715, 1.470]
		s.e. = 0.222	s.e. = 0.222	s.e. = 0.216	s.e. = 0.216	s.e. = 0.189
		p = 0.251	p = 0.253	p = 0.342	p = 0.348	p = 0.892
image5 - Woman wearing mask	Low	1.192	1.116	1.170	1.175	1.006
		[1.031, 1.378]	[0.963, 1.292]	[1.005, 1.361]	[1.009, 1.370]	[0.858, 1.179]
		s.e. = 0.088	s.e. = 0.083	s.e. = 0.090	s.e. = 0.092	s.e. = 0.082

	response					
		p = 0.018	p = 0.144	p = 0.043	p = 0.038	p = 0.945
	Medium	1.029	1.026	1.034	1.021	0.971
		[0.897, 1.182]	[0.893, 1.178]	[0.895, 1.195]	[0.883, 1.182]	[0.838, 1.126]
		s.e. = 0.072	s.e. = 0.073	s.e. = 0.076	s.e. = 0.076	s.e. = 0.073
		p = 0.681	p = 0.718	p = 0.647	p = 0.775	p = 0.699
Image: Man wearing mask	Low	1.226	1.211	1.211	1.213	1.036
		[1.032, 1.456]	[1.019, 1.439]	[1.018, 1.439]	[1.020, 1.442]	[0.864, 1.241]
		s.e. = 0.107	s.e. = 0.107	s.e. = 0.107	s.e. = 0.107	s.e. = 0.096
		p = 0.020	p = 0.030	p = 0.030	p = 0.029	p = 0.704
	Medium	1.179	1.179	1.152	1.146	1.089
		[1.002, 1.388]	[1.001, 1.387]	[0.978, 1.356]	[0.973, 1.350]	[0.922, 1.286]
		s.e. = 0.098	s.e. = 0.098	s.e. = 0.096	s.e. = 0.096	s.e. = 0.092
		p = 0.047	p = 0.048	p = 0.090	p = 0.101	p = 0.315
Sex: Male	Low		1.656	1.623	1.627	1.572
			[1.480, 1.853]	[1.449, 1.818]	[1.452, 1.823]	[1.398, 1.768]
			s.e. = 0.095	s.e. = 0.094	s.e. = 0.094	s.e. = 0.094
			p = <0.001	p = <0.001	p = <0.001	p = <0.001
	Medium		1.028	1.025	1.023	1.010
			[0.920, 1.149]	[0.915, 1.147]	[0.913, 1.145]	[0.902, 1.132]
			s.e. = 0.058	s.e. = 0.059	s.e. = 0.059	s.e. = 0.059
			p = 0.626	p = 0.671	p = 0.696	p = 0.863
Age: 25- 44	Low			1.020	1.028	0.895
				[0.824, 1.262]	[0.830, 1.273]	[0.718, 1.116]
				s.e. = 0.111	s.e. = 0.112	s.e. = 0.101
				p = 0.856	p = 0.803	p = 0.324
	Medium			0.725	0.722	0.694
				[0.597, 0.880]	[0.594, 0.877]	[0.571, 0.845]
				s.e. = 0.072	s.e. = 0.072	s.e. = 0.069
				p = 0.001	p = 0.001	p = <0.001
Age: 45- 64	Low			1.183	1.183	0.940
				[0.969, 1.445]	[0.968, 1.446]	[0.764, 1.155]
				s.e. = 0.121	s.e. = 0.121	s.e. = 0.099
				p = 0.099	p = 0.100	p = 0.554
	Medium			0.780	0.788	0.736
				[0.651, 0.936]	[0.657, 0.946]	[0.612, 0.885]
				s.e. = 0.072	s.e. = 0.073	s.e. = 0.069
				p = 0.007	p = 0.011	p = 0.001
Age: 65+	Low			1.199	1.200	0.939
				[0.968, 1.484]	[0.968, 1.486]	[0.753, 1.171]
				s.e. = 0.131	s.e. = 0.131	s.e. = 0.106
				p = 0.096	p = 0.096	p = 0.574
	Medium			0.900	0.912	0.845
				[0.741, 1.094]	[0.750, 1.108]	[0.693, 1.029]
				s.e. = 0.089	s.e. = 0.091	s.e. = 0.085
				p = 0.291	p = 0.354	p = 0.094
Education: University level	Low				0.980	0.866
					[0.858, 1.119]	[0.755, 0.994]
					s.e. = 0.066	s.e. = 0.061
					p = 0.768	p = 0.041
	Medium				1.096	1.055
					[0.965, 1.244]	[0.928, 1.199]
					s.e. = 0.071	s.e. = 0.069
					p = 0.159	p = 0.414
Education: Postgraduate Degree	Low				0.858	0.700
					[0.571, 1.289]	[0.458, 1.068]
					s.e. = 0.178	s.e. = 0.151
					p = 0.461	p = 0.098
	Medium				0.876	0.826
					[0.584, 1.313]	[0.549, 1.241]
					s.e. = 0.181	s.e. = 0.172
					p = 0.522	p = 0.357
Month: May	Low					2.573
						[2.245, 2.950]
						s.e. = 0.179
						p = <0.001
	Medium					1.353
						[1.185, 1.543]
						s.e. = 0.091
						p = <0.001
Month: June	Low					5.892
						[4.588, 7.567]

	response					
						s.e. = 0.752
						p = <0.001
	Medium					1.991
						[1.527, 2.598]
						s.e. = 0.270
						p = <0.001
Month: July	Low					4.424
						[3.608, 5.426]
						s.e. = 0.461
						p = <0.001
	Medium					1.534
						[1.235, 1.905]
						s.e. = 0.170
						p = <0.001
Month: August	Low					3.009
						[2.177, 4.158]
						s.e. = 0.496
						p = <0.001
	Medium					1.640
						[1.183, 2.274]
						s.e. = 0.274
						p = 0.003
Num.Obs.		8223	8223	8223	8223	8223
AIC		18044.7	17948.8	17933.0	17936.9	17447.6
Odds Ratios and 95% confidence intervals.						

Table 22 Step wise regression results for Spain, Outcome: Threat perception of COVID-19 to the family

	response					
(Intercept)	Low	0.640	0.610	0.530	0.616	0.520
		[0.550, 0.744]	[0.523, 0.712]	[0.407, 0.688]	[0.471, 0.806]	[0.390, 0.693]
		s.e. = 0.049	s.e. = 0.048	s.e. = 0.071	s.e. = 0.085	s.e. = 0.076
		p = <0.001	p = <0.001	p = <0.001	p = <0.001	p = <0.001
	Medium	0.995	0.996	1.010	1.045	0.964
		[0.871, 1.138]	[0.869, 1.141]	[0.810, 1.260]	[0.832, 1.312]	[0.756, 1.228]
		s.e. = 0.068	s.e. = 0.069	s.e. = 0.114	s.e. = 0.121	s.e. = 0.119
		p = 0.946	p = 0.955	p = 0.928	p = 0.705	p = 0.765
Image: Couple blowing nose	Low	1.029	1.018	1.051	1.057	0.954
		[0.741, 1.429]	[0.733, 1.414]	[0.756, 1.461]	[0.760, 1.470]	[0.684, 1.331]
		s.e. = 0.172	s.e. = 0.171	s.e. = 0.177	s.e. = 0.178	s.e. = 0.162
		p = 0.866	p = 0.914	p = 0.767	p = 0.741	p = 0.783
	Medium	0.969	0.970	1.000	1.001	0.955
		[0.722, 1.301]	[0.722, 1.301]	[0.744, 1.343]	[0.745, 1.344]	[0.710, 1.285]
		s.e. = 0.145	s.e. = 0.146	s.e. = 0.151	s.e. = 0.151	s.e. = 0.145
		p = 0.836	p = 0.837	p = 0.998	p = 0.996	p = 0.763
image5 - Woman wearing mask	Low	0.798	0.792	0.841	0.869	0.908
		[0.678, 0.940]	[0.673, 0.933]	[0.713, 0.993]	[0.736, 1.026]	[0.768, 1.074]
		s.e. = 0.066	s.e. = 0.066	s.e. = 0.071	s.e. = 0.074	s.e. = 0.078
		p = 0.007	p = 0.005	p = 0.041	p = 0.098	p = 0.261
	Medium	0.825	0.825	0.880	0.887	0.902
		[0.714, 0.952]	[0.714, 0.953]	[0.760, 1.019]	[0.766, 1.027]	[0.778, 1.045]
		s.e. = 0.061	s.e. = 0.061	s.e. = 0.066	s.e. = 0.066	s.e. = 0.068
		p = 0.009	p = 0.009	p = 0.087	p = 0.108	p = 0.170
Image: Man wearing mask	Low	0.899	0.892	0.881	0.896	0.901
		[0.748, 1.080]	[0.742, 1.072]	[0.733, 1.059]	[0.745, 1.077]	[0.749, 1.085]
		s.e. = 0.084	s.e. = 0.083	s.e. = 0.083	s.e. = 0.084	s.e. = 0.085
		p = 0.254	p = 0.222	p = 0.177	p = 0.243	p = 0.271
	Medium	0.953	0.953	0.941	0.944	0.944
		[0.811, 1.119]	[0.811, 1.119]	[0.800, 1.105]	[0.803, 1.110]	[0.803, 1.111]
		s.e. = 0.078	s.e. = 0.078	s.e. = 0.077	s.e. = 0.078	s.e. = 0.078
		p = 0.554	p = 0.555	p = 0.458	p = 0.485	p = 0.488
Sex: Male	Low		1.181	1.127	1.109	1.127
			[1.064, 1.311]	[1.013, 1.254]	[0.996, 1.234]	[1.012, 1.254]
			s.e. = 0.063	s.e. = 0.061	s.e. = 0.061	s.e. = 0.062
			p = 0.002	p = 0.028	p = 0.059	p = 0.030
	Medium		0.997	0.949	0.945	0.951
			[0.909, 1.094]	[0.863, 1.043]	[0.860, 1.039]	[0.865, 1.045]
			s.e. = 0.047	s.e. = 0.046	s.e. = 0.046	s.e. = 0.046

	response					
			p = 0.952	p = 0.274	p = 0.244	p = 0.297
Age: 25- 44	Low			0.995	1.021	0.973
				[0.791, 1.252]	[0.811, 1.285]	[0.772, 1.226]
				s.e. = 0.117	s.e. = 0.120	s.e. = 0.115
				p = 0.968	p = 0.860	p = 0.817
	Medium			0.819	0.825	0.807
				[0.677, 0.990]	[0.682, 0.997]	[0.667, 0.977]
				s.e. = 0.079	s.e. = 0.080	s.e. = 0.079
				p = 0.039	p = 0.047	p = 0.028
Age: 45- 64	Low			1.174	1.176	1.106
				[0.935, 1.473]	[0.936, 1.477]	[0.879, 1.391]
				s.e. = 0.136	s.e. = 0.137	s.e. = 0.129
				p = 0.168	p = 0.163	p = 0.389
	Medium			1.005	1.007	0.979
				[0.833, 1.213]	[0.834, 1.215]	[0.810, 1.183]
				s.e. = 0.097	s.e. = 0.097	s.e. = 0.095
				p = 0.959	p = 0.945	p = 0.827
Age: 65+	Low			1.381	1.436	1.342
				[1.074, 1.775]	[1.117, 1.848]	[1.042, 1.729]
				s.e. = 0.177	s.e. = 0.185	s.e. = 0.173
				p = 0.012	p = 0.005	p = 0.023
	Medium			1.197	1.209	1.174
				[0.970, 1.477]	[0.979, 1.493]	[0.950, 1.452]
				s.e. = 0.128	s.e. = 0.130	s.e. = 0.127
				p = 0.095	p = 0.078	p = 0.137
Education: University level	Low				0.769	0.773
					[0.691, 0.855]	[0.694, 0.860]
					s.e. = 0.042	s.e. = 0.042
					p = <0.001	p = <0.001
	Medium				0.946	0.948
					[0.860, 1.040]	[0.862, 1.042]
					s.e. = 0.046	s.e. = 0.046
					p = 0.249	p = 0.268
Education: Postgraduate Degree	Low				0.652	0.648
					[0.543, 0.784]	[0.539, 0.779]
					s.e. = 0.061	s.e. = 0.061
					p = <0.001	p = <0.001
	Medium				0.898	0.895
					[0.769, 1.047]	[0.767, 1.045]
					s.e. = 0.071	s.e. = 0.071
					p = 0.170	p = 0.160
Month: April	Low					1.092
						[0.946, 1.259]
						s.e. = 0.080
						p = 0.228
	Medium					1.068
						[0.948, 1.204]
						s.e. = 0.065
						p = 0.279
Month: May	Low					1.362
						[1.153, 1.608]
						s.e. = 0.115
						p = <0.001
	Medium					1.134
						[0.983, 1.309]
						s.e. = 0.083
						p = 0.085
Month: June	Low					1.729
						[1.354, 2.207]
						s.e. = 0.215
						p = <0.001
	Medium					1.285
						[1.031, 1.602]
						s.e. = 0.145
						p = 0.026
Month: July	Low					1.804
						[1.434, 2.270]
						s.e. = 0.211
						p = <0.001
	Medium					1.310
						[1.064, 1.613]

	response					
						s.e. = 0.139
						p = 0.011
Month: August	Low					1.989
						[1.425, 2.776]
						s.e. = 0.338
						p = <0.001
	Medium					1.415
						[1.038, 1.929]
						s.e. = 0.224
						p = 0.028
Num.Obs.		10964	10964	10964	10964	10964
AIC		23418.5	23410.7	23383.7	23358.1	23319.7
Odds Ratios and 95% confidence intervals.						

Table 23 Step wise regression results for the United Kingdom, Outcome: Threat perception of COVID-19 to the family

	response					
(Intercept)	Low	0.357	0.303	0.477	0.483	0.327
		[0.296, 0.431]	[0.250, 0.368]	[0.363, 0.627]	[0.366, 0.637]	[0.245, 0.438]
		s.e. = 0.034	s.e. = 0.030	s.e. = 0.067	s.e. = 0.068	s.e. = 0.048
		p = <0.001	p = <0.001	p = <0.001	p = <0.001	p = <0.001
	Medium	0.785	0.760	0.860	0.850	0.720
		[0.678, 0.908]	[0.655, 0.881]	[0.687, 1.076]	[0.678, 1.067]	[0.571, 0.910]
		s.e. = 0.059	s.e. = 0.057	s.e. = 0.098	s.e. = 0.099	s.e. = 0.086
		p = 0.001	p = <0.001	p = 0.186	p = 0.162	p = 0.006
Image: Couple blowing nose	Low	1.828	1.786	1.675	1.674	1.296
		[1.261, 2.651]	[1.230, 2.594]	[1.152, 2.435]	[1.151, 2.435]	[0.882, 1.902]
		s.e. = 0.347	s.e. = 0.340	s.e. = 0.320	s.e. = 0.320	s.e. = 0.254
		p = 0.001	p = 0.002	p = 0.007	p = 0.007	p = 0.186
	Medium	1.368	1.361	1.350	1.344	1.210
		[0.998, 1.875]	[0.993, 1.866]	[0.984, 1.852]	[0.979, 1.844]	[0.880, 1.663]
		s.e. = 0.220	s.e. = 0.219	s.e. = 0.218	s.e. = 0.217	s.e. = 0.197
		p = 0.051	p = 0.055	p = 0.063	p = 0.067	p = 0.242
image5 - Woman wearing mask	Low	1.052	1.011	0.972	0.973	1.152
		[0.863, 1.283]	[0.829, 1.234]	[0.795, 1.188]	[0.795, 1.190]	[0.937, 1.417]
		s.e. = 0.106	s.e. = 0.103	s.e. = 0.100	s.e. = 0.100	s.e. = 0.122
		p = 0.615	p = 0.912	p = 0.778	p = 0.789	p = 0.180
	Medium	0.986	0.978	1.003	0.993	1.067
		[0.846, 1.150]	[0.838, 1.140]	[0.859, 1.171]	[0.850, 1.160]	[0.912, 1.249]
		s.e. = 0.077	s.e. = 0.077	s.e. = 0.079	s.e. = 0.079	s.e. = 0.086
		p = 0.857	p = 0.773	p = 0.973	p = 0.926	p = 0.417
Image: Man wearing mask	Low	1.021	0.963	0.951	0.950	1.010
		[0.809, 1.289]	[0.762, 1.218]	[0.752, 1.202]	[0.751, 1.202]	[0.794, 1.284]
		s.e. = 0.121	s.e. = 0.115	s.e. = 0.114	s.e. = 0.114	s.e. = 0.124
		p = 0.862	p = 0.755	p = 0.672	p = 0.670	p = 0.935
	Medium	0.965	0.953	0.936	0.929	0.931
		[0.805, 1.157]	[0.795, 1.143]	[0.781, 1.123]	[0.774, 1.114]	[0.775, 1.119]
		s.e. = 0.089	s.e. = 0.088	s.e. = 0.087	s.e. = 0.086	s.e. = 0.087
		p = 0.701	p = 0.604	p = 0.479	p = 0.426	p = 0.446
Sex: Male	Low		1.699	1.722	1.721	1.728
			[1.529, 1.889]	[1.549, 1.916]	[1.547, 1.914]	[1.550, 1.926]
			s.e. = 0.092	s.e. = 0.093	s.e. = 0.093	s.e. = 0.096
			p = <0.001	p = <0.001	p = <0.001	p = <0.001
	Medium		1.130	1.117	1.115	1.122
			[1.036, 1.232]	[1.024, 1.218]	[1.022, 1.216]	[1.029, 1.225]
			s.e. = 0.050	s.e. = 0.050	s.e. = 0.049	s.e. = 0.050
			p = 0.006	p = 0.013	p = 0.014	p = 0.010
Age: 25- 44	Low			0.733	0.733	0.682
				[0.590, 0.912]	[0.588, 0.913]	[0.546, 0.852]
				s.e. = 0.082	s.e. = 0.082	s.e. = 0.078
				p = 0.005	p = 0.006	p = <0.001
	Medium			0.794	0.781	0.758
				[0.657, 0.958]	[0.646, 0.944]	[0.627, 0.917]
				s.e. = 0.076	s.e. = 0.076	s.e. = 0.074
				p = 0.016	p = 0.011	p = 0.004
Age: 45- 64	Low			0.565	0.564	0.467
				[0.459, 0.696]	[0.458, 0.694]	[0.377, 0.578]
				s.e. = 0.060	s.e. = 0.060	s.e. = 0.051

	response					
				p = <0.001	p = <0.001	p = <0.001
	Medium			0.821	0.816	0.760
				[0.687, 0.981]	[0.683, 0.975]	[0.635, 0.909]
				s.e. = 0.075	s.e. = 0.074	s.e. = 0.070
				p = 0.030	p = 0.025	p = 0.003
Age: 65+	Low			0.665	0.663	0.526
				[0.536, 0.826]	[0.534, 0.823]	[0.421, 0.656]
				s.e. = 0.073	s.e. = 0.073	s.e. = 0.060
				p = <0.001	p = <0.001	p = <0.001
	Medium			0.997	0.995	0.917
				[0.829, 1.199]	[0.828, 1.197]	[0.762, 1.105]
				s.e. = 0.094	s.e. = 0.094	s.e. = 0.087
				p = 0.974	p = 0.959	p = 0.363
Education: University level	Low				0.972	0.939
					[0.870, 1.084]	[0.840, 1.051]
					s.e. = 0.055	s.e. = 0.054
					p = 0.607	p = 0.274
	Medium				1.035	1.026
					[0.949, 1.129]	[0.940, 1.119]
					s.e. = 0.046	s.e. = 0.046
					p = 0.437	p = 0.569
Education: Postgraduate Degree	Low				1.041	1.014
					[0.849, 1.276]	[0.824, 1.248]
					s.e. = 0.108	s.e. = 0.107
					p = 0.700	p = 0.896
	Medium				1.148	1.143
					[0.978, 1.347]	[0.973, 1.342]
					s.e. = 0.094	s.e. = 0.094
					p = 0.091	p = 0.104
Month: April	Low					1.237
						[1.070, 1.429]
						s.e. = 0.091
						p = 0.004
	Medium					1.198
						[1.080, 1.329]
						s.e. = 0.064
						p = <0.001
Month: May	Low					2.357
						[2.034, 2.731]
						s.e. = 0.177
						p = <0.001
	Medium					1.464
						[1.301, 1.648]
						s.e. = 0.088
						p = <0.001
Month: June	Low					3.545
						[2.810, 4.471]
						s.e. = 0.420
						p = <0.001
	Medium					1.653
						[1.344, 2.032]
						s.e. = 0.174
						p = <0.001
Month: July	Low					3.832
						[3.121, 4.705]
						s.e. = 0.401
						p = <0.001
	Medium					1.786
						[1.487, 2.145]
						s.e. = 0.167
						p = <0.001
Month: August	Low					5.659
						[4.072, 7.863]
						s.e. = 0.950
						p = <0.001
	Medium					1.751
						[1.259, 2.437]
						s.e. = 0.295
						p = <0.001
Num.Obs.		11401	11401	11401	11401	11401
AIC		23472.0	23379.6	23335.3	23339.6	22982.6

	response					
Odds Ratios and 95% confidence intervals.						

Table 24 Step wise regression results for the United States, Outcome: Threat perception of COVID-19 to the family

	response					
(Intercept)	Low	1.369	1.027	0.818	0.955	0.559
		[1.160, 1.617]	[0.867, 1.217]	[0.671, 0.998]	[0.781, 1.168]	[0.453, 0.690]
		s.e. = 0.116	s.e. = 0.089	s.e. = 0.083	s.e. = 0.098	s.e. = 0.060
		p = <0.001	p = 0.756	p = 0.048	p = 0.655	p = <0.001
	Medium	1.116	1.091	1.134	1.168	1.085
		[0.938, 1.328]	[0.916, 1.299]	[0.930, 1.384]	[0.955, 1.428]	[0.882, 1.335]
		s.e. = 0.099	s.e. = 0.097	s.e. = 0.115	s.e. = 0.120	s.e. = 0.115
		p = 0.215	p = 0.327	p = 0.214	p = 0.130	p = 0.440
Image: Couple blowing nose	Low	0.736	0.705	0.749	0.756	0.744
		[0.547, 0.991]	[0.521, 0.954]	[0.553, 1.014]	[0.558, 1.024]	[0.548, 1.009]
		s.e. = 0.112	s.e. = 0.109	s.e. = 0.116	s.e. = 0.117	s.e. = 0.116
		p = 0.043	p = 0.023	p = 0.061	p = 0.071	p = 0.057
	Medium	1.010	1.006	1.010	1.009	1.000
		[0.751, 1.358]	[0.748, 1.353]	[0.750, 1.358]	[0.750, 1.358]	[0.743, 1.346]
		s.e. = 0.153	s.e. = 0.152	s.e. = 0.153	s.e. = 0.153	s.e. = 0.152
		p = 0.949	p = 0.970	p = 0.950	p = 0.952	p = 0.998
image5 - Woman wearing mask	Low	0.764	0.716	0.738	0.743	0.897
		[0.645, 0.904]	[0.603, 0.850]	[0.621, 0.876]	[0.625, 0.883]	[0.752, 1.071]
		s.e. = 0.066	s.e. = 0.063	s.e. = 0.065	s.e. = 0.065	s.e. = 0.081
		p = 0.002	p = <0.001	p = <0.001	p = <0.001	p = 0.229
	Medium	0.869	0.864	0.871	0.871	0.895
		[0.729, 1.037]	[0.724, 1.031]	[0.729, 1.039]	[0.730, 1.040]	[0.748, 1.071]
		s.e. = 0.078	s.e. = 0.078	s.e. = 0.079	s.e. = 0.079	s.e. = 0.082
		p = 0.121	p = 0.106	p = 0.125	p = 0.127	p = 0.228
Image: Man wearing mask	Low	0.732	0.679	0.679	0.684	0.761
		[0.608, 0.880]	[0.563, 0.819]	[0.563, 0.819]	[0.566, 0.826]	[0.629, 0.922]
		s.e. = 0.069	s.e. = 0.065	s.e. = 0.065	s.e. = 0.066	s.e. = 0.074
		p = <0.001	p = <0.001	p = <0.001	p = <0.001	p = 0.005
	Medium	0.909	0.902	0.900	0.900	0.908
		[0.750, 1.101]	[0.745, 1.093]	[0.743, 1.090]	[0.743, 1.090]	[0.749, 1.102]
		s.e. = 0.089	s.e. = 0.088	s.e. = 0.088	s.e. = 0.088	s.e. = 0.090
		p = 0.328	p = 0.294	p = 0.282	p = 0.281	p = 0.329
Sex: Male	Low		2.521	2.462	2.417	2.410
			[2.374, 2.678]	[2.317, 2.615]	[2.275, 2.569]	[2.265, 2.564]
			s.e. = 0.077	s.e. = 0.076	s.e. = 0.075	s.e. = 0.076
			p = <0.001	p = <0.001	p = <0.001	p = <0.001
	Medium		1.105	1.100	1.097	1.099
			[1.037, 1.178]	[1.032, 1.173]	[1.028, 1.169]	[1.031, 1.172]
			s.e. = 0.036	s.e. = 0.036	s.e. = 0.036	s.e. = 0.036
			p = 0.002	p = 0.003	p = 0.005	p = 0.004
Age: 25- 44	Low			1.030	1.166	1.070
				[0.913, 1.162]	[1.032, 1.317]	[0.946, 1.211]
				s.e. = 0.063	s.e. = 0.072	s.e. = 0.067
				p = 0.629	p = 0.014	p = 0.280
	Medium			0.923	0.940	0.928
				[0.825, 1.033]	[0.840, 1.053]	[0.828, 1.040]
				s.e. = 0.053	s.e. = 0.054	s.e. = 0.054
				p = 0.162	p = 0.288	p = 0.201
Age: 45- 64	Low			1.395	1.532	1.314
				[1.244, 1.565]	[1.364, 1.720]	[1.168, 1.479]
				s.e. = 0.082	s.e. = 0.091	s.e. = 0.079
				p = <0.001	p = <0.001	p = <0.001
	Medium			0.945	0.958	0.937
				[0.847, 1.053]	[0.859, 1.069]	[0.839, 1.046]
				s.e. = 0.052	s.e. = 0.053	s.e. = 0.053
				p = 0.304	p = 0.443	p = 0.249
Age: 65+	Low			1.287	1.444	1.218
				[1.145, 1.447]	[1.283, 1.626]	[1.080, 1.375]
				s.e. = 0.077	s.e. = 0.087	s.e. = 0.075
				p = <0.001	p = <0.001	p = 0.001
	Medium			0.989	1.005	0.982
				[0.885, 1.104]	[0.899, 1.123]	[0.877, 1.099]
				s.e. = 0.056	s.e. = 0.057	s.e. = 0.056

	response					
				p = 0.843	p = 0.934	p = 0.752
Education: University level	Low				0.674	0.665
					[0.634, 0.717]	[0.626, 0.708]
					s.e. = 0.021	s.e. = 0.021
					p = <0.001	p = <0.001
	Medium				0.937	0.934
					[0.880, 0.997]	[0.878, 0.994]
					s.e. = 0.030	s.e. = 0.030
					p = 0.039	p = 0.031
Education: Postgraduate Degree	Low				0.485	0.484
					[0.423, 0.557]	[0.421, 0.556]
					s.e. = 0.034	s.e. = 0.034
					p = <0.001	p = <0.001
	Medium				0.930	0.927
					[0.819, 1.056]	[0.816, 1.053]
					s.e. = 0.060	s.e. = 0.060
					p = 0.263	p = 0.243
Month: April	Low					1.361
						[1.256, 1.475]
						s.e. = 0.056
						p = <0.001
	Medium					1.055
						[0.980, 1.136]
						s.e. = 0.040
						p = 0.154
Month: May	Low					2.732
						[2.508, 2.976]
						s.e. = 0.119
						p = <0.001
	Medium					1.171
						[1.076, 1.275]
						s.e. = 0.051
						p = <0.001
Month: June	Low					2.623
						[2.256, 3.050]
						s.e. = 0.202
						p = <0.001
	Medium					1.181
						[1.012, 1.379]
						s.e. = 0.093
						p = 0.035
Month: July	Low					2.075
						[1.853, 2.324]
						s.e. = 0.120
						p = <0.001
	Medium					1.120
						[1.000, 1.255]
						s.e. = 0.065
						p = 0.049
Month: August	Low					2.190
						[1.803, 2.660]
						s.e. = 0.217
						p = <0.001
	Medium					0.978
						[0.792, 1.208]
						s.e. = 0.105
						p = 0.836
Num.Obs.		28400	28400	28400	28400	28400
AIC		62378.8	61225.3	61132.6	60889.4	60121.9
Odds Ratios and 95% confidence intervals.						

Table 25 Step wise regression results for Belgium, Outcome: Threat perception of COVID-19 to oneself

	response					
(Intercept)	Low	0.853	0.745	3.251	3.153	2.408
		[0.727, 1.001]	[0.632, 0.878]	[2.593, 4.075]	[2.511, 3.958]	[1.905, 3.043]
		s.e. = 0.070	s.e. = 0.062	s.e. = 0.375	s.e. = 0.366	s.e. = 0.288
		p = 0.051	p = <0.001	p = <0.001	p = <0.001	p = <0.001

	response					
	Medium	0.829	0.853	1.719	1.679	1.547
		[0.705, 0.974]	[0.724, 1.004]	[1.362, 2.171]	[1.328, 2.123]	[1.219, 1.963]
		s.e. = 0.068	s.e. = 0.071	s.e. = 0.205	s.e. = 0.201	s.e. = 0.188
		p = 0.022	p = 0.057	p = <0.001	p = <0.001	p = <0.001
Image: Couple blowing nose	Low	1.688	1.696	1.205	1.192	1.005
		[1.137, 2.504]	[1.142, 2.519]	[0.801, 1.814]	[0.792, 1.794]	[0.662, 1.524]
		s.e. = 0.340	s.e. = 0.342	s.e. = 0.251	s.e. = 0.249	s.e. = 0.214
		p = 0.009	p = 0.009	p = 0.371	p = 0.400	p = 0.983
	Medium	1.424	1.422	1.266	1.251	1.172
		[0.945, 2.145]	[0.944, 2.143]	[0.838, 1.912]	[0.828, 1.891]	[0.774, 1.774]
		s.e. = 0.298	s.e. = 0.297	s.e. = 0.266	s.e. = 0.264	s.e. = 0.248
		p = 0.091	p = 0.092	p = 0.263	p = 0.287	p = 0.453
image5 - Woman wearing mask	Low	1.540	1.527	1.135	1.099	1.197
		[1.299, 1.825]	[1.288, 1.811]	[0.951, 1.354]	[0.920, 1.314]	[0.998, 1.437]
		s.e. = 0.134	s.e. = 0.133	s.e. = 0.102	s.e. = 0.100	s.e. = 0.111
		p = <0.001	p = <0.001	p = 0.162	p = 0.299	p = 0.053
	Medium	1.435	1.438	1.312	1.275	1.300
		[1.209, 1.704]	[1.211, 1.707]	[1.101, 1.564]	[1.068, 1.521]	[1.088, 1.554]
		s.e. = 0.126	s.e. = 0.126	s.e. = 0.117	s.e. = 0.115	s.e. = 0.118
		p = <0.001	p = <0.001	p = 0.002	p = 0.007	p = 0.004
Image: Man wearing mask	Low	0.861	0.881	0.868	0.859	0.975
		[0.717, 1.034]	[0.734, 1.059]	[0.720, 1.045]	[0.713, 1.035]	[0.806, 1.179]
		s.e. = 0.080	s.e. = 0.082	s.e. = 0.082	s.e. = 0.082	s.e. = 0.095
		p = 0.108	p = 0.177	p = 0.135	p = 0.110	p = 0.791
	Medium	1.168	1.162	1.159	1.149	1.194
		[0.975, 1.399]	[0.969, 1.392]	[0.967, 1.389]	[0.959, 1.378]	[0.994, 1.433]
		s.e. = 0.108	s.e. = 0.107	s.e. = 0.107	s.e. = 0.106	s.e. = 0.111
		p = 0.093	p = 0.105	p = 0.111	p = 0.133	p = 0.057
Sex: Male	Low		1.450	1.718	1.729	1.706
			[1.316, 1.597]	[1.554, 1.899]	[1.563, 1.912]	[1.540, 1.889]
			s.e. = 0.072	s.e. = 0.088	s.e. = 0.089	s.e. = 0.089
			p = <0.001	p = <0.001	p = <0.001	p = <0.001
	Medium		0.913	0.961	0.965	0.963
			[0.827, 1.009]	[0.869, 1.064]	[0.872, 1.068]	[0.870, 1.067]
			s.e. = 0.047	s.e. = 0.050	s.e. = 0.050	s.e. = 0.050
			p = 0.075	p = 0.447	p = 0.493	p = 0.471
Age: 25- 44	Low			0.293	0.284	0.261
				[0.246, 0.349]	[0.238, 0.339]	[0.219, 0.312]
				s.e. = 0.026	s.e. = 0.026	s.e. = 0.024
				p = <0.001	p = <0.001	p = <0.001
	Medium			0.527	0.512	0.498
				[0.438, 0.634]	[0.425, 0.617]	[0.413, 0.600]
				s.e. = 0.050	s.e. = 0.049	s.e. = 0.047
				p = <0.001	p = <0.001	p = <0.001
Age: 45- 64	Low			0.188	0.185	0.160
				[0.158, 0.224]	[0.156, 0.221]	[0.134, 0.191]
				s.e. = 0.017	s.e. = 0.016	s.e. = 0.014
				p = <0.001	p = <0.001	p = <0.001
	Medium			0.467	0.460	0.437
				[0.389, 0.560]	[0.383, 0.552]	[0.364, 0.525]
				s.e. = 0.043	s.e. = 0.043	s.e. = 0.041
				p = <0.001	p = <0.001	p = <0.001
Age: 65+	Low			0.176	0.173	0.147
				[0.146, 0.213]	[0.143, 0.209]	[0.121, 0.178]
				s.e. = 0.017	s.e. = 0.017	s.e. = 0.015
				p = <0.001	p = <0.001	p = <0.001
	Medium			0.468	0.458	0.432
				[0.385, 0.569]	[0.376, 0.557]	[0.354, 0.526]
				s.e. = 0.047	s.e. = 0.046	s.e. = 0.044
				p = <0.001	p = <0.001	p = <0.001
Education: University level	Low				1.142	1.092
					[1.035, 1.259]	[0.989, 1.206]
					s.e. = 0.057	s.e. = 0.055
					p = 0.008	p = 0.083
	Medium				1.118	1.098
					[1.016, 1.230]	[0.998, 1.209]
					s.e. = 0.055	s.e. = 0.054
					p = 0.023	p = 0.056
Education: Postgraduate Degree	Low				1.207	1.179
					[0.905, 1.610]	[0.880, 1.578]
					s.e. = 0.177	s.e. = 0.176

	response					
					p = 0.201	p = 0.270
	Medium				1.306	1.298
					[0.988, 1.727]	[0.981, 1.717]
					s.e. = 0.186	s.e. = 0.185
					p = 0.061	p = 0.067
Month: May	Low					1.933
						[1.710, 2.186]
						s.e. = 0.121
						p = <0.001
	Medium					1.289
						[1.142, 1.455]
						s.e. = 0.080
						p = <0.001
Month: June	Low					2.061
						[1.688, 2.516]
						s.e. = 0.210
						p = <0.001
	Medium					1.261
						[1.032, 1.542]
						s.e. = 0.129
						p = 0.024
Month: July	Low					2.228
						[1.898, 2.614]
						s.e. = 0.182
						p = <0.001
	Medium					1.463
						[1.248, 1.717]
						s.e. = 0.119
						p = <0.001
Month: August	Low					3.245
						[2.513, 4.189]
						s.e. = 0.423
						p = <0.001
	Medium					1.413
						[1.072, 1.861]
						s.e. = 0.199
						p = 0.014
Num.Obs.		11144	11144	11144	11144	11144
AIC		24350.1	24251.4	23776.7	23774.4	23535.5
Odds Ratios and 95% confidence intervals.						

Table 26 Step wise regression results for France, Outcome: Threat perception of COVID-19 to oneself

	response					
(Intercept)	Low	1.104	0.967	3.783	3.461	2.343
		[0.874, 1.395]	[0.764, 1.226]	[2.875, 4.979]	[2.593, 4.620]	[1.725, 3.182]
		s.e. = 0.132	s.e. = 0.117	s.e. = 0.530	s.e. = 0.510	s.e. = 0.366
		p = 0.405	p = 0.784	p = <0.001	p = <0.001	p = <0.001
	Medium	1.082	1.155	1.794	1.651	1.470
		[0.856, 1.369]	[0.912, 1.464]	[1.355, 2.374]	[1.231, 2.215]	[1.083, 1.995]
		s.e. = 0.130	s.e. = 0.140	s.e. = 0.257	s.e. = 0.248	s.e. = 0.229
		p = 0.510	p = 0.233	p = <0.001	p = <0.001	p = 0.014
Image: Couple blowing nose	Low	2.535	2.738	1.536	1.510	1.116
		[1.615, 3.981]	[1.742, 4.304]	[0.965, 2.445]	[0.948, 2.405]	[0.695, 1.792]
		s.e. = 0.584	s.e. = 0.632	s.e. = 0.364	s.e. = 0.359	s.e. = 0.270
		p = <0.001	p = <0.001	p = 0.071	p = 0.082	p = 0.651
	Medium	1.479	1.421	1.243	1.226	1.126
		[0.912, 2.398]	[0.876, 2.306]	[0.764, 2.022]	[0.753, 1.996]	[0.689, 1.840]
		s.e. = 0.365	s.e. = 0.351	s.e. = 0.309	s.e. = 0.305	s.e. = 0.282
		p = 0.113	p = 0.154	p = 0.382	p = 0.412	p = 0.636
image5 - Woman wearing mask	Low	1.211	1.213	0.917	0.902	0.969
		[0.953, 1.537]	[0.955, 1.542]	[0.719, 1.170]	[0.706, 1.151]	[0.756, 1.243]
		s.e. = 0.148	s.e. = 0.148	s.e. = 0.114	s.e. = 0.112	s.e. = 0.123
		p = 0.117	p = 0.114	p = 0.486	p = 0.406	p = 0.807
	Medium	1.081	1.079	1.020	1.005	1.004
		[0.850, 1.374]	[0.849, 1.373]	[0.801, 1.299]	[0.789, 1.281]	[0.787, 1.282]
		s.e. = 0.133	s.e. = 0.132	s.e. = 0.126	s.e. = 0.124	s.e. = 0.125
		p = 0.527	p = 0.534	p = 0.870	p = 0.967	p = 0.973

	response					
Image: Man wearing mask	Low	0.963	0.983	0.913	0.904	0.913
		[0.738, 1.257]	[0.753, 1.284]	[0.696, 1.198]	[0.689, 1.186]	[0.692, 1.203]
		s.e. = 0.131	s.e. = 0.134	s.e. = 0.127	s.e. = 0.125	s.e. = 0.129
		p = 0.781	p = 0.901	p = 0.512	p = 0.466	p = 0.517
	Medium	1.103	1.090	1.072	1.063	1.034
		[0.845, 1.438]	[0.836, 1.423]	[0.820, 1.400]	[0.813, 1.389]	[0.790, 1.353]
		s.e. = 0.150	s.e. = 0.148	s.e. = 0.146	s.e. = 0.145	s.e. = 0.142
		p = 0.472	p = 0.524	p = 0.611	p = 0.656	p = 0.809
Sex: Male	Low		1.476	1.630	1.641	1.609
			[1.339, 1.627]	[1.475, 1.801]	[1.484, 1.814]	[1.454, 1.781]
			s.e. = 0.073	s.e. = 0.083	s.e. = 0.084	s.e. = 0.083
			p = <0.001	p = <0.001	p = <0.001	p = <0.001
	Medium		0.787	0.804	0.806	0.802
			[0.709, 0.875]	[0.723, 0.893]	[0.725, 0.897]	[0.721, 0.892]
			s.e. = 0.042	s.e. = 0.043	s.e. = 0.044	s.e. = 0.043
			p = <0.001	p = <0.001	p = <0.001	p = <0.001
Age: 25- 44	Low			0.353	0.357	0.335
				[0.303, 0.412]	[0.306, 0.416]	[0.287, 0.392]
				s.e. = 0.028	s.e. = 0.028	s.e. = 0.027
				p = <0.001	p = <0.001	p = <0.001
	Medium			0.682	0.685	0.676
				[0.577, 0.807]	[0.579, 0.811]	[0.571, 0.800]
				s.e. = 0.059	s.e. = 0.059	s.e. = 0.058
				p = <0.001	p = <0.001	p = <0.001
Age: 45- 64	Low			0.205	0.211	0.191
				[0.176, 0.239]	[0.181, 0.247]	[0.163, 0.224]
				s.e. = 0.016	s.e. = 0.017	s.e. = 0.015
				p = <0.001	p = <0.001	p = <0.001
	Medium			0.619	0.632	0.621
				[0.525, 0.729]	[0.535, 0.746]	[0.526, 0.734]
				s.e. = 0.052	s.e. = 0.054	s.e. = 0.053
				p = <0.001	p = <0.001	p = <0.001
Age: 65+	Low			0.216	0.224	0.199
				[0.182, 0.257]	[0.188, 0.266]	[0.167, 0.237]
				s.e. = 0.019	s.e. = 0.020	s.e. = 0.018
				p = <0.001	p = <0.001	p = <0.001
	Medium			0.632	0.647	0.635
				[0.528, 0.756]	[0.539, 0.776]	[0.528, 0.762]
				s.e. = 0.058	s.e. = 0.060	s.e. = 0.059
				p = <0.001	p = <0.001	p = <0.001
Education: University level	Low				1.122	1.092
					[1.000, 1.258]	[0.972, 1.226]
					s.e. = 0.066	s.e. = 0.065
					p = 0.050	p = 0.137
	Medium				1.111	1.105
					[0.993, 1.244]	[0.987, 1.237]
					s.e. = 0.064	s.e. = 0.064
					p = 0.066	p = 0.084
Education: Postgraduate Degree	Low				1.037	0.961
					[0.846, 1.272]	[0.781, 1.182]
					s.e. = 0.108	s.e. = 0.101
					p = 0.724	p = 0.705
	Medium				1.133	1.115
					[0.929, 1.383]	[0.913, 1.361]
					s.e. = 0.115	s.e. = 0.113
					p = 0.217	p = 0.286
Month: April	Low					1.267
						[1.110, 1.446]
						s.e. = 0.085
						p = <0.001
	Medium					1.124
						[0.993, 1.272]
						s.e. = 0.071
						p = 0.065
Month: May	Low					2.039
						[1.752, 2.373]
						s.e. = 0.158
						p = <0.001
	Medium					1.307
						[1.127, 1.515]
						s.e. = 0.099

	response					
						p = <0.001
Month: June	Low					2.568
						[2.056, 3.208]
						s.e. = 0.291
						p = <0.001
	Medium					1.209
						[0.962, 1.519]
						s.e. = 0.141
						p = 0.104
Month: July	Low					2.158
						[1.806, 2.579]
						s.e. = 0.196
						p = <0.001
	Medium					1.363
						[1.143, 1.624]
						s.e. = 0.122
						p = <0.001
Month: August	Low					3.107
						[2.439, 3.958]
						s.e. = 0.384
						p = <0.001
	Medium					1.081
						[0.826, 1.413]
						s.e. = 0.148
						p = 0.571
Num.Obs.		11670	11670	11670	11670	11670
AIC		25483.3	25312.7	24721.0	24723.1	24498.6
Odds Ratios and 95% confidence intervals.						

Table 27 Step wise regression results for Germany, Outcome: Threat perception of COVID-19 to oneself

	response					
(Intercept)	Low	1.530	1.396	4.251	4.221	2.449
		[1.290, 1.814]	[1.176, 1.657]	[3.462, 5.219]	[3.437, 5.183]	[1.970, 3.046]
		s.e. = 0.133	s.e. = 0.122	s.e. = 0.445	s.e. = 0.442	s.e. = 0.272
		p = <0.001	p = <0.001	p = <0.001	p = <0.001	p = <0.001
	Medium	1.014	1.017	1.675	1.648	1.454
		[0.841, 1.222]	[0.843, 1.227]	[1.338, 2.096]	[1.316, 2.063]	[1.152, 1.836]
		s.e. = 0.097	s.e. = 0.097	s.e. = 0.192	s.e. = 0.189	s.e. = 0.173
		p = 0.886	p = 0.864	p = <0.001	p = <0.001	p = 0.002
Image: Couple blowing nose	Low	1.868	1.807	1.499	1.495	1.077
		[1.294, 2.697]	[1.251, 2.611]	[1.031, 2.179]	[1.028, 2.174]	[0.735, 1.578]
		s.e. = 0.350	s.e. = 0.339	s.e. = 0.286	s.e. = 0.286	s.e. = 0.210
		p = <0.001	p = 0.002	p = 0.034	p = 0.035	p = 0.703
	Medium	1.490	1.492	1.393	1.384	1.266
		[0.992, 2.237]	[0.993, 2.240]	[0.927, 2.095]	[0.920, 2.082]	[0.840, 1.909]
		s.e. = 0.309	s.e. = 0.309	s.e. = 0.290	s.e. = 0.288	s.e. = 0.265
		p = 0.055	p = 0.054	p = 0.111	p = 0.118	p = 0.260
image5 - Woman wearing mask	Low	2.006	1.926	1.332	1.323	1.293
		[1.684, 2.390]	[1.616, 2.295]	[1.112, 1.596]	[1.104, 1.585]	[1.075, 1.556]
		s.e. = 0.179	s.e. = 0.172	s.e. = 0.123	s.e. = 0.122	s.e. = 0.122
		p = <0.001	p = <0.001	p = 0.002	p = 0.002	p = 0.006
	Medium	1.520	1.522	1.292	1.273	1.242
		[1.255, 1.842]	[1.256, 1.845]	[1.062, 1.571]	[1.046, 1.548]	[1.019, 1.512]
		s.e. = 0.149	s.e. = 0.149	s.e. = 0.129	s.e. = 0.127	s.e. = 0.125
		p = <0.001	p = <0.001	p = 0.010	p = 0.016	p = 0.031
Image: Man wearing mask	Low	1.318	1.260	1.153	1.151	1.121
		[1.090, 1.593]	[1.042, 1.525]	[0.951, 1.399]	[0.949, 1.396]	[0.920, 1.366]
		s.e. = 0.128	s.e. = 0.122	s.e. = 0.114	s.e. = 0.113	s.e. = 0.113
		p = 0.004	p = 0.017	p = 0.147	p = 0.154	p = 0.257
	Medium	1.067	1.069	1.031	1.025	0.999
		[0.865, 1.316]	[0.867, 1.318]	[0.836, 1.273]	[0.831, 1.265]	[0.809, 1.235]
		s.e. = 0.114	s.e. = 0.114	s.e. = 0.111	s.e. = 0.110	s.e. = 0.108
		p = 0.544	p = 0.535	p = 0.773	p = 0.817	p = 0.995
Sex: Male	Low		1.391	1.630	1.622	1.598
			[1.294, 1.495]	[1.513, 1.756]	[1.506, 1.748]	[1.482, 1.723]
			s.e. = 0.051	s.e. = 0.062	s.e. = 0.062	s.e. = 0.061
			p = <0.001	p = <0.001	p = <0.001	p = <0.001

	response					
	Medium		0.988	1.055	1.048	1.044
			[0.911, 1.073]	[0.971, 1.146]	[0.964, 1.138]	[0.961, 1.135]
			s.e. = 0.041	s.e. = 0.045	s.e. = 0.044	s.e. = 0.044
			p = 0.780	p = 0.210	p = 0.273	p = 0.305
Age: 25- 44	Low			0.495	0.484	0.442
				[0.439, 0.558]	[0.429, 0.546]	[0.391, 0.499]
				s.e. = 0.030	s.e. = 0.030	s.e. = 0.028
				p = <0.001	p = <0.001	p = <0.001
	Medium			0.766	0.734	0.717
				[0.670, 0.876]	[0.641, 0.840]	[0.626, 0.821]
				s.e. = 0.052	s.e. = 0.051	s.e. = 0.050
				p = <0.001	p = <0.001	p = <0.001
Age: 45- 64	Low			0.314	0.309	0.266
				[0.278, 0.355]	[0.273, 0.350]	[0.235, 0.301]
				s.e. = 0.020	s.e. = 0.019	s.e. = 0.017
				p = <0.001	p = <0.001	p = <0.001
	Medium			0.602	0.585	0.565
				[0.525, 0.690]	[0.510, 0.671]	[0.492, 0.649]
				s.e. = 0.042	s.e. = 0.041	s.e. = 0.040
				p = <0.001	p = <0.001	p = <0.001
Age: 65+	Low			0.220	0.215	0.191
				[0.191, 0.253]	[0.187, 0.248]	[0.166, 0.220]
				s.e. = 0.016	s.e. = 0.015	s.e. = 0.014
				p = <0.001	p = <0.001	p = <0.001
	Medium			0.517	0.498	0.485
				[0.444, 0.603]	[0.427, 0.581]	[0.416, 0.567]
				s.e. = 0.040	s.e. = 0.039	s.e. = 0.038
				p = <0.001	p = <0.001	p = <0.001
Education: University level	Low				1.083	1.058
					[1.002, 1.171]	[0.978, 1.144]
					s.e. = 0.043	s.e. = 0.042
					p = 0.045	p = 0.162
	Medium				1.182	1.174
					[1.085, 1.287]	[1.078, 1.279]
					s.e. = 0.051	s.e. = 0.051
					p = <0.001	p = <0.001
Education: Postgraduate Degree	Low				1.220	1.214
					[0.993, 1.498]	[0.986, 1.495]
					s.e. = 0.128	s.e. = 0.129
					p = 0.059	p = 0.068
	Medium				1.271	1.272
					[1.015, 1.592]	[1.016, 1.594]
					s.e. = 0.146	s.e. = 0.146
					p = 0.037	p = 0.036
Month: April	Low					1.814
						[1.642, 2.004]
						s.e. = 0.092
						p = <0.001
	Medium					1.190
						[1.073, 1.320]
						s.e. = 0.063
						p = 0.001
Month: May	Low					2.828
						[2.516, 3.180]
						s.e. = 0.169
						p = <0.001
	Medium					1.348
						[1.189, 1.528]
						s.e. = 0.086
						p = <0.001
Month: June	Low					3.692
						[3.071, 4.437]
						s.e. = 0.347
						p = <0.001
	Medium					1.317
						[1.070, 1.621]
						s.e. = 0.140
						p = 0.009
Month: July	Low					3.347
						[2.820, 3.973]
						s.e. = 0.293

	response					
						p = <0.001
	Medium					1.394
						[1.153, 1.685]
						s.e. = 0.135
						p = <0.001
Month: August	Low					2.749
						[2.199, 3.437]
						s.e. = 0.313
						p = <0.001
	Medium					1.324
						[1.034, 1.694]
						s.e. = 0.167
						p = 0.026
Num.Obs.		22363	22363	22363	22363	22363
AIC		44890.8	44744.9	44082.1	44071.6	43517.4
Odds Ratios and 95% confidence intervals.						

Table 28 Step wise regression results for Italy, Outcome: Threat perception of COVID-19 to oneself

	response					
(Intercept)	Low	0.623	0.552	2.573	2.501	1.524
		[0.539, 0.721]	[0.476, 0.640]	[2.120, 3.123]	[2.059, 3.038]	[1.238, 1.875]
		s.e. = 0.046	s.e. = 0.042	s.e. = 0.254	s.e. = 0.248	s.e. = 0.161
		p = <0.001	p = <0.001	p = <0.001	p = <0.001	p = <0.001
	Medium	0.832	0.829	2.101	2.058	1.749
		[0.727, 0.951]	[0.724, 0.950]	[1.749, 2.525]	[1.711, 2.474]	[1.442, 2.123]
		s.e. = 0.057	s.e. = 0.057	s.e. = 0.197	s.e. = 0.193	s.e. = 0.173
		p = 0.007	p = 0.007	p = <0.001	p = <0.001	p = <0.001
Image: Couple blowing nose	Low	1.243	1.228	0.933	0.920	0.850
		[0.905, 1.707]	[0.893, 1.688]	[0.670, 1.299]	[0.660, 1.281]	[0.608, 1.190]
		s.e. = 0.201	s.e. = 0.199	s.e. = 0.158	s.e. = 0.155	s.e. = 0.146
		p = 0.179	p = 0.206	p = 0.681	p = 0.620	p = 0.344
	Medium	1.213	1.212	1.047	1.035	1.022
		[0.903, 1.629]	[0.903, 1.628]	[0.775, 1.413]	[0.767, 1.397]	[0.756, 1.381]
		s.e. = 0.182	s.e. = 0.182	s.e. = 0.160	s.e. = 0.158	s.e. = 0.157
		p = 0.199	p = 0.200	p = 0.766	p = 0.822	p = 0.890
image5 - Woman wearing mask	Low	1.382	1.336	0.946	0.939	1.216
		[1.186, 1.611]	[1.145, 1.558]	[0.805, 1.111]	[0.799, 1.103]	[1.029, 1.437]
		s.e. = 0.108	s.e. = 0.105	s.e. = 0.078	s.e. = 0.077	s.e. = 0.104
		p = <0.001	p = <0.001	p = 0.496	p = 0.444	p = 0.022
	Medium	1.360	1.358	1.111	1.103	1.202
		[1.181, 1.566]	[1.179, 1.564]	[0.962, 1.284]	[0.955, 1.275]	[1.036, 1.394]
		s.e. = 0.098	s.e. = 0.098	s.e. = 0.082	s.e. = 0.081	s.e. = 0.091
		p = <0.001	p = <0.001	p = 0.153	p = 0.182	p = 0.015
Image: Man wearing mask	Low	0.855	0.844	0.919	0.915	1.056
		[0.702, 1.041]	[0.693, 1.028]	[0.750, 1.128]	[0.746, 1.122]	[0.858, 1.301]
		s.e. = 0.086	s.e. = 0.085	s.e. = 0.096	s.e. = 0.095	s.e. = 0.112
		p = 0.118	p = 0.091	p = 0.421	p = 0.393	p = 0.607
	Medium	0.994	0.994	1.064	1.059	1.103
		[0.833, 1.187]	[0.833, 1.186]	[0.889, 1.274]	[0.885, 1.268]	[0.920, 1.322]
		s.e. = 0.090	s.e. = 0.090	s.e. = 0.098	s.e. = 0.097	s.e. = 0.102
		p = 0.948	p = 0.945	p = 0.498	p = 0.531	p = 0.289
Sex: Male	Low		1.534	1.746	1.759	1.880
			[1.405, 1.675]	[1.593, 1.914]	[1.604, 1.929]	[1.712, 2.064]
			s.e. = 0.069	s.e. = 0.082	s.e. = 0.083	s.e. = 0.090
			p = <0.001	p = <0.001	p = <0.001	p = <0.001
	Medium		1.014	1.094	1.101	1.125
			[0.932, 1.103]	[1.004, 1.192]	[1.010, 1.200]	[1.032, 1.226]
			s.e. = 0.044	s.e. = 0.048	s.e. = 0.048	s.e. = 0.050
			p = 0.754	p = 0.041	p = 0.029	p = 0.008
Age: 25- 44	Low			0.343	0.329	0.288
				[0.299, 0.393]	[0.286, 0.379]	[0.250, 0.332]
				s.e. = 0.024	s.e. = 0.024	s.e. = 0.021
				p = <0.001	p = <0.001	p = <0.001
	Medium			0.560	0.538	0.518
				[0.488, 0.642]	[0.468, 0.618]	[0.450, 0.596]
				s.e. = 0.039	s.e. = 0.038	s.e. = 0.037
				p = <0.001	p = <0.001	p = <0.001

	response					
Age: 45- 64	Low			0.149	0.146	0.121
				[0.129, 0.172]	[0.126, 0.169]	[0.104, 0.140]
				s.e. = 0.011	s.e. = 0.011	s.e. = 0.009
				p = <0.001	p = <0.001	p = <0.001
	Medium			0.345	0.336	0.320
				[0.300, 0.396]	[0.291, 0.386]	[0.277, 0.369]
				s.e. = 0.025	s.e. = 0.024	s.e. = 0.023
				p = <0.001	p = <0.001	p = <0.001
Age: 65+	Low			0.109	0.106	0.087
				[0.091, 0.130]	[0.088, 0.126]	[0.073, 0.105]
				s.e. = 0.010	s.e. = 0.010	s.e. = 0.008
				p = <0.001	p = <0.001	p = <0.001
	Medium			0.255	0.247	0.235
				[0.217, 0.300]	[0.210, 0.291]	[0.199, 0.277]
				s.e. = 0.021	s.e. = 0.021	s.e. = 0.020
				p = <0.001	p = <0.001	p = <0.001
Education: University level	Low				1.159	1.164
					[1.053, 1.275]	[1.057, 1.282]
					s.e. = 0.057	s.e. = 0.057
					p = 0.003	p = 0.002
	Medium				1.121	1.121
					[1.028, 1.223]	[1.028, 1.223]
					s.e. = 0.049	s.e. = 0.050
					p = 0.009	p = 0.010
Education: Postgraduate Degree	Low				1.079	1.094
					[0.924, 1.259]	[0.936, 1.280]
					s.e. = 0.085	s.e. = 0.087
					p = 0.337	p = 0.258
	Medium				1.150	1.155
					[1.005, 1.315]	[1.009, 1.322]
					s.e. = 0.079	s.e. = 0.080
					p = 0.042	p = 0.037
Month: April	Low					1.593
						[1.423, 1.784]
						s.e. = 0.092
						p = <0.001
	Medium					1.224
						[1.108, 1.353]
						s.e. = 0.062
						p = <0.001
Month: May	Low					2.601
						[2.294, 2.949]
						s.e. = 0.167
						p = <0.001
	Medium					1.362
						[1.212, 1.529]
						s.e. = 0.081
						p = <0.001
Month: June	Low					2.548
						[2.030, 3.197]
						s.e. = 0.295
						p = <0.001
	Medium					1.395
						[1.131, 1.720]
						s.e. = 0.149
						p = 0.002
Month: July	Low					2.016
						[1.657, 2.452]
						s.e. = 0.201
						p = <0.001
	Medium					1.092
						[0.907, 1.313]
						s.e. = 0.103
						p = 0.353
Month: August	Low					1.335
						[0.988, 1.804]
						s.e. = 0.205
						p = 0.060
	Medium					1.133
						[0.878, 1.464]
						s.e. = 0.148

	response					
						p = 0.337
Num.Obs.		14202	14202	14202	14202	14202
AIC		30944.1	30832.7	29818.8	29813.9	29555.5
Odds Ratios and 95% confidence intervals.						

Table 29 Step wise regression results for the Netherlands, Outcome: Threat perception of COVID-19 to oneself

	response					
(Intercept)	Low	0.930	0.824	3.380	3.314	3.025
		[0.824, 1.050]	[0.727, 0.935]	[2.634, 4.337]	[2.580, 4.257]	[2.344, 3.903]
		s.e. = 0.057	s.e. = 0.053	s.e. = 0.430	s.e. = 0.423	s.e. = 0.393
		p = 0.241	p = 0.003	p = <0.001	p = <0.001	p = <0.001
	Medium	0.862	0.888	1.845	1.809	1.805
		[0.762, 0.975]	[0.782, 1.009]	[1.414, 2.408]	[1.384, 2.363]	[1.380, 2.361]
		s.e. = 0.054	s.e. = 0.058	s.e. = 0.251	s.e. = 0.247	s.e. = 0.247
		p = 0.019	p = 0.068	p = <0.001	p = <0.001	p = <0.001
Image: Couple blowing nose	Low	1.695	1.672	1.289	1.285	0.875
		[1.197, 2.400]	[1.179, 2.370]	[0.902, 1.843]	[0.899, 1.836]	[0.603, 1.269]
		s.e. = 0.301	s.e. = 0.298	s.e. = 0.235	s.e. = 0.234	s.e. = 0.166
		p = 0.003	p = 0.004	p = 0.163	p = 0.170	p = 0.481
	Medium	1.337	1.342	1.206	1.202	1.142
		[0.923, 1.935]	[0.927, 1.943]	[0.831, 1.751]	[0.828, 1.744]	[0.785, 1.662]
		s.e. = 0.252	s.e. = 0.253	s.e. = 0.229	s.e. = 0.228	s.e. = 0.219
		p = 0.124	p = 0.119	p = 0.324	p = 0.334	p = 0.488
image5 - Woman wearing mask	Low	1.720	1.637	1.268	1.246	1.080
		[1.495, 1.980]	[1.421, 1.886]	[1.094, 1.470]	[1.074, 1.447]	[0.926, 1.261]
		s.e. = 0.123	s.e. = 0.118	s.e. = 0.095	s.e. = 0.095	s.e. = 0.085
		p = <0.001	p = <0.001	p = 0.002	p = 0.004	p = 0.327
	Medium	1.371	1.389	1.254	1.232	1.194
		[1.186, 1.584]	[1.201, 1.607]	[1.078, 1.458]	[1.057, 1.434]	[1.023, 1.393]
		s.e. = 0.101	s.e. = 0.103	s.e. = 0.096	s.e. = 0.096	s.e. = 0.094
		p = <0.001	p = <0.001	p = 0.003	p = 0.007	p = 0.025
Image: Man wearing mask	Low	1.243	1.231	1.184	1.176	1.016
		[1.053, 1.468]	[1.042, 1.454]	[1.000, 1.401]	[0.993, 1.393]	[0.852, 1.212]
		s.e. = 0.106	s.e. = 0.105	s.e. = 0.102	s.e. = 0.101	s.e. = 0.091
		p = 0.010	p = 0.015	p = 0.050	p = 0.060	p = 0.859
	Medium	1.339	1.343	1.322	1.314	1.287
		[1.132, 1.584]	[1.135, 1.589]	[1.117, 1.565]	[1.110, 1.556]	[1.084, 1.528]
		s.e. = 0.115	s.e. = 0.115	s.e. = 0.114	s.e. = 0.113	s.e. = 0.113
		p = <0.001	p = <0.001	p = 0.001	p = 0.002	p = 0.004
Sex: Male	Low		1.476	1.682	1.675	1.637
			[1.322, 1.649]	[1.500, 1.885]	[1.494, 1.878]	[1.456, 1.840]
			s.e. = 0.083	s.e. = 0.098	s.e. = 0.098	s.e. = 0.098
			p = <0.001	p = <0.001	p = <0.001	p = <0.001
	Medium		0.892	0.936	0.932	0.931
			[0.792, 1.004]	[0.829, 1.056]	[0.826, 1.051]	[0.825, 1.051]
			s.e. = 0.054	s.e. = 0.058	s.e. = 0.057	s.e. = 0.058
			p = 0.059	p = 0.281	p = 0.251	p = 0.247
Age: 25- 44	Low			0.345	0.341	0.292
				[0.271, 0.439]	[0.268, 0.435]	[0.228, 0.373]
				s.e. = 0.043	s.e. = 0.042	s.e. = 0.037
				p = <0.001	p = <0.001	p = <0.001
	Medium			0.577	0.570	0.557
				[0.444, 0.749]	[0.438, 0.740]	[0.429, 0.725]
				s.e. = 0.077	s.e. = 0.076	s.e. = 0.075
				p = <0.001	p = <0.001	p = <0.001
Age: 45- 64	Low			0.213	0.215	0.164
				[0.169, 0.268]	[0.171, 0.271]	[0.130, 0.208]
				s.e. = 0.025	s.e. = 0.025	s.e. = 0.020
				p = <0.001	p = <0.001	p = <0.001
	Medium			0.447	0.452	0.432

	response					
				[0.349, 0.572]	[0.353, 0.578]	[0.337, 0.555]
				s.e. = 0.056	s.e. = 0.057	s.e. = 0.055
				p = <0.001	p = <0.001	p = <0.001
Age: 65+	Low			0.214	0.216	0.163
				[0.168, 0.272]	[0.170, 0.275]	[0.127, 0.209]
				s.e. = 0.026	s.e. = 0.027	s.e. = 0.021
				p = <0.001	p = <0.001	p = <0.001
	Medium			0.472	0.478	0.455
				[0.365, 0.610]	[0.369, 0.618]	[0.351, 0.590]
				s.e. = 0.062	s.e. = 0.063	s.e. = 0.060
				p = <0.001	p = <0.001	p = <0.001
Education: University level	Low				1.122	1.014
					[0.982, 1.283]	[0.884, 1.163]
					s.e. = 0.077	s.e. = 0.071
					p = 0.092	p = 0.842
	Medium				1.123	1.098
					[0.977, 1.291]	[0.954, 1.263]
					s.e. = 0.080	s.e. = 0.078
					p = 0.102	p = 0.191
Education: Postgraduate Degree	Low				0.993	0.835
					[0.658, 1.497]	[0.548, 1.273]
					s.e. = 0.208	s.e. = 0.180
					p = 0.972	p = 0.403
	Medium				1.013	0.975
					[0.658, 1.560]	[0.633, 1.504]
					s.e. = 0.223	s.e. = 0.215
					p = 0.953	p = 0.910
Month: May	Low					2.311
						[2.017, 2.649]
						s.e. = 0.161
						p = <0.001
	Medium					1.138
						[0.989, 1.310]
						s.e. = 0.081
						p = 0.070
Month: June	Low					4.475
						[3.507, 5.711]
						s.e. = 0.557
						p = <0.001
	Medium					1.379
						[1.048, 1.815]
						s.e. = 0.193
						p = 0.022
Month: July	Low					3.770
						[3.065, 4.637]
						s.e. = 0.398
						p = <0.001
	Medium					1.301
						[1.034, 1.636]
						s.e. = 0.152
						p = 0.025
Month: August	Low					2.664
						[1.921, 3.693]
						s.e. = 0.444
						p = <0.001
	Medium					1.372
						[0.967, 1.946]
						s.e. = 0.245
						p = 0.076
Num.Obs.		8223	8223	8223	8223	8223
AIC		17880.2	17789.1	17552.3	17556.7	17130.3
Odds Ratios and 95% confidence intervals.						

Table 30 Step wise regression results for Spain, Outcome: Threat perception of COVID-19 to oneself

	response					
(Intercept)	Low	0.759	0.736	2.522	2.743	2.650
		[0.656, 0.877]	[0.634, 0.853]	[1.951, 3.261]	[2.107, 3.570]	[2.003, 3.507]

	response					
		s.e. = 0.056	s.e. = 0.056	s.e. = 0.331	s.e. = 0.369	s.e. = 0.379
		p = <0.001	p = <0.001	p = <0.001	p = <0.001	p = <0.001
	Medium	0.920	0.946	1.592	1.583	1.616
		[0.801, 1.055]	[0.823, 1.089]	[1.220, 2.078]	[1.206, 2.080]	[1.214, 2.152]
		s.e. = 0.065	s.e. = 0.068	s.e. = 0.216	s.e. = 0.220	s.e. = 0.236
		p = 0.233	p = 0.440	p = <0.001	p = <0.001	p = 0.001
Image: Couple blowing nose	Low	1.156	1.149	1.026	1.029	0.962
		[0.845, 1.583]	[0.839, 1.572]	[0.747, 1.410]	[0.748, 1.414]	[0.698, 1.326]
		s.e. = 0.185	s.e. = 0.184	s.e. = 0.166	s.e. = 0.167	s.e. = 0.157
		p = 0.365	p = 0.388	p = 0.874	p = 0.862	p = 0.813
	Medium	1.026	1.032	0.990	0.989	0.976
		[0.755, 1.393]	[0.760, 1.402]	[0.728, 1.346]	[0.727, 1.344]	[0.717, 1.329]
		s.e. = 0.160	s.e. = 0.161	s.e. = 0.155	s.e. = 0.155	s.e. = 0.154
		p = 0.870	p = 0.839	p = 0.948	p = 0.943	p = 0.879
image5 - Woman wearing mask	Low	1.131	1.126	0.988	1.006	1.041
		[0.967, 1.322]	[0.963, 1.316]	[0.842, 1.160]	[0.857, 1.181]	[0.886, 1.224]
		s.e. = 0.090	s.e. = 0.090	s.e. = 0.081	s.e. = 0.082	s.e. = 0.086
		p = 0.123	p = 0.138	p = 0.884	p = 0.938	p = 0.623
	Medium	1.095	1.100	1.035	1.037	1.036
		[0.944, 1.271]	[0.948, 1.276]	[0.890, 1.203]	[0.891, 1.206]	[0.889, 1.206]
		s.e. = 0.083	s.e. = 0.083	s.e. = 0.080	s.e. = 0.080	s.e. = 0.081
		p = 0.230	p = 0.208	p = 0.659	p = 0.640	p = 0.652
Image: Man wearing mask	Low	0.913	0.908	0.918	0.927	0.942
		[0.766, 1.088]	[0.762, 1.083]	[0.769, 1.096]	[0.776, 1.107]	[0.788, 1.127]
		s.e. = 0.082	s.e. = 0.082	s.e. = 0.083	s.e. = 0.084	s.e. = 0.086
		p = 0.309	p = 0.283	p = 0.343	p = 0.400	p = 0.515
	Medium	0.998	1.003	1.012	1.013	1.017
		[0.846, 1.178]	[0.850, 1.183]	[0.857, 1.194]	[0.858, 1.196]	[0.860, 1.201]
		s.e. = 0.084	s.e. = 0.085	s.e. = 0.086	s.e. = 0.086	s.e. = 0.087
		p = 0.982	p = 0.973	p = 0.890	p = 0.876	p = 0.846
Sex: Male	Low		1.114	1.227	1.217	1.233
			[1.009, 1.231]	[1.108, 1.360]	[1.098, 1.349]	[1.113, 1.367]
			s.e. = 0.057	s.e. = 0.064	s.e. = 0.064	s.e. = 0.065
			p = 0.033	p = <0.001	p = <0.001	p = <0.001
	Medium		0.899	0.941	0.944	0.946
			[0.816, 0.990]	[0.853, 1.039]	[0.855, 1.042]	[0.857, 1.044]
			s.e. = 0.044	s.e. = 0.047	s.e. = 0.048	s.e. = 0.048
			p = 0.030	p = 0.230	p = 0.253	p = 0.271
Age: 25- 44	Low			0.367	0.374	0.363
				[0.293, 0.460]	[0.298, 0.468]	[0.289, 0.456]
				s.e. = 0.042	s.e. = 0.043	s.e. = 0.042
				p = <0.001	p = <0.001	p = <0.001
	Medium			0.677	0.685	0.680
				[0.533, 0.862]	[0.538, 0.871]	[0.534, 0.866]
				s.e. = 0.083	s.e. = 0.084	s.e. = 0.084
				p = 0.002	p = 0.002	p = 0.002
Age: 45- 64	Low			0.244	0.245	0.236
				[0.195, 0.305]	[0.195, 0.306]	[0.188, 0.295]
				s.e. = 0.028	s.e. = 0.028	s.e. = 0.027
				p = <0.001	p = <0.001	p = <0.001
	Medium			0.570	0.575	0.570
				[0.449, 0.723]	[0.453, 0.729]	[0.449, 0.724]
				s.e. = 0.069	s.e. = 0.070	s.e. = 0.070
				p = <0.001	p = <0.001	p = <0.001
Age: 65+	Low			0.257	0.263	0.251
				[0.201, 0.329]	[0.206, 0.337]	[0.196, 0.321]
				s.e. = 0.032	s.e. = 0.033	s.e. = 0.032
				p = <0.001	p = <0.001	p = <0.001
	Medium			0.524	0.532	0.526
				[0.405, 0.677]	[0.411, 0.688]	[0.406, 0.681]
				s.e. = 0.069	s.e. = 0.070	s.e. = 0.069
				p = <0.001	p = <0.001	p = <0.001
Education: University level	Low				0.869	0.872
					[0.784, 0.964]	[0.786, 0.967]
					s.e. = 0.046	s.e. = 0.046
					p = 0.008	p = 0.010
	Medium				1.015	1.015
					[0.920, 1.120]	[0.920, 1.120]
					s.e. = 0.051	s.e. = 0.051
					p = 0.768	p = 0.770
Education: Postgraduate Degree	Low				0.759	0.754

	response					
					[0.640, 0.901]	[0.635, 0.895]
					s.e. = 0.066	s.e. = 0.066
					p = 0.002	p = 0.001
	Medium				0.861	0.856
					[0.732, 1.011]	[0.728, 1.006]
					s.e. = 0.071	s.e. = 0.070
					p = 0.068	p = 0.059
Month: April	Low					0.916
						[0.801, 1.049]
						s.e. = 0.063
						p = 0.204
	Medium					0.966
						[0.851, 1.096]
						s.e. = 0.062
						p = 0.588
Month: May	Low					1.142
						[0.975, 1.337]
						s.e. = 0.092
						p = 0.099
	Medium					0.951
						[0.818, 1.106]
						s.e. = 0.073
						p = 0.517
Month: June	Low					1.342
						[1.062, 1.697]
						s.e. = 0.161
						p = 0.014
	Medium					1.018
						[0.810, 1.279]
						s.e. = 0.119
						p = 0.880
Month: July	Low					1.551
						[1.241, 1.940]
						s.e. = 0.177
						p = <0.001
	Medium					1.188
						[0.955, 1.478]
						s.e. = 0.132
						p = 0.121
Month: August	Low					1.540
						[1.109, 2.138]
						s.e. = 0.258
						p = 0.010
	Medium					1.197
						[0.866, 1.655]
						s.e. = 0.198
						p = 0.277
Num.Obs.		10964	10964	10964	10964	10964
AIC		23996.9	23983.5	23792.8	23783.7	23752.6
Odds Ratios and 95% confidence intervals.						

Table 31 Step wise regression results for the United Kingdom, Outcome: Threat perception of COVID-19 to oneself

	response					
(Intercept)	Low	0.458	0.398	2.429	2.291	1.642
		[0.384, 0.545]	[0.333, 0.475]	[1.845, 3.198]	[1.736, 3.025]	[1.232, 2.188]
		s.e. = 0.041	s.e. = 0.036	s.e. = 0.341	s.e. = 0.325	s.e. = 0.241
		p = <0.001	p = <0.001	p = <0.001	p = <0.001	p = <0.001
	Medium	0.721	0.713	1.533	1.466	1.332
		[0.620, 0.839]	[0.612, 0.831]	[1.168, 2.012]	[1.114, 1.928]	[1.008, 1.760]
		s.e. = 0.056	s.e. = 0.056	s.e. = 0.213	s.e. = 0.205	s.e. = 0.189
		p = <0.001	p = <0.001	p = 0.002	p = 0.006	p = 0.044
Image: Couple blowing nose	Low	2.554	2.509	2.028	2.011	1.649
		[1.798, 3.626]	[1.764, 3.567]	[1.415, 2.909]	[1.402, 2.884]	[1.139, 2.388]
		s.e. = 0.457	s.e. = 0.451	s.e. = 0.373	s.e. = 0.370	s.e. = 0.311
		p = <0.001	p = <0.001	p = <0.001	p = <0.001	p = 0.008
	Medium	1.656	1.654	1.553	1.539	1.465
		[1.181, 2.323]	[1.179, 2.319]	[1.106, 2.181]	[1.096, 2.162]	[1.042, 2.061]

	response					
		s.e. = 0.286	s.e. = 0.285	s.e. = 0.269	s.e. = 0.267	s.e. = 0.255
		p = 0.003	p = 0.004	p = 0.011	p = 0.013	p = 0.028
image5 - Woman wearing mask	Low	1.404	1.359	1.114	1.086	1.264
		[1.170, 1.685]	[1.132, 1.633]	[0.924, 1.343]	[0.900, 1.310]	[1.043, 1.533]
		s.e. = 0.131	s.e. = 0.127	s.e. = 0.106	s.e. = 0.104	s.e. = 0.124
		p = <0.001	p = 0.001	p = 0.258	p = 0.391	p = 0.017
	Medium	1.250	1.246	1.183	1.155	1.206
		[1.066, 1.465]	[1.063, 1.461]	[1.008, 1.388]	[0.983, 1.356]	[1.025, 1.419]
		s.e. = 0.101	s.e. = 0.101	s.e. = 0.097	s.e. = 0.095	s.e. = 0.100
		p = 0.006	p = 0.007	p = 0.040	p = 0.080	p = 0.024
Image: Man wearing mask	Low	1.076	1.023	1.012	0.995	1.040
		[0.867, 1.335]	[0.824, 1.271]	[0.812, 1.260]	[0.798, 1.240]	[0.831, 1.301]
		s.e. = 0.118	s.e. = 0.113	s.e. = 0.113	s.e. = 0.112	s.e. = 0.119
		p = 0.507	p = 0.836	p = 0.916	p = 0.964	p = 0.733
	Medium	1.068	1.063	1.056	1.039	1.044
		[0.886, 1.288]	[0.882, 1.282]	[0.875, 1.274]	[0.860, 1.254]	[0.864, 1.261]
		s.e. = 0.102	s.e. = 0.102	s.e. = 0.101	s.e. = 0.100	s.e. = 0.101
		p = 0.491	p = 0.521	p = 0.568	p = 0.693	p = 0.657
Sex: Male	Low		1.586	1.723	1.722	1.734
			[1.439, 1.748]	[1.559, 1.905]	[1.558, 1.904]	[1.566, 1.920]
			s.e. = 0.079	s.e. = 0.088	s.e. = 0.088	s.e. = 0.090
			p = <0.001	p = <0.001	p = <0.001	p = <0.001
	Medium		1.044	1.069	1.067	1.071
			[0.954, 1.143]	[0.976, 1.171]	[0.974, 1.168]	[0.978, 1.174]
			s.e. = 0.048	s.e. = 0.050	s.e. = 0.050	s.e. = 0.050
			p = 0.353	p = 0.153	p = 0.166	p = 0.140
Age: 25- 44	Low			0.285	0.276	0.254
				[0.227, 0.358]	[0.219, 0.347]	[0.201, 0.320]
				s.e. = 0.033	s.e. = 0.032	s.e. = 0.030
				p = <0.001	p = <0.001	p = <0.001
	Medium			0.577	0.558	0.545
				[0.453, 0.735]	[0.438, 0.712]	[0.428, 0.696]
				s.e. = 0.071	s.e. = 0.069	s.e. = 0.068
				p = <0.001	p = <0.001	p = <0.001
Age: 45- 64	Low			0.134	0.134	0.110
				[0.108, 0.167]	[0.107, 0.167]	[0.088, 0.138]
				s.e. = 0.015	s.e. = 0.015	s.e. = 0.013
				p = <0.001	p = <0.001	p = <0.001
	Medium			0.429	0.426	0.404
				[0.340, 0.542]	[0.338, 0.538]	[0.320, 0.510]
				s.e. = 0.051	s.e. = 0.051	s.e. = 0.048
				p = <0.001	p = <0.001	p = <0.001
Age: 65+	Low			0.140	0.141	0.112
				[0.112, 0.176]	[0.112, 0.177]	[0.088, 0.141]
				s.e. = 0.016	s.e. = 0.016	s.e. = 0.013
				p = <0.001	p = <0.001	p = <0.001
	Medium			0.460	0.462	0.433
				[0.363, 0.584]	[0.364, 0.586]	[0.341, 0.550]
				s.e. = 0.056	s.e. = 0.056	s.e. = 0.053
				p = <0.001	p = <0.001	p = <0.001
Education: University level	Low				1.172	1.148
					[1.057, 1.298]	[1.034, 1.274]
					s.e. = 0.061	s.e. = 0.061
					p = 0.002	p = 0.009
	Medium				1.136	1.133
					[1.038, 1.244]	[1.035, 1.240]
					s.e. = 0.052	s.e. = 0.052
					p = 0.005	p = 0.007
Education: Postgraduate Degree	Low				1.201	1.184
					[0.993, 1.452]	[0.976, 1.436]
					s.e. = 0.116	s.e. = 0.117
					p = 0.058	p = 0.087
	Medium				1.237	1.239
					[1.047, 1.462]	[1.048, 1.464]
					s.e. = 0.105	s.e. = 0.106
					p = 0.012	p = 0.012
Month: April	Low					1.264
						[1.110, 1.440]
						s.e. = 0.084
						p = <0.001
	Medium					1.094

	response					
						[0.982, 1.218]
						s.e. = 0.060
						p = 0.104
Month: May	Low					2.298
						[2.000, 2.641]
						s.e. = 0.163
						p = <0.001
	Medium					1.337
						[1.180, 1.514]
						s.e. = 0.085
						p = <0.001
Month: June	Low					2.767
						[2.210, 3.466]
						s.e. = 0.318
						p = <0.001
	Medium					1.327
						[1.071, 1.642]
						s.e. = 0.145
						p = 0.010
Month: July	Low					3.283
						[2.694, 4.000]
						s.e. = 0.331
						p = <0.001
	Medium					1.411
						[1.164, 1.711]
						s.e. = 0.139
						p = <0.001
Month: August	Low					4.422
						[3.166, 6.177]
						s.e. = 0.754
						p = <0.001
	Medium					1.652
						[1.175, 2.322]
						s.e. = 0.287
						p = 0.004
Num.Obs.		11401	11401	11401	11401	11401
AIC		24571.4	24477.9	23996.9	23989.7	23695.2
Odds Ratios and 95% confidence intervals.						

Table 32 Step wise regression results for the United States, Outcome: Threat perception of COVID-19 to oneself

	response					
(Intercept)	Low	1.591	1.222	3.143	3.562	2.298
		[1.350, 1.874]	[1.034, 1.445]	[2.564, 3.851]	[2.900, 4.374]	[1.856, 2.844]
		s.e. = 0.133	s.e. = 0.104	s.e. = 0.326	s.e. = 0.373	s.e. = 0.250
		p = <0.001	p = 0.019	p = <0.001	p = <0.001	p = <0.001
	Medium	1.030	1.022	1.716	1.686	1.601
		[0.860, 1.234]	[0.853, 1.226]	[1.376, 2.139]	[1.349, 2.106]	[1.274, 2.012]
		s.e. = 0.095	s.e. = 0.095	s.e. = 0.193	s.e. = 0.192	s.e. = 0.187
		p = 0.747	p = 0.811	p = <0.001	p = <0.001	p = <0.001
Image: Couple blowing nose	Low	0.952	0.923	0.815	0.822	0.808
		[0.710, 1.275]	[0.686, 1.241]	[0.605, 1.099]	[0.609, 1.109]	[0.598, 1.092]
		s.e. = 0.142	s.e. = 0.140	s.e. = 0.124	s.e. = 0.126	s.e. = 0.124
		p = 0.740	p = 0.596	p = 0.181	p = 0.200	p = 0.165
	Medium	1.078	1.076	0.986	0.987	0.978
		[0.786, 1.478]	[0.785, 1.476]	[0.718, 1.353]	[0.719, 1.356]	[0.712, 1.343]
		s.e. = 0.174	s.e. = 0.173	s.e. = 0.159	s.e. = 0.160	s.e. = 0.158
		p = 0.643	p = 0.647	p = 0.929	p = 0.938	p = 0.892
image5 - Woman wearing mask	Low	0.937	0.887	0.819	0.825	0.973
		[0.793, 1.107]	[0.749, 1.051]	[0.691, 0.972]	[0.695, 0.979]	[0.817, 1.158]
		s.e. = 0.080	s.e. = 0.077	s.e. = 0.071	s.e. = 0.072	s.e. = 0.087
		p = 0.445	p = 0.166	p = 0.022	p = 0.028	p = 0.757
	Medium	1.033	1.031	0.966	0.967	0.985
		[0.860, 1.241]	[0.858, 1.239]	[0.804, 1.162]	[0.804, 1.163]	[0.817, 1.188]
		s.e. = 0.097	s.e. = 0.097	s.e. = 0.091	s.e. = 0.091	s.e. = 0.094
		p = 0.729	p = 0.744	p = 0.717	p = 0.723	p = 0.876
Image: Man wearing mask	Low	0.789	0.737	0.747	0.753	0.825
		[0.658, 0.948]	[0.613, 0.888]	[0.620, 0.900]	[0.625, 0.908]	[0.682, 0.997]

	response					
		s.e. = 0.074	s.e. = 0.070	s.e. = 0.071	s.e. = 0.072	s.e. = 0.080
		p = 0.011	p = 0.001	p = 0.002	p = 0.003	p = 0.046
	Medium	1.020	1.018	1.031	1.032	1.039
		[0.836, 1.245]	[0.834, 1.242]	[0.845, 1.259]	[0.846, 1.260]	[0.850, 1.271]
		s.e. = 0.104	s.e. = 0.103	s.e. = 0.105	s.e. = 0.105	s.e. = 0.107
		p = 0.844	p = 0.862	p = 0.761	p = 0.754	p = 0.709
Sex: Male	Low		2.369	2.515	2.483	2.475
			[2.229, 2.518]	[2.364, 2.676]	[2.333, 2.642]	[2.324, 2.636]
			s.e. = 0.074	s.e. = 0.079	s.e. = 0.079	s.e. = 0.079
			p = <0.001	p = <0.001	p = <0.001	p = <0.001
	Medium		1.034	1.084	1.087	1.091
			[0.966, 1.107]	[1.012, 1.161]	[1.015, 1.165]	[1.018, 1.169]
			s.e. = 0.036	s.e. = 0.038	s.e. = 0.038	s.e. = 0.038
			p = 0.329	p = 0.021	p = 0.017	p = 0.014
Age: 25- 44	Low			0.450	0.497	0.456
				[0.395, 0.513]	[0.436, 0.567]	[0.399, 0.520]
				s.e. = 0.030	s.e. = 0.033	s.e. = 0.031
				p = <0.001	p = <0.001	p = <0.001
	Medium			0.744	0.739	0.730
				[0.647, 0.856]	[0.641, 0.851]	[0.634, 0.842]
				s.e. = 0.053	s.e. = 0.053	s.e. = 0.053
				p = <0.001	p = <0.001	p = <0.001
Age: 45- 64	Low			0.390	0.419	0.360
				[0.344, 0.442]	[0.370, 0.475]	[0.317, 0.409]
				s.e. = 0.025	s.e. = 0.027	s.e. = 0.023
				p = <0.001	p = <0.001	p = <0.001
	Medium			0.604	0.601	0.590
				[0.528, 0.692]	[0.525, 0.689]	[0.514, 0.677]
				s.e. = 0.042	s.e. = 0.042	s.e. = 0.041
				p = <0.001	p = <0.001	p = <0.001
Age: 65+	Low			0.323	0.354	0.300
				[0.285, 0.367]	[0.312, 0.402]	[0.263, 0.341]
				s.e. = 0.021	s.e. = 0.023	s.e. = 0.020
				p = <0.001	p = <0.001	p = <0.001
	Medium			0.510	0.507	0.497
				[0.444, 0.585]	[0.441, 0.582]	[0.432, 0.572]
				s.e. = 0.036	s.e. = 0.036	s.e. = 0.035
				p = <0.001	p = <0.001	p = <0.001
Education: University level	Low				0.734	0.727
					[0.690, 0.780]	[0.683, 0.773]
					s.e. = 0.023	s.e. = 0.023
					p = <0.001	p = <0.001
	Medium				1.037	1.035
					[0.971, 1.108]	[0.969, 1.105]
					s.e. = 0.035	s.e. = 0.035
					p = 0.283	p = 0.311
Education: Postgraduate Degree	Low				0.518	0.518
					[0.453, 0.593]	[0.453, 0.594]
					s.e. = 0.036	s.e. = 0.036
					p = <0.001	p = <0.001
	Medium				1.001	0.997
					[0.877, 1.143]	[0.874, 1.138]
					s.e. = 0.067	s.e. = 0.067
					p = 0.987	p = 0.969
Month: April	Low					1.312
						[1.212, 1.420]
						s.e. = 0.053
						p = <0.001
	Medium					1.047
						[0.968, 1.132]
						s.e. = 0.042
						p = 0.250
Month: May	Low					2.358
						[2.165, 2.569]
						s.e. = 0.103
						p = <0.001
	Medium					1.124
						[1.027, 1.229]
						s.e. = 0.051
						p = 0.011
Month: June	Low					2.370

	response					
						[2.035, 2.760]
						s.e. = 0.184
						p = <0.001
	Medium					1.224
						[1.041, 1.440]
						s.e. = 0.101
						p = 0.015
Month: July	Low					1.824
						[1.630, 2.043]
						s.e. = 0.105
						p = <0.001
	Medium					1.060
						[0.941, 1.195]
						s.e. = 0.065
						p = 0.339
Month: August	Low					1.952
						[1.606, 2.373]
						s.e. = 0.194
						p = <0.001
	Medium					0.882
						[0.705, 1.105]
						s.e. = 0.101
						p = 0.276
Num.Obs.		28400	28400	28400	28400	28400
AIC		61559.1	60434.6	60057.8	59838.3	59240.2
Odds Ratios and 95% confidence intervals.						
