# Supplementary material for: Assessing self-selection biases in Facebook-recruited online surveys: Evidence from the COVID-19 Health Behavior Survey
Source: PLoS One. 2025 Jul 8;20(7):e0326884. doi: 10.1371/journal.pone.0326884 (PMC12237053; doi:10.1371/journal.pone.0326884)
Supplement: Attachment — Submitted filename: PONE-D-24-35721_review.pdf [file pone.0326884.s006.pdf]

PONE-D-24-35721
Assessing self-selection biases in online surveys: Evidence from the COVID-19 Health Behavior Survey
Reviewer comments

Summary

Overall, I thought the manuscript focus and hypotheses were quite interesting with regards to leverage-salience theory applications to COVID-19. This topic seems quite relevant, given the need to better understand disease outcomes and population perceptions. From a constructively critical standpoint, I did find the manuscript results a bit hard to follow. I think the authors could attempt to lay out the results more succinctly. I also think the visualization of the results could be more effectively presented. In your appendix 1, your S1 figure (number of participants by ad image and country over time) is a great depiction of the data and might be a nice addition to the actual manuscript (potentially at the beginning of your results?).

I would similarly think about how to better represent model significance. As a reader, I would really like to be able to capture that information visually, or in some way that allows me easily to see how some factors (i.e. month of survey participation) were impactful on model outcomes.

Introduction

Well structured. However, near the end of your introduction, you start to discuss the results of your analysis. I might remove that, and instead, formulate some questions that relate to your hypotheses. Do advertising images have a variable effect on survey outcomes? What factors (location, timeframe, population demographics, etc) might influence survey outcomes, given the heterogenous nature of leverage and salience?

I might also broaden the hypothesis to say that there are several influential factors which may determine who participates in the survey – which includes time frame of the pandemic – but also includes many other factors, and your intent is to tease out these interactions and effects based on the multi-level structure you have constructed (age/sex/location etc.).

Methods

I can appreciate the detail in the methodology, which is well organized. I may be a little fixated on visualizing outcomes, but you might consider one diagram which depicts the flow of your methodology – even if its in a supplemental appendix. Your approach focuses on survey data from this 2020 time frame, based on 6 differing ads which have some form of increasing COVID-19 prominence. Then you analyze based on several predictor factors. That could be presented in one diagram – so the reader would immediately be able to grasp the approach and how the models are applied.

Also – you mention a total of 32 models, I'm assuming based on the changing factor levels. If that is clearly defined, maybe I just missed it – but if not, id include that. Potentially in the diagram that I'm suggesting above.

Results

Page 9: "Fig 2 shows the variation in the levels of perceived threat towards COVID-19 that respondents reported for themselves and for their family, across the various countries and advertisement images. Respondents generally perceived COVID-19 as a higher threat to their family than to themselves."

I couldn't tell from Figure 2 that respondents perceived COVID-19 as a higher threat to their family than themselves? Am I missing something?

Also – if your models are binomial and multinomial logistic regressions, then a prominent summary statistic would be the odds ratios. I see you are reporting these in your S2 appendix. Maybe summarizing the odds ratios for the most influential models might be interesting to report in the manuscript? Again, if I missed that in the manuscript, my apologies. You could also present a standardized coefficient diagram that indicates those factors which are significant, with their odds ratios/Cis. Maybe that's already in the supplementals. If so – one of those diagrams in the results would seem to be very useful.

Discussion

I think your discussion would benefit by giving some thought as to WHY you think some of this variability exists – either across location or the differing interactions of influential factors on each of the images. My sense is – some of this language is more appropriate for the results section.

For example: you report some of the locational differences (based on country). Were their known policy or covid-19 spread variations based on county – that might be associated with what your results are indicating? I realize that's not the focus of this work – but I'm interested in why we are seeing these results, and what implications we might be infer?
