# Supplementary material for: Assessing self-selection biases in Facebook-recruited online surveys: Evidence from the COVID-19 Health Behavior Survey
Source: PLoS One. 2025 Jul 8;20(7):e0326884. doi: 10.1371/journal.pone.0326884 (PMC12237053; doi:10.1371/journal.pone.0326884)
Supplement: Attachment — Submitted filename: Comments to authors.docx [file pone.0326884.s005.docx]

Is the goal of Facebook-based sampling to obtain a representative sample of country’s population or representative sample of Facebook users?

Are the images validated measures of salience? While images 1-2 are fairly generic, it is not clear to me that images 3, 4, and 6 are any different in their level or intensity of COVID-19 salience (image 5 would be the most COVID-19 salient).

Lines 84-89: Are there potential cultural differences in perceptions or interpretations of images?

Lines 162-165: what exactly is meant by “interest” in the topic of COVID-19? The term “interest” is too generic. Can authors be more specific here? Do you mean to say concern of infection varied by demographics? Risk of COVID-19 severity varied by demographics?

Lines 216-219: highly recommend including a flow chart showing n(%) excluded for each reason (similar to a CONSORT or STROBE diagram). Given over half of respondents were dropped from analysis, it is unclear how representative the resulting analytic sample really is.

Table S3: Reach and click %s are quite low—6% (1,799,691/30,140,838) of those reached clicked through. Can authors explain whether such low reach and clicks are common? It is difficult to get a sense on how poor or acceptable this level of click through is in terms of recruitment and participation.

Figure 3: suggests to me that image did in fact result in selection bias since adoption rates did vary by image. On lines 306-309, it says “the level of compliance with hand hygiene recommendations did not differ significantly across images…with the exception of the group of athletes image.” Doesn’t this in fact indicate that there is selection bias? One would have expected there to be NO variation at all.

Figure 4: what is the meaning of the “Low” “Medium” and “High” strata at the top? Please clarify in image.

Month of survey has a profound effect on results; have you tested whether there is interaction between image and month pandemic? It may be that the pandemic month modifies the effect the images have on respondent’s threat perception and behaviors since pandemic month is a proxy for actual risk (i.e., COVID-19 transmission and deaths increased and decreased during the pandemic). The effect of images may differ when actual risk is high (during a peak in cases/deaths) vs when it is low (during a lull in cases/deaths).

Unclear how these findings should be applied to future research. Does it mean that investigators can use salient images to maximize recruitment without fear of inducing selection bias?

Lines 519-521: can authors further expand on what they mean by “capturing trends within the general online population”? It is unclear to me how this is an improvement over understanding the other subpopulations (e.g., parents, smokers, men) which are somewhat more informative subpopulations to understand for public health intervention than a general online audience.

The Conclusions statement seems too strongly worded. The results and the authors in fact do confirm that, within a given country, the images do effect selection since the predicted probability of perceived risk and behaviors were different. Authors should explain why in some of the countries we see image effects. For example:

- Lines 366-369: “in Belgium, the respondents recruited through…image 6…had a lower chance”
- Lines 383-385: “the probability of increased hand hygiene…we found significant differences across images only in Italy and the United Kingdom”
- Lines 401-402: “20 out of our 32 models…found no image effect” suggesting 12 models did find effects.
- Lines 444-446 “in those cases where unexplained image effects on the survey outcomes persisted, the direction of these effects differed depending on the specific outcome and country.”

Even if effects were small, given the large target populations, even small effect sizes can have large public health impacts.

The regression analysis uses relatively few adjustment factors (sex, age, education, month). What other unmeasured factors might be needed? For instance, would race and ethnicity influence results? How about socioeconomic status? These seem to be important variables that were not accounted for. Can authors discuss whether these omitted variables are important?

Have the authors conducted an overall pooled analysis of all countries? If so, what were those results?
