# Supplementary material for: Assessing self-selection biases in Facebook-recruited online surveys: Evidence from the COVID-19 Health Behavior Survey
Source: PLoS One. 2025 Jul 8;20(7):e0326884. doi: 10.1371/journal.pone.0326884 (PMC12237053; doi:10.1371/journal.pone.0326884)
Supplement: Attachment — Submitted filename: Response_to_Reviewers.docx [file pone.0326884.s007.docx]

Dear Editors,

We appreciate the opportunity to revise our manuscript entitled "Assessing self-selection biases in online surveys: Evidence from the COVID-19 Health Behavior Survey " for consideration as a PlosOne research article.

We are thankful to the reviewers for their constructive feedback, which have been instrumental in improving the quality of our work. In response to the reviewers' and editors' comments, we have made substantial revisions to the manuscript. We enclose a detailed point-by-point response to each comment. Here we provide a summary of the major changes that we implemented:

- Results section: We edited this section to present our findings more effectively, addressing the feedback from Reviewer 2. Additionally, we tested the robustness of our findings by including respondents’ employment status in our models, as suggested by Reviewer 1.
- Discussion section: We expanded our interpretation of the potential reasons behind country-specific differences in our results, considering the different COVID-19 health policies in place during our data collection, as suggested by Reviewer 2.
- Conclusion section: We improved this section by elaborating on the applicability of our findings to future research and offering a more nuanced interpretation of our results, as suggested by Reviewer 1.
- Figures and visualizations:
  - We added a visualization of our study design (S1 File, Figure S1), as suggested by Reviewer 2.
  - We included a flowchart (S1 File, Figure S2) detailing the cases excluded at each data preparation step, as per Reviewer 2’s recommendation.
  - We clarified and improved the visual presentation of Figure 2 (now Figure 3 in the revised manuscript), as suggested by Reviewer 2.
- General additions: We revised the manuscript to address instances of unclear or potentially misleading wording.

Additionally, in response to the editors' comment regarding the Role of Funder statement, we have revised it to read as follows: "This study was funded with support from the Max Planck Institute for Demographic Research, part of the Max Planck Society. The funders had no role in study design, data collection and analysis, decision to publish, or preparation of the manuscript.”

We believe that these revisions have significantly improved our manuscript. If further edits or additional changes are recommended, we would be delighted to address them and make any necessary adjustments.

Thank you for your consideration, and we look forward to hearing back from you.

With best regards,

Jessica Donzowa (on behalf of all the authors)

**Journal Requirements:**

1. Please ensure that your manuscript meets PLOS ONE's style requirements, including those for file naming. The PLOS ONE style templates can be found at <https://journals.plos.org/plosone/s/file?id=wjVg/PLOSOne_formatting_sample_main_body.pdf> and <https://journals.plos.org/plosone/s/file?id=ba62/PLOSOne_formatting_sample_title_authors_affiliations.pdf>

Thank you for your reminder regarding PLOS ONE's style requirements. We have carefully reviewed and followed the formal guidelines, including file naming conventions. However, if we have misunderstood any of the requirements, we would be happy to make further adjustments.

2. Thank you for stating the following financial disclosure: "This study was funded with support from the Max Planck Institute for Demographic Research, which is part of the Max Planck Society. " Please state what role the funders took in the study. If the funders had no role, please state: "The funders had no role in study design, data collection and analysis, decision to publish, or preparation of the manuscript." If this statement is not correct you must amend it as needed. Please include this amended Role of Funder statement in your cover letter; we will change the online submission form on your behalf.

We have amended the Role of Funder statement as follows:

“This study was funded with support from the Max Planck Institute for Demographic Research, which is part of the Max Planck Society. The funders had no role in study design, data collection and analysis, decision to publish, or preparation of the manuscript.”

3. In the online submission form, you indicated that "Interested researchers wishing to conduct scientific research can access the survey data upon request and approval. Contact details for accessing the data: Perrotta, Daniela, perrotta@demogr.mpg.de."

All PLOS journals now require all data underlying the findings described in their manuscript to be freely available to other researchers, either 1. In a public repository, 2. Within the manuscript itself, or 3. Uploaded as supplementary information. This policy applies to all data except where public deposition would breach compliance with the protocol approved by your research ethics board. If your data cannot be made publicly available for ethical or legal reasons (e.g., public availability would compromise patient privacy), please explain your reasons on resubmission and your exemption request will be escalated for approval.

Due to privacy regulations and the potential risk of identifying respondents, we cannot share the raw individual survey data used in our analysis. However, we will make the data available upon request to researchers for the purpose of scientific research. Additionally, in the revised version of the manuscript, we have now included the aggregated data underlying the main findings as supplementary material.

4. When completing the data availability statement of the submission form, you indicated that you will make your data available on acceptance. We strongly recommend all authors decide on a data sharing plan before acceptance, as the process can be lengthy and hold up publication timelines. Please note that, though access restrictions are acceptable now, your entire data will need to be made freely accessible if your manuscript is accepted for publication. This policy applies to all data except where public deposition would breach compliance with the protocol approved by your research ethics board. If you are unable to adhere to our open data policy, please kindly revise your statement to explain your reasoning and we will seek the editor's input on an exemption. Please be assured that, once you have provided your new statement, the assessment of your exemption will not hold up the peer review process.

As mentioned in the previous point, we cannot share the individual raw survey data due to data protection regulations. However, in the revised version of the manuscript, we have included the aggregated data underlying the graphs, as well as the code used for the analysis, which allows reproduction of the figures presented in the paper.

5. We note that Figure 1 includes an image of a [patient / participant / in the study]. As per the PLOS ONE policy (http://journals.plos.org/plosone/s/submission-guidelines#loc-human-subjects-research) on papers that include identifying, or potentially identifying, information, the individual(s) or parent(s)/guardian(s) must be informed of the terms of the PLOS open-access (CC-BY) license and provide specific permission for publication of these details under the terms of this license. Please download the Consent Form for Publication in a PLOS Journal (http://journals.plos.org/plosone/s/file?id=8ce6/plos-consent-form-english.pdf). The signed consent form should not be submitted with the manuscript, but should be securely filed in the individual's case notes. Please amend the methods section and ethics statement of the manuscript to explicitly state that the patient/participant has provided consent for publication: “The individual in this manuscript has given written informed consent (as outlined in PLOS consent form) to publish these case details”.

If you are unable to obtain consent from the subject of the photograph, you will need to remove the figure and any other textual identifying information or case descriptions for this individual.

Figure 1 in the manuscript shows the six images used in the advertisements placed on Facebook to recruit survey participants. The images were purchased from two stock image platforms, Adobe Stock and iStockphoto, under licenses permitting republication. The individuals depicted in the stock image were not participants in our study. Consequently, we do not possess or disclose any identifying or potentially identifying information about them.

**Reviewer(s)' Comments to Author:**

We would like to thank the reviewers for their valuable feedback on our manuscript that helped us to substantially strengthen our paper. Below we provide a point-by-point response (highlighted in blue) including a detailed description of all changes in the manuscript.

**Reviewer: 1**

**Comments to the Author**

1. Is the goal of Facebook-based sampling to obtain a representative sample of country’s population or representative sample of Facebook users?

Obtaining a population-representative sample through Facebook-based sampling is challenging, as Facebook users and non-users may differ in unobservable characteristics that cannot be accounted for. In the case of the COVID-19 Health Behavior Survey data collection, the objective was to disseminate the survey as uniformly as possible across key population characteristics, including gender, age, and region of residence. To achieve this, the advertisement campaign was stratified by sex (male and female), age group (18–24, 25–44, 45–64, and 65+ years), and region of residence. While the sample is not fully representative of the entire population, these efforts were designed to ensure a diverse cross-section of respondents alongside those demographic dimensions.

2. Are the images validated measures of salience? While images 1-2 are fairly generic, it is not clear to me that images 3, 4, and 6 are any different in their level or intensity of COVID-19 salience (image 5 would be the most COVID-19 salient).

We thank the reviewer for this comment. Given the unique circumstances of the COVID-19 pandemic and the urgency of launching the survey as quickly as possible, the images used in the recruitment phase were not formally validated. Nevertheless, we believe the images can be grouped into three categories based on the level of reference to the survey topic. Images 1-2 are fairly neutral, while the other images tend to generally refer to respiratory diseases: images 3-4 hint to influenza-like illnesses, and images 5-6 more explicitly to COVID-19 due to the face mask wearing. This is because in the countries of this study, the use of protective face masks was a public health recommendation specifically adopted in response to COVID-19. This measure was still relatively new and contextually specific to COVID-19, particularly in Western countries. We agree with the reviewer that image 5 is the most salient with respect to COVID-19 because it displays an early name of the disease, as we also stated in the paper.

We have clarified the different topic salience between images 3-4 and images 5-6 in the “Materials and Methods” section of the new manuscript at lines 116-129:

“Each advertisement displayed one image selected by Facebook's algorithms from a total of six different advertisement images (Fig 1). Given the unique circumstances of the COVID-19 pandemic and the urgency of launching the survey as quickly as possible, the images used in the survey recruitment phase of our study are not validated measures of salience. However, the images can be grouped into three categories based on how prominently they referred to the survey topic of the COVID-19 pandemic. Images 1 and 2 are fairly neutral, showing individuals exercising outdoors. Images 3 and 4 referred to respiratory illnesses but not to COVID-19 specifically. Lastly, images 5 and 6 explicitly hinted at the survey topic by portraying individuals wearing a protective face mask. This is because in the countries of this study, the use of protective face masks was a public health recommendation specifically in response to COVID-19. This measure was still relatively new and contextually specific to COVID-19, particularly in Western countries. Image 5, in particular, explicitly referred to COVID-19 by displaying an early name of the virus.”

3. Lines 84-89: Are there potential cultural differences in perceptions or interpretations of images?

We appreciate the reviewer raising this important point regarding potential cultural differences in the perception or interpretation of images. We do not have a direct measure of how respondents in the different countries perceived or interpreted the various ad images. However, as the pandemic situation varied between countries during the recruitment period, it is reasonable to assume that images directly related to infectious diseases or the pandemic may have been perceived differently across countries. The different effect of the advertisement images between countries could also reflect differences in cultural perceptions of the advertisement content.

We addressed this aspect in lines 456-463 of the Discussion section in the original manuscript (Lines 454-461 in the revised manuscript), where we discussed variations in public health interventions and the progression of the COVID-19 pandemic across countries:

“Secondly, in those cases where unexplained image effects on the survey outcomes persisted, the direction of these effects differed depending on the specific outcome and country. Contrary to our initial hypothesis, we generally found that higher salience of the survey topic in the advertisement image was associated with lower compliance to wear a face mask. However, this association also displayed variations across countries, for example, the “couple blowing nose” image (image 4) was associated with a higher probability of wearing a face mask in Germany and a lower probability in the United Kingdom.”

Additionally, following comment 11 from reviewer 2, we extended the interpretation of our results in the context of the implemented health policies in the revised manuscript. This expanded discussion can be now found in lines 485 to 500 of the Discussion section, and reads as follows:

“Relating this country classification to our results, we do not observe any systematic pattern linking countries with stronger or more moderate responses to specific advertisement image effects. For instance, while the association between the “woman blowing nose” image (image 3) and higher threat perception to oneself in Italy could suggest increased awareness of symptomatic COVID-19 indicators, this effect is not observed in France or Spain. Similarly, within the group of countries classified as having a moderate response, contrasting image effects emerge. For example, the “couple blowing nose” image (image 4) was associated with higher mask-wearing in Germany but lower in the United Kingdom.

Additionally, we observe similar imagery effects across countries with different COVID-19 policy strategies. For example, the “couple blowing nose” image (image 4) was associated with a lower likelihood of increased handwashing in both Italy and the United Kingdom. In the United States, where COVID-19 quickly became a highly polarizing and political issue [36, 37], this polarization may have amplified self-selection effects, particularly for advertising images explicitly referring to COVID-19 or mask-wearing.”

4. Lines 162-165: what exactly is meant by “interest” in the topic of COVID-19? The term “interest” is too generic. Can authors be more specific here? Do you mean to say concern of infection varied by demographics? Risk of COVID-19 severity varied by demographics?

We thank the reviewer for noticing this. We agree that the word "interest" may be too generic in this context. Here we referred to the perceived concern of a COVID-19 infection, and thus the level of compliance with preventive measures, that may have differed by sex, age, and educational level. To address this, we have replaced the term “interest” with “concern”, which better conveys our intended meaning. The revised sentence in the “Materials and methods” section now reads as follows (lines 167-169):

“Empirical evidence suggests that the level of concern regarding a COVID-19 infection differed across demographic subgroups, with sex, age, and education being among the most important determinants.”

5. Lines 216-219: highly recommend including a flow chart showing n(%) excluded for each reason (similar to a CONSORT or STROBE diagram). Given over half of respondents were dropped from analysis, it is unclear how representative the resulting analytic sample really is.

We thank the reviewer for the opportunity to address this point. We agree that it is important to thoroughly report the number of questionnaires excluded and the reason for their exclusion. For this reason, in the revised version of our manuscript, we have now included a flowchart in S1 File (Fig S2) detailing each of these steps.

Since the ad image is the main predictor in our models, complete data for this variable is essential. Additionally we excluded incomplete and non-substantive responses from the sample, retaining only substantive survey responses. This decision aligns with practices in studies designed to address substantive research questions, where non-substantive responses are typically excluded. Our goal is to ensure that our results are robust and transferable to similar research contexts.

In the revised manuscript, we now refer to this flowchart in line 232-234 where it reads:

“see S2 Fig in S1 File for an overview of the number of questionnaires excluded in each data preparation step”

6. Table S3: Reach and click %s are quite low—6% (1,799,691/30,140,838) of those reached clicked through. Can authors explain whether such low reach and clicks are common? It is difficult to get a sense on how poor or acceptable this level of click through is in terms of recruitment and participation.

We thank the reviewer for highlighting this aspect and for prompting us to provide additional context regarding the click-through rate observed in our study. Across all countries combined, our study reached 30,140,838 and recorded 1,799,691 link clicks, resulting in a click-through rate of 6%. This click-through rate is comparable to, or even higher than, those reported in similar survey studies that used the traffic campaign setting on the Facebook Advertising Platform. For example, Neundorf and Öztürk (2021) conducted Facebook campaigns in the United Kingdom, Turkey, Spain, and the Czech Republic, obtaining click-through rates between 1.5% and 2%.

Reference:

Neundorf, A., & Öztürk, A. (2021). *Recruiting Research Participants through Facebook: Assessing Facebook Advertisement Tools* [Preprint]. Open Science Framework. <https://doi.org/10.31219/osf.io/3g74n>

7. Figure 3: suggests to me that image did in fact result in selection bias since adoption rates did vary by image. On lines 306-309, it says “the level of compliance with hand hygiene recommendations did not differ significantly across images…with the exception of the group of athletes image.” Doesn’t this in fact indicate that there is selection bias? One would have expected there to be NO variation at all.

We thank the reviewer for noticing this oversight. We have revised this sentence accordingly, which now reads as follows in lines 313-315:

“The level of compliance with hand hygiene measures only differs in the Netherlands for the “group of athletes” image (image 2), for which respondents demonstrated a lower likelihood of increasing the frequency of hand washing. ”

8. Figure 4: what is the meaning of the “Low” “Medium” and “High” strata at the top? Please clarify in image.

We apologize that this was not clear. The labels “Low”, “Medium”, and “High” in Figure 4 (now figure 5 in the revised manuscript) refer to the perceived level of threat. Following the reviewer’s comment, we have now clarified this in the figure by updating the labels as "Low perceived threat", "Medium perceived threat", and "High perceived threat", respectively.

9. Month of survey has a profound effect on results; have you tested whether there is interaction between image and month pandemic? It may be that the pandemic month modifies the effect the images have on respondent’s threat perception and behaviors since pandemic month is a proxy for actual risk (i.e., COVID-19 transmission and deaths increased and decreased during the pandemic). The effect of images may differ when actual risk is high (during a peak in cases/deaths) vs when it is low (during a lull in cases/deaths).

We thank the reviewer for this suggestion and agree that exploring the interaction between the month of survey participation and the image effect is relevant to our research question. In response, we have tested whether there is an interaction between the ad image and the month of survey participation by including an interaction term in our models. However, upon inspection, we found that including the interaction terms resulted in inflated standard errors and overly wide confidence intervals. This is due to small case numbers or empty cells in the crosstabulation between our outcome variables (image and month), resulting in sparse data for some combinations of the interaction terms. As a result, our sample size does not allow us to calculate this interaction with adequate precision. Given this limitation, we decided not to include this estimation in the manuscript. In the revised version of the manuscript, we now mention this in lines 405 to 409 of the Results section:

“While exploring the interaction between the month of survey participation and the ad image would be interesting for our research question, small case numbers or empty cells in the crosstabulation between our outcome variables (image and month of survey participation) prevent us from estimating this interaction with sufficient precision.”

10. Unclear how these findings should be applied to future research. Does it mean that investigators can use salient images to maximize recruitment without fear of inducing selection bias?

We appreciate the reviewer's request for clarification on the application of our findings. Our results suggest that the use of salient, thematic images in recruitment materials can be an effective way to maximise reach without systematically introducing selection bias across all outcomes. However, our findings also suggest that image effects may vary by country and specific survey outcome. Thus, while salient images may be useful for broad engagement, we recommend that researchers remain mindful of potential contextual biases and consider pre-testing recruitment materials, where feasible. Furthermore, it is important to note that our results are specific to the unique circumstances of the COVID-19 pandemic. Future studies may benefit from exploring these effects in different contexts, populations, and survey topics to confirm when and where image-driven selection biases may occur. We have revised our Conclusion section to better address these points. In lines 568 to 584 of the revised version of the manuscript it now reads:

“Our findings provide limited support for our initial hypothesis that higher topic salience in the survey recruitment materials would lead to systematically different survey responses. However, within specific countries, we observed that certain recruitment images were associated with different responses regarding perceived COVID-19 risk perception and preventive behaviors. The direction of these effects varied by outcome and country, suggesting that image effects are context-specific and do not uniformly bias responses. It is important to note that our results are specific to the unique circumstances of the COVID-19 pandemic. For future applications of survey recruitment via Facebook advertisements during (health) crises, we conclude that thematic advertisements can effectively reach a broad audience without consistently introducing systematic biases. However, while salient images may be useful for broad engagement, we recommend that researchers remain mindful of potential contextual biases and consider pre-testing recruitment materials, where feasible. Future research should explore whether this effect holds for other social media platforms, non-crisis periods, and different, potentially polarizing survey topics. In conclusion, while topical self-selection had a limited overall impact, context-specific image effects suggest that careful recruitment design remains important in online surveys during health crises.”

11. Lines 519-521: can authors further expand on what they mean by “capturing trends within the general online population”? It is unclear to me how this is an improvement over understanding the other subpopulations (e.g., parents, smokers, men) which are somewhat more informative subpopulations to understand for public health intervention than a general online audience.

We thank the reviewer for this remark. In the Introduction section of the original manuscript (lines 17-31), we presented a few studies that focused on specific subpopulations, such as parents, smokers, or men which offered valuable insights for targeted public health interventions, such as smoking intervention studies. However, in the context of larger public health crises, such as pandemics or natural disasters that may require rapid data collection (for example through social media advertising), it is unclear whether the results from these studies are applicable. For example, two of the cited studies (Stern et al. (2022) and Choi et al. (2017)) focused exclusively on men. In a public health crisis affecting the entire population, these studies do not provide insights on the potential survey response behavior of the general online population. Thus, we believe that our study offers important insights into the response behaviour of the general online population, which cannot be inferred from previous studies.

We have expanded this explanation in the Discussion section of the revised manuscript (lines 562-566), where it now reads:

“Lastly, our emphasis lies in capturing trends within the general online population, which allows for broader applicability in the context of population-wide public health crises, such as pandemics or natural disasters, rather focusing on specific subpopulations — such as parents, smokers, or men — as previous research has done.”

12. The Conclusions statement seems too strongly worded. The results and the authors in fact do confirm that, within a given country, the images do effect selection since the predicted probability of perceived risk and behaviors were different. Authors should explain why in some of the countries we see image effects. For example:

- Lines 366-369: “in Belgium, the respondents recruited through…image 6…had a lower chance”
- Lines 383-385: “the probability of increased hand hygiene…we found significant differences across images only in Italy and the United Kingdom”
- Lines 401-402: “20 out of our 32 models…found no image effect” suggesting 12 models did find effects.
- Lines 444-446 “in those cases where unexplained image effects on the survey outcomes persisted, the direction of these effects differed depending on the specific outcome and country.”

Even if effects were small, given the large target populations, even small effect sizes can have large public health impacts.

We appreciate the reviewer's feedback on our conclusion statement and their suggestion to clarify the observed image effects within certain countries. We agree that these points are important and have addressed them thoroughly in the revised manuscript. Specifically, we have revised our conclusion to provide a more nuanced interpretation of our findings. The revised Conclusion (lines 568 to 584) now reads as follows:

“Our findings provide limited support for our initial hypothesis that higher topic salience in the survey recruitment materials would lead to systematically different survey responses. However, within specific countries, we observed that certain recruitment images were associated with different responses regarding perceived COVID-19 risk perception and preventive behaviors. The direction of these effects varied by outcome and country, suggesting that image effects are context-specific and do not uniformly bias responses. It is important to note that our results are specific to the unique circumstances of the COVID-19 pandemic. For future applications of survey recruitment via Facebook advertisements during (health) crises, we conclude that thematic advertisements can effectively reach a broad audience without consistently introducing systematic biases. However, while salient images may be useful for broad engagement, we recommend that researchers remain mindful of potential contextual biases and consider pre-testing recruitment materials, where feasible. Future research should explore whether this effect holds for other social media platforms, non-crisis periods, and different, potentially polarizing survey topics. In conclusion, while topical self-selection had a limited overall impact, context-specific image effects suggest that careful recruitment design remains important in online surveys during health crises.”

Additionally, to address the reviewer's comment on the variability of image effects across countries, we have expanded the Discussion section to interpret our findings in the context of differing public health interventions and the progression of the COVID-19 pandemic in each country and over time. In summary, our analysis of the advertisement image effects across countries, considering the stringency of their COVID-19 response policies, revealed no clear pattern. Specifically, we did not observe a systematic relationship between the strength of a country’s response to COVID-19 (e.g., strong versus moderate interventions) and the impact of the advertisement images on our outcomes. We acknowledge that these interpretations are based on associations that could not be empirically confirmed within the scope of our analysis. This limitation is now highlighted in the revised Discussion section of the manuscript (lines 466 to 508), which reads as follows:

“To provide some context, we interpret our results in light of the different public health interventions and the progression of the COVID-19 pandemic which varied across the countries and over time [33, 34]. Considering the first phase of the pandemic in spring and summer 2020, the European countries in our analysis can be categorized into countries that had an early and strong response (Italy, France, Spain) and those that had a more moderate response (Germany, Netherlands, Belgium, United Kingdom) [35]. For example, Italy experienced a large COVID-19 outbreak early on in March 2020, prompting early public health interventions in early March. Similar measures were implemented in mid-March in Spain and France [34]. Mandatory masks were introduced relatively early, by mid-March in France and late April in Italy. Some restrictions were relaxed during the summer, depending on the pandemic trends, but then re-imposed when the pandemic worsened in the fall [35]. By contrast, Germany, the Netherlands, Belgium and the United Kingdom had a more moderate response, characterized by a gradual implementation of social distancing and mask-wearing measures [34]. Movement restrictions were implemented by late March, but mask mandates varied. For example, the Netherlands only introduced masks on public transport in June. In general, the implementation of public health interventions also varied by sub-national region (e.g., in Germany movement restrictions were only implemented at regional level, not nationwide) [35].

Relating this country classification to our results, we do not observe any systematic pattern linking countries with stronger or more moderate responses to specific advertisement image effects. For instance, while the association between the “woman blowing nose” image (image 3) and higher threat perception to oneself in Italy could suggest increased awareness of symptomatic COVID-19 indicators, this effect is not observed in France or Spain. Similarly, within the group of countries classified as having a moderate response, contrasting image effects emerge. For example, the “couple blowing nose” image (image 4) was associated with higher mask-wearing in Germany but lower in the United Kingdom.

Additionally, we observe similar imagery effects across countries with different COVID-19 policy strategies. For example, the “couple blowing nose” image (image 4) was associated with a lower likelihood of increased handwashing in both Italy and the United Kingdom. In the United States, where COVID-19 quickly became a highly polarizing and political issue [36, 37], this polarization may have amplified self-selection effects, particularly for advertising images explicitly referring to COVID-19 or mask-wearing.

It is important to note that these interpretations, in relation to country-specific policies, should be approached with caution. While country-specific policies offer additional context for our results, further analysis would be needed to empirically verify potential associations between COVID-19 policy and advertisement design. While this is beyond the focus of this work, it highlights the importance of future survey research using Facebook advertisements during (health) crises to carefully consider the local political environment and public sentiment when designing ads, but also that image effects may be influenced by factors beyond the crisis itself. ”

13. The regression analysis uses relatively few adjustment factors (sex, age, education, month). What other unmeasured factors might be needed? For instance, would race and ethnicity influence results? How about socioeconomic status? These seem to be important variables that were not accounted for. Can authors discuss whether these omitted variables are important?

We thank the reviewer for raising this point. We selected the covariates based on the variables available in the survey. Questions on race or ethnicity were not included in the questionnaire. Similarly, we did not include questions related to income or wealth of respondents. To measure socioeconomic status, we had information on the respondents’ highest level of education and employment status in the past 7 days (e.g., employed, in education, retired). Following the reviewer’s comment, we tested whether including the employment status in our model would affect our results. Employment status was assessed through a list of options, from which respondents could select all that applied to them in the past 7 days: being in paid employment, in education, unemployed and actively looking for a job, unemployed and not actively looking for a job, permanently sick, retired, in military service, or doing housework. We included each response option separately in our model, controlling for each employment status category. We found that our results are robust to the inclusion of the employment status and do not differ from our model without employment status.

In the revised version of the manuscript, we now mention this in line 403 to 405 of the Results section:

“We also tested the inclusion of respondents' employment status over the past seven days in our model and found that our results remain robust, showing no differences compared to the model without employment status.”

14. Have the authors conducted an overall pooled analysis of all countries? If so, what were those results?

We thank the reviewer for this suggestion. In response, we have computed an overall pooled analysis across all countries, controlling for image, gender, age, education, month of survey participation, and country. Both the pooled and country-specific analyses show the same general pattern: the adoption of hand washing was higher than the adoption of wearing a face mask, and the perceived threat to family was higher than the perceived threat to self. However, the pooled analysis overlooks variations in image effects across countries. For example, in the pooled analysis, the perceived threat of COVID-19 to the family was lower for respondents recruited by the "woman wearing mask" image (image 5). Yet, the country-specific analysis reveals that this effect is only present in the United States and not in other countries. Similarly, the "couple blowing nose" image (image 4) was associated with a high likelihood of wearing a face mask in Germany but a lower likelihood in the United Kingdom. Regarding the adoption of handwashing, the pooled analysis shows no significant differences between images, whereas the country-specific analysis reveals image effects for this outcome in Italy and the United Kingdom. Therefore, we believe that the country-specific results are preferable as they highlight the varying effect of the images by country.

**Reviewer: 2**

**Comments to the Author**

Summary

1. Overall, I thought the manuscript focus and hypotheses were quite interesting with regards to leverage-salience theory applications to COVID-19. This topic seems quite relevant, given the need to better understand disease outcomes and population perceptions.

We appreciate the reviewer’s recognition of the relevance of our research topic and theoretical framing.

2. From a constructively critical standpoint, I did find the manuscript results a bit hard to follow. I think the authors could attempt to lay out the results more succinctly. I also think the visualization of the results could be more effectively presented.

We thank the reviewer for this comment. In response, we have revised the Results section of our manuscript to improve readability. Specifically, we ensured that the results for each outcome are consistently presented, referring to either a higher or lower probability where possible. We also streamlined our phrasing to avoid complex or contradictory expressions. As an example, we edited the following sentences:

“In Italy, participants shown the “woman wearing mask” image (image 5) **had a lower chance** (33%, CI: 0.33-0.34) of perceiving COVID-19 **as a high threat** than those shown the “woman blowing nose” image (image 3) (38%, CI: 0.35-0.41). In the United Kingdom, both the “woman blowing nose” image (image 3) (44%, CI: 0.41-0.48) and the “man wearing mask” image (image 6) (43%, CI: 0.41-0.46) had a **higher chance for a higher threat** perception compared to the “couple blowing nose” image (image 4) (34%, CI: 0.28-0.41) and the “woman wearing mask” image (image 5) (39%, CI: 0.38-0.41).”

which now read as follows (Lines 343-350 of the revised manuscript):

“In Italy, participants shown the “woman blowing nose” image (image 3) (38%, CI: 0.35-0.41) were **more likely to perceive a high threat** compared to those recruited through the “woman wearing mask” image (image 5) (33%, CI: 0.33-0.34).

In the United Kingdom, both the “woman blowing nose” image (image 3) (44%, CI: 0.41-0.48) and the “man wearing mask” image (image 6) (43%, CI: 0.41-0.46) were **more likely to perceive a high threat** compared to the “couple blowing nose” image (image 4) (34%, CI: 0.28-0.41) and the “woman wearing mask” image (image 5) (39%, CI: 0.38-0.41).”

Additionally, we have improved the readability of Figure 2 (now Figure 3 in the revised manuscript) by presenting the results as a heat map instead of a stacked bar plot and improved the visualization of significant differences in the model plots (Figure 5 and 6 in the revised manuscript) by adding a vertical line corresponding to the lowest confidence interval (CI). This provides a clearer visual cue to identify overlapping confidence intervals and enhances the reader's ability to assess significant differences between the images.

3. In your appendix 1, your S1 figure (number of participants by ad image and country over time) is a great depiction of the data and might be a nice addition to the actual manuscript (potentially at the beginning of your results?).

We thank the reviewer for their appreciation of our visualisation. In response, we now include it as Figure 2 in the Results section of the revised manuscript, where we describe the number of participants by ad image over time (lines 275-280).

4. I would similarly think about how to better represent model significance. As a reader, I would really like to be able to capture that information visually, or in some way that allows me easily to see how some factors (i.e. month of survey participation) were impactful on model outcomes.

We appreciate the reviewer’s suggestion to visually capture the impact of factors, such as the month of survey participation, on model outcomes. As noted in our detailed response to comments 9 and 10, we carefully considered reporting the stepwise odds ratios or a standardized coefficient diagrams in the main text. However, we concluded that reporting predicted probabilities for the final model in the main text provides a clearer and more direct comparison across categories, aligning better with our study's objectives. We reported the predicted probabilities for each model step in S1 File of the original manuscript, which we also reference in the main text when describing the explanatory effect of the covariates. We considered including these figures in the main text, but we chose to place them in the appendix to avoid overloading the manuscript with additional figures. For a more detailed explanation, please refer to our response to comments 9 and 10.

**Introduction**

5. Well structured. However, near the end of your introduction, you start to discuss the results of your analysis. I might remove that, and instead, formulate some questions that relate to your hypotheses. Do advertising images have a variable effect on survey outcomes? What factors (location, timeframe, population demographics, etc) might influence survey outcomes, given the heterogenous nature of leverage and salience? I might also broaden the hypothesis to say that there are several influential factors which may determine who participates in the survey – which includes time frame of the pandemic – but also includes many other factors, and your intent is to tease out these interactions and effects based on the multi-level structure you have constructed (age/sex/location etc.).

We are pleased to read that the reviewer finds our introduction well structured. Following the reviewer’s suggestion, we have edited the end of the Introduction section (lines 62-71) accordingly. In the revised version of the manuscript it now reads:

“We assume that users who chose to participate in a survey on COVID-19 during the early months of the pandemic were specifically interested in the topic and, therefore, likely differed in their opinions and survey responses regarding the adoption of protective behaviors or threat perception in comparison to users that participated via an image referring to general health behavior. In addition to survey topic interest, several other factors may influence the choice to participate, such as age, gender, education, and the month of survey participation. The focus of this work is to explore how different elements, such as advertising content, participant demographics and timing of participation, shape survey responses related to the adoption of protective behaviors and threat perception.”

**Methods**

6. I can appreciate the detail in the methodology, which is well organized. I may be a little fixated on visualizing outcomes, but you might consider one diagram which depicts the flow of your methodology – even if its in a supplemental appendix. Your approach focuses on survey data from this 2020 time frame, based on 6 differing ads which have some form of increasing COVID-19 prominence. Then you analyze based on several predictor factors. That could be presented in one diagram – so the reader would immediately be able to grasp the approach and how the models are applied.

We thank the reviewer for their appreciation of the detail and structure of our methodology and the suggestion to include a visualization of our study design. In response, we have created a figure that visualizes our study design and included it in the supplementary material (S1 Fig). We reference this figure at Line 218 of the revised version of the manuscript, and we report it here below for the reviewers’ convenience.

7. Also – you mention a total of 32 models, I’m assuming based on the changing factor levels. If that is clearly defined, maybe I just missed it – but if not, id include that. Potentially in the diagram that I’m suggesting above.

We apologize for any confusion regarding this part. The total of 32 models results from calculating models for four outcomes across eight countries, representing the number of final models that include all covariates. In the revised version of the manuscript, we have clarified this point in the Materials and methods section by adding the following sentence at line 220:

“Considering the four outcomes across eight countries, this results in 32 final models, each including all covariates.”

**Results**

8. Page 9: “Fig 2 shows the variation in the levels of perceived threat towards COVID-19 that respondents reported for themselves and for their family, across the various countries and advertisement images. Respondents generally perceived COVID-19 as a higher threat to their family than to themselves.” I couldn’t tell from Figure 2 that respondents perceived COVID-19 as a higher threat to their family than themselves? Am I missing something?

Following the reviewer’s comment, we have improved Figure 2 (now Figure 3 in revised manuscript) for better readability and now report the level of threat perception by image in the form of a heat map instead of a stacked bar plot. We report it here below for the reviewers’ convenience.

9. Also – if your models are binomial and multinomial logistic regressions, then a prominent summary statistic would be the odds ratios. I see you are reporting these in your S2 appendix. Maybe summarizing the odds ratios for the most influential models might be interesting to report in the manuscript? Again, if I missed that in the manuscript, my apologies.

We thank the reviewer for this thoughtful suggestion and for their valuable insight regarding the use of odds ratios. We carefully considered this during our analysis. After further reflection, we believe that reporting the predicted probabilities in the manuscript better addresses our research question. This is because predicted probabilities allow for a more direct comparison of all images with one another, whereas odds ratios are inherently tied to a reference category, which may limit the interpretability in this context. We believe that this approach is more aligned with the objectives of our study.

10. You could also present a standardized coefficient diagram that indicates those factors which are significant, with their odds ratios/Cis. Maybe that’s already in the supplementals. If so – one of those diagrams in the results would seem to be very useful.

We thank the reviewer for this thoughtful suggestion. We understand a standardized coefficient plot to graphically represent the relative importance of predictors using standardized coefficients. In the context of linear regression, standardized coefficients allow for a unitless interpretation, indicating how many standard deviations the dependent variable changes per standard deviation change in the predictor variable. However, in logistic regression, the predicted outcome is not the dependent variable itself but the probability of the outcome occurring. This distinction complicates the calculation and interpretation of standardized coefficients, as the dependent variable is transformed into a logit scale that lacks a straightforward mean or standard deviation. In addition, our model uses only categorical predictors, where regression coefficients represent changes in the log odds of the outcome relative to a reference category. Standardizing these coefficients is less meaningful because the concept of a "standard deviation" increase is not directly applicable to categorical variables. For these reasons, we chose not to include standardized coefficients in the manuscript.

If the reviewer’s comment instead refers to showing the predicted probabilities for different model steps, we note that these are reported in S1 File of the original manuscript and we referenced them in the main text when describing the explanatory effect of the covariates. While we considered including these figures in the main text, we decided to retain them in the appendix to avoid overloading the main text with additional figures. If we have misunderstood the comment, we would be happy to clarify it further.

**Discussion**

11. I think your discussion would benefit by giving some thought as to WHY you think some of this variability exists – either across location or the differing interactions of influential factors on each of the images. My sense is – some of this language is more appropriate for the results section. For example: you report some of the locational differences (based on country). Were their known policy or covid-19 spread variations based on county – that might be associated with what your results are indicating? I realize that’s not the focus of this work – but I’m interested in why we are seeing these results, and what implications we might be infer?

We thank the reviewer for this thoughtful suggestion. We agree that further consideration of country-specific factors could provide valuable context for the observed variability in threat perception and protective behaviors we observed in relation to the advertisement designs. In response, we have expanded the description of the COVID-19 policies in place in the countries included in our analysis and have moved this part from the Limitations section to the Discussion section in order to better contextualize these factors alongside our results. Additionally, we have expanded the Discussion section to interpret our findings in the context of differing public health interventions and the progression of the COVID-19 pandemic in each country and over time. Specifically, when examining the advertisement image effects across countries and considering the stringency of their COVID-19 response policies, we found no clear pattern. In other words, we did not observe a systematic relationship between the type of response to COVID-19 in each country (e.g., strong versus moderate interventions) and the impact of the advertisement images on our outcomes. We recognize that these interpretations are based on associations that could not be empirically confirmed within the scope of our analysis. In the revised manuscript, we highlight this aspect in the Discussion section (lines 466-508) which we report here below for the reviewers’ convenience.

“To provide some context, we interpret our results in light of the different public health interventions and the progression of the COVID-19 pandemic which varied across the countries and over time [33, 34]. Considering the first phase of the pandemic in spring and summer 2020, the European countries in our analysis can be categorized into countries that had an early and strong response (Italy, France, Spain) and those that had a more moderate response (Germany, Netherlands, Belgium, United Kingdom) [35]. For example, Italy experienced a large COVID-19 outbreak early on in March 2020, prompting early public health interventions in early March. Similar measures were implemented in mid-March in Spain and France [34]. Mandatory masks were introduced relatively early, by mid-March in France and late April in Italy. Some restrictions were relaxed during the summer, depending on the pandemic trends, but then re-imposed when the pandemic worsened in the fall [35]. By contrast, Germany, the Netherlands, Belgium and the United Kingdom had a more moderate response, characterized by a gradual implementation of social distancing and mask-wearing measures [34]. Movement restrictions were implemented by late March, but mask mandates varied. For example, the Netherlands only introduced masks on public transport in June. In general, the implementation of public health interventions also varied by sub-national region (e.g., in Germany movement restrictions were only implemented at regional level, not nationwide) [35].

Relating this country classification to our results, we do not observe any systematic pattern linking countries with stronger or more moderate responses to specific advertisement image effects. For instance, while the association between the “woman blowing nose” image (image 3) and higher threat perception to oneself in Italy could suggest increased awareness of symptomatic COVID-19 indicators, this effect is not observed in France or Spain. Similarly, within the group of countries classified as having a moderate response, contrasting image effects emerge. For example, the “couple blowing nose” image (image 4) was associated with higher mask-wearing in Germany but lower in the United Kingdom.

Additionally, we observe similar imagery effects across countries with different COVID-19 policy strategies. For example, the “couple blowing nose” image (image 4) was associated with a lower likelihood of increased handwashing in both Italy and the United Kingdom. In the United States, where COVID-19 quickly became a highly polarizing and political issue [36, 37], this polarization may have amplified self-selection effects, particularly for advertising images explicitly referring to COVID-19 or mask-wearing.

It is important to note that these interpretations, in relation to country-specific policies, should be approached with caution. While country-specific policies offer additional context for our results, further analysis would be needed to empirically verify potential associations between COVID-19 policy and advertisement design. While this is beyond the focus of this work, it highlights the importance of future survey research using Facebook advertisements during (health) crises to carefully consider the local political environment and public sentiment when designing ads, but also that image effects may be influenced by factors beyond the crisis itself. ”
