# Supplementary material for: Assessing self-selection biases in Facebook-recruited online surveys: Evidence from the COVID-19 Health Behavior Survey
Source: PLoS One. 2025 Jul 8;20(7):e0326884. doi: 10.1371/journal.pone.0326884 (PMC12237053; doi:10.1371/journal.pone.0326884)
Supplement: Attachment — Submitted filename: Response_to_Reviewers_auresp_2.docx [file pone.0326884.s008.docx]

Dear Editors,

We appreciate the opportunity to revise our manuscript, originally entitled "Assessing self-selection biases in online surveys: Evidence from the COVID-19 Health Behavior Survey" for consideration as a PlosOne research article.

We are thankful to the reviewers for their constructive feedback throughout the first and second rounds of revisions, which have been instrumental in improving the quality of our work. We enclose a detailed point-by-point response to each comment. Here we provide a summary of the major changes that we implemented:

- We revised the title of the manuscript to explicitly specify Facebook recruitment. The revised title is “Assessing self-selection biases in Facebook-recruited online surveys: Evidence from the COVID-19 Health Behavior Survey”
- We addressed the potential algorithmic bias by analyzing its potential impact on our sample composition and adding tables in the S1 File showing the number of impressions by ad image, gender, and age for each country.
- We expanded the discussion on the lack of a survey interest measure in order to explore its potential implications for the interpretation of our results and suggest ways future studies might address this issue.

We believe that these revisions have significantly strengthened our manuscript. If further edits or additional changes are recommended, we would be delighted to address them and make the necessary adjustments.

Thank you for your time and consideration, and we look forward to hearing back from you.

With best regards,

Jessica Donzowa (on behalf of all the authors)

**Journal Requirements:**

1. Please review your reference list to ensure that it is complete and correct. If you have cited papers that have been retracted, please include the rationale for doing so in the manuscript text, or remove these references and replace them with relevant current references. Any changes to the reference list should be mentioned in the rebuttal letter that accompanies your revised manuscript. If you need to cite a retracted article, indicate the article’s retracted status in the References list and also include a citation and full reference for the retraction notice.

We thank the editor for the reminder. We have checked our reference list and did not find any retracted papers. However, we noticed that some references were missing the DOI or a stable link, which we have now added in the revised version of the manuscript. The updated references are the following, listed in the order they appear in the manuscript:

- Wenz A. Do Distractions During Web Survey Completion Affect Data Quality? Findings From a Laboratory Experiment. Social Science Computer Review. 2019; p. 14. doi:10.1177/0894439319851503.
- Stern MJ, Fordyce E, Carpenter R, Viox MH, Michaels S, Harper C, et al. Evaluating the Data Quality of a National Sample of Young Sexual and Gender Minorities Recruited Using Social Media: The Influence of Different Design Formats. Social Science Computer Review. 2022;40(3):663–677. doi: 10.1177/0894439320928240.
- Groves RM. Understanding The Decision to Participate in a Survey on JSTOR. 1992; Available from: https://www.jstor.org/stable/2749203.
- Groves RM, Singer E, Corning A. Leverage-Saliency Theory of Survey Participation. Public Opinion Quarterly. 2000;64(3):299–308. doi: 10.1086/317990.
- Lehdonvirta V, Oksanen A, Räsänen P, Blank G. Social Media, Web, and Panel Surveys: Using Non-Probability Samples in Social and Policy Research. Policy & Internet. 2021;13(1):134–155. doi: 10.1002/poi3.238.

**Reviewer(s)' Comments to Author:**

**Reviewer 2:**

The authors have complied with all the requested revisions and feedback. Recommend acceptance of the manuscript.

We thank the reviewer for the positive feedback and recommendation to accept our manuscript. We greatly appreciate their support throughout the review process.

**Reviewer 3:**

This study investigates whether self-selection bias affects responses in online surveys when participants are recruited through Facebook advertisements with varying degrees of topic salience. Using a large cross-national dataset (N=120,184) from eight countries, the authors find minimal self-selection bias, suggesting that concerns over bias in social media-recruited surveys may be overstated. The study is methodologically rigorous, well-written, and highly relevant to discussions on the validity of online survey data. However, while the paper makes a strong contribution, there are some limitations and areas for improvement that should be addressed before publication. Below, I provide specific comments on the title, strengths, and areas for revision.

We thank the reviewer for their thoughtful and encouraging feedback. We appreciate their positive assessment of our study’s methodological rigor, writing quality, and contribution to discussions on online survey quality. The additional points raised by the reviewer provided valuable insights that helped us further refine and improve our manuscript. We addressed the specific points in the responses below and incorporated the suggestions into the revised version of the manuscript.

**Major Comment: Title Revision Needed**

**Clarify That Findings Are Facebook-Specific**

- The current title, Assessing Self-Selection Biases in Online Surveys, is too broad given that all recruitment was conducted via Facebook ads.
- Since self-selection bias may differ across platforms (e.g., LinkedIn, Twitter, Instagram), the title should explicitly mention Facebook to avoid overgeneralization.
- Suggested revision: Assessing Self-Selection Biases in Facebook-Recruited Online Surveys: Evidence from the COVID-19 Health Behavior Survey
- Alternatively, the authors could add a phrase in the abstract emphasizing that findings may not generalize to other platforms.

We thank the reviewer for raising this point. We agree that it is important to address the platform-specific nature of our findings from social media-recruited surveys in general, and to make this explicit in the title. We agree with the title suggested by the reviewer, “Assessing self-selection biases in Facebook-recruited online surveys: Evidence from the COVID-19 Health Behavior Survey”, and incorporated this change in the revised version of our manuscript.

**Strengths:**

1. **Timely and Important Topic**

- The study addresses a key methodological concern in online survey research—whether recruitment via social media advertisements introduces selection bias.
- Given the widespread use of online surveys, particularly during and after the COVID-19 pandemic, this research is highly relevant.

2. **Large and Cross-National Dataset**

- The inclusion of 120,184 respondents from eight countries enhances the generalizability of the findings.
- Few studies analyze cross-national differences in self-selection bias, making this study a valuable contribution.

3. **Innovative Use of Ad Image Variations**

- The quasi-experimental design, which compares survey responses from participants recruited through different advertisement images, is an innovative approach to testing self-selection bias.
- This provides empirical evidence on how survey topic salience in recruitment ads affects participation and response patterns.

4. **Strong Statistical Methods**

- The study uses multinomial and binomial logistic regression models, effectively controlling for demographics (age, gender, education), country context, and timing of survey participation.
- The use of stepwise regression models to test for the persistence of self-selection effects adds credibility to the conclusions.

5. **Important Practical Implications**

- The study challenges the common assumption that social media-based surveys suffer from strong self-selection bias.
- This finding is particularly useful for researchers designing future online surveys and suggests that Facebook-based survey recruitment remains a viable method.

We thank the reviewer for their thoughtful feedback on our manuscript and for pointing out the strengths of our study, including its relevance and timeliness, the use of a large cross-national dataset, the innovative use of ad image variations and appropriate statistical methods, and its important practical implications for survey research.

**Areas for Improvement & Revisions:**

1. **Limited Generalizability Beyond Facebook**

- The authors should explicitly clarify that findings may not generalize to other social media platforms with different ad targeting mechanisms.
- Recommendation: Add a paragraph in the discussion section acknowledging that platform algorithms may vary, affecting how self-selection bias manifests elsewhere.

We thank the reviewer for pointing out the important influence of platform-specific algorithms on social media-based research. We agree that it is important to acknowledge the challenges and limitations of generalizing findings beyond a specific social media platform due to the unknown mechanisms in the ads distribution.

In the original version of this manuscript, we mentioned this aspect in the Limitations section (lines 568 - 572):

“Further, we need to acknowledge that the results between social media platforms cannot be directly compared as the advertisement structure and platform usage pattern may differ between them. However, as the majority of studies that use social media as a recruitment tool rely on Facebook, the results presented here still offer valuable insights for those studies using Facebook early during the pandemic.”

To address this aspect more explicitly, we have also added a sentence in the Discussion section highlighting that the results may not be generalizable beyond the Facebook platform. Lines 510 through 514 of the revised manuscript now read:

“An important consideration is that our findings may not generalize beyond Facebook, as platform-specific algorithms with targeting mechanisms unknown to researchers may influence self-selection bias differently across social media platforms. We discuss additional limitations of our study in the next section.”

**2. Lack of Pre-Survey Interest Measurement**

- The authors assume that ad images influenced self-selection bias, but they do not measure participants’ pre-existing interest in COVID-19 topics.
- Would it be possible to use post-hoc measures (e.g., interest in public health topics) to strengthen the interpretation of self-selection effects?
- At minimum, the authors should discuss this limitation and suggest how future studies might address it.

We appreciate the reviewer's point about the limitation of not measuring participants' pre-existing interest in the COVID-19 topics. In the Limitations section of the original manuscript (lines 535-538), we acknowledged the lack of an independent measure of respondents' initial interest in the survey topic. In response to the reviewer's comment, we have expanded this section to discuss the potential implications for interpreting our results and suggest ways future studies might address this issue.

The revised version of the manuscript now reads (lines 538-547):

"Since respondents were recruited during the early months of the pandemic, it is reasonable to assume a heightened general interest in COVID-19. This may have led to an underestimation of the role of the advertisement image in self-selection bias, as there was no direct measure of respondents' interest in the survey topic. Future studies could address this issue by including a question about respondents' interest in the survey topic. When using ad images with different topics, researchers could ask about respondents' interest in all topics mentioned in the ads—since they only know which ad image respondents entered after data collection—or design separate surveys with unique links for each ad image, explicitly measuring interest in the displayed topic."

**3. National Contexts & Policy Differences Not Fully Accounted For**

- COVID-19 policies and public health messaging varied significantly across the eight countries in the study.
- Did differences in government mandates, media coverage, or pandemic severity influence how participants perceived survey topics?
- The authors should expand their discussion on how these external factors might have affected responses.

We appreciate the reviewer’s comments regarding the potential influence of national contexts, such as government mandates, media coverage, and pandemic severity, on participants' perceptions of the survey topic. However, as noted in our response to comment two, we cannot make definitive claims about how these external factors shaped participants' perceptions, as we did not directly measure them.

In the original manuscript (lines 469-510), we had already provided an overview of the public health policies implemented across the eight countries in our study and analyzed their relationship to our findings. We observed no consistent pattern between image effects and countries, even when categorized by the stringency of their COVID-19 response measures during the initial months of the pandemic. One possible explanation is that individual-level factors—such as personal interest in COVID-19 or prior exposure to related information—may have played a more significant role than country-level policies in shaping participants’ engagement with the survey.

**4. Potential Algorithm Bias in Ad Distribution**

- The paper notes that Facebook’s algorithm optimized for engagement, meaning some ad images were shown more frequently than others.
- However, it is unclear how much this influenced sample composition.
- Would it be possible to report how often each ad image was shown across demographic groups?
- If this data is unavailable, the authors should discuss the potential impact of algorithmic bias in the limitations section.

We thank the reviewer for raising this point and suggesting an analysis of these metrics by demographic groups. In response, we added three additional tables to the S1 file (Table S5, S6, and S7) that show the number of impressions by gender, age, and advertisement design for the eight countries in our study. This additional information by age and gender improves the understanding of how the ads were displayed and provides insights into the age and gender composition of our sample by advertisement image. In the revised version of the manuscript, we now refer to these tables in the Results section (lines 290 to 295), where it reads:

“A more detailed description of the covariates by ad image, as well as impressions by age, gender and ad image, can be found in the supporting information S1 File (see S4 to S7 Tables in S1 File). In general, the age and gender composition by ad image is consistent with the number of impressions (i.e., the number of times the ad was displayed) recorded for the ad sets targeted by gender and age.”

We also added the main finding from these tables into the “Covariates by advertisement image” section of the S1 file, where we describe the sample composition by age, gender, and advertisement design. On page eight of the revised S1 file, it now reads:

“In general, the age and gender composition by ad image in each country aligns with the number of impressions (i.e., the number of times the ad was displayed) recorded for the ad sets targeting by gender and age (Tables S5 to S7). Taking the United States as an example, only 15% of the respondents recruited through the “group of athletes” image (image 2) reported being female in the survey, and the median age for this image is higher than the overall median age for the country in the survey–63 compared to 58. Looking at the number of impressions, we see that the “group of athletes” image (image 2) received a significantly higher share of impressions in the two older age groups (i.e., 45-65 and 65+) with about 54% compared to just 6% for the ad sets targeted to women in those same age groups.”

**5. Unclear Impact on Long-Term Online Survey Practices**

- While the findings suggest that self-selection bias is minimal in social media-recruited surveys, does this hold outside of a crisis situation like COVID-19?
- The authors should consider whether the unique context of the pandemic (increased time online, heightened public health awareness) might have reduced typical self-selection effects.
- Future research directions should explore non-crisis topics and different recruitment methods.

We thank the reviewer for raising the importance of the specific COVID-19 crisis context in which our survey was conducted. We agree that it is important to acknowledge that our results are specific to this health crisis and may not generalize to other potentially polarizing topics. We already emphasized this aspect in the original manuscript, for instance in the Conclusion section (line 590) where we limit the applicability of our results to “survey recruitment via Facebook advertisements during (health) crises”. In addition, we state the following in the Conclusion section of the original manuscript (lines 595-597):

“Future research should explore whether this effect holds for other social media platforms, non-crisis periods, and different, potentially polarizing survey topics.”

**Final Recommendation:**

This study makes a strong and novel contribution to the field of online survey methodology. I recommend acceptance with minor revisions to strengthen the title, discussion on generalizability, and analysis of potential biases. With these refinements, the paper will be even more impactful for researchers designing future online surveys.

Would the authors be open to modifying the title to specify Facebook recruitment? Additionally, addressing algorithmic bias and survey interest measurement would make the findings more robust.

I appreciate the opportunity to review this manuscript and look forward to seeing the final version.

We thank the reviewer for their thoughtful and supportive feedback. We appreciate the recognition of our study’s novel contribution to online survey methodology and the valuable suggestions for further strengthening the manuscript.

In response to the recommendations, we have revised the title to explicitly specify Facebook recruitment. Additionally, we have discussed the algorithmic bias and the measurement of survey interest more explicitly to further enhance the interpretation of our findings.
