# Supplementary material for: Assessing self-selection biases in Facebook-recruited online surveys: Evidence from the COVID-19 Health Behavior Survey
Source: PLoS One. 2025 Jul 8;20(7):e0326884. doi: 10.1371/journal.pone.0326884 (PMC12237053; doi:10.1371/journal.pone.0326884)
Supplement: Attachment — Submitted filename: Response_to_Reviewers_auresp_3.docx [file pone.0326884.s009.docx]

Dear Editors,

We appreciate the opportunity to revise our manuscript, entitled "Assessing self-selection biases in Facebook-recruited online surveys: Evidence from the COVID-19 Health Behavior Survey" for consideration as a PLOS One research article.

We are thankful to the reviewers for their constructive feedback throughout the three rounds of revisions, which has been instrumental in improving the quality and clarity of our work. We enclose a detailed point-by-point response to each comment. Here we provide a summary of the major changes that we implemented:

- We adjusted the wording in the abstract and discussion to more clearly reflect the platform-specific nature of our findings.
- We expanded our suggestions for how future research could measure respondents’ reactions to specific ad images.
- We emphasized more explicitly the opaque nature of Facebook’s ad algorithm and the limited generalizability of our findings to other public health contexts in the limitations section.

We believe that these revisions have significantly strengthened our manuscript. If further edits or additional changes are recommended, we would be delighted to address them and make the necessary adjustments.

Thank you for your time and consideration, and we look forward to hearing back from you.

With best regards,

Jessica Donzowa (on behalf of all the authors)

**Journal Requirements:**

1. Please review your reference list to ensure that it is complete and correct. If you have cited papers that have been retracted, please include the rationale for doing so in the manuscript text, or remove these references and replace them with relevant current references. Any changes to the reference list should be mentioned in the rebuttal letter that accompanies your revised manuscript. If you need to cite a retracted article, indicate the article’s retracted status in the References list and also include a citation and full reference for the retraction notice.

We thank the editor for the reminder regarding the reference list. We have carefully checked all references cited in the manuscript. We have not identified any retracted articles among the cited works. We have revised the references to ensure that they follow the Vancouver citation style. This includes standardizing capitalization from titles, abbreviating journal names, adding volume, issue, and page numbers where needed, and correcting the citation of preprints. The updated references are listed below in the order in which they appear in the manuscript:

Wenz A. Do distractions during web survey completion affect data quality? Findings from a laboratory experiment. Soc Sci Comput Rev. 2021;39(1):148-161. doi:10.1177/0894439319851503

Thornton L, Batterham PJ, Fassnacht DB, Kay-Lambkin F, Calear AL, Hunt S. Recruiting for health, medical or psychosocial research using Facebook: systematic review. Internet Interv. 2016;4:72–81. doi:10.1016/j.invent.2016.02.001

Grow A, Perrotta D, Del Fava E, Cimentada J, Rampazzo F, Gil-Clavel S, et al. Is Facebook’s advertising data accurate enough for use in social science research? Insights from a cross-national online survey. J R Stat Soc Ser A Stat Soc. 2022;185(Supplement 2):343–S363. doi:10.1111/rssa.12948

Pötzschke S, Braun M. Migrant sampling using Facebook advertisements: a case study of Polish migrants in four European countries. Soc Sci Comput Rev. 2017;35(5):633–653. doi:10.1177/0894439316666262

Nelson LM, Simard JF, Oluyomi A, Nava V, Rosas LG, Bondy M, et al. US public concerns about the COVID-19 pandemic from results of a survey given via social media. JAMA Intern Med. 2020;180(7):1020–1022. doi:10.1001/jamainternmed.2020.1369

Wang PW, Lu WH, Ko NY, Chen YL, Li DJ, Chang YP, et al. COVID-19-related information sources and the relationship with confidence in people coping with COVID-19: Facebook survey study in Taiwan. J Med Internet Res. 2020;22(6):e20021. doi:10.2196/20021

Qiu J, Shen B, Zhao M, Wang Z, Xie B, Xu Y. A nationwide survey of psychological distress among Chinese people in the COVID-19 epidemic: implications and policy recommendations. Gen Psychiatr. 2020;33(2):e100213. doi:10.1136/gpsych-2020-100213

Gaetano AD, Bajardi P, Gozzi N, Perra N, Perrotta D, Paolotti D. Behavioral changes associated with COVID-19 vaccination: cross-national online survey. J Med Internet Res. 2023;25(1):e47563. doi:10.2196/47563

De Man J, Campbell L, Tabana H, Wouters E. The pandemic of online research in times of COVID-19. BMJ Open. 2021;11(2):e043866. doi:10.1136/bmjopen-2020-043866

Batterham PJ. Recruitment of mental health survey participants using Internet advertising: content, characteristics and cost effectiveness: Recruitment using Internet advertising. Int J Methods Psychiatr Res. 2014;23(2):184–191. doi:10.1002/mpr.1421

Bethlehem J. Selection bias in web surveys. Int Stat Rev. 2010;78(2):161-188. doi:10.1111/j.1751-5823.2010.00112.x

Kühne S, Zindel Z. Using Facebook and Instagram to recruit web survey participants: a step-by-step guide and application. Surv Methods Insights Field. 2020;doi:10.13094/SMIF-2020-00017

Ramo DE, Rodriguez TMS, Chavez K, Sommer MJ, Prochaska JJ. Facebook recruitment of young adult smokers for a cessation trial: Methods, metrics, and lessons learned. Internet Interv. 2014;1(2):58-64. doi:10.1016/j.invent.2014.05.001

Bennetts SK, Hokke S, Crawford S, Hackworth NJ, Leach LS, Nguyen C, et al. Using paid and free Facebook methods to recruit Australian parents to an online survey: an evaluation. J Med Internet Res. 2019;21(3):e11206. doi:10.2196/11206

Machado NM, Gomide HP, Bernardino HS, Ronzani TM. Facebook recruitment of smokers: comparing gain- and loss-framed ads for the purposes of an internet-based smoking cessation intervention. Cad Saude Publica. 2019;35 (10):e00151318. doi:10.1590/0102-311X00151318

August T, Oliveira N, Tan C, Smith N, Reinecke K. Framing effects: choice of slogans used to advertise online experiments can boost recruitment and lead to sample biases. Proc ACM Hum Comput Interact. 2018;2(CSCW):Article 22. doi:10.1145/3274291

Choi I, Milne DN, Glozier N, Peters D, Harvey SB, Calvo RA. Using different Facebook advertisements to recruit men for an online mental health study: engagement and selection bias. Internet Interv. 2017;8:27-34. doi:10.1016/j.invent.2017.02.002

Stern MJ, Fordyce E, Carpenter R, Viox MH, Michaels S, Harper C, et al. Evaluating the data quality of a national sample of young sexual and gender minorities recruited using social media: the influence of different design formats. Soc Sci Comput Rev. 2022;40(3):663–677. doi: 10.1177/0894439320928240

Neundorf A, Öztürk A. Advertising online surveys on social media: how your advertisements affect your samples [preprint]. OSF. 2022. Available from: https://osf.io/84h3t/

Perrotta D, Grow A, Rampazzo F, Cimentada J, Del Fava E, Gil-Clavel S, et al. Behaviours and attitudes in response to the COVID-19 pandemic: EPJ Data Sci. 2021;10(1):17. doi:10.1140/epjds/s13688-021-00270-1

Groves RM, Cialdini RB, Couper MP. Understanding the decision to participate in a survey. Public Opin Q. 1992;56(4):475-495. Available from: https://www.jstor.org/stable/2749203

Groves RM, Singer E, Corning A. Leverage-saliency theory of survey participation. Public Opin Q. 2000;64(3):299–308. doi:10.1086/317990

Lehdonvirta V, Oksanen A, Räsänen P, Blank G. Social media, web, and panel surveys: Using non-probability samples in social and policy research. Policy Internet. 2021;13(1):134-155. doi:10.1002/poi3.238

Grow A, Perrotta D, Del Fava E, Cimentada J, Rampazzo F, Gil-Clavel S, et al. Addressing public health emergencies via Facebook surveys: Advantages, Challenges, and Practical Considerations. J Med Internet Res. 2020;22(12):e20653. doi:10.2196/20653

Neundorf A, Öztürk A. How to improve representativeness and cost-effectiveness in samples recruited through meta: a comparison of advertisement tools. PLoS ONE. 2023;18(2):e0281243. doi:10.1371/journal.pone.0281243

Pötzschke S, Weiß B, Daikeler J, Silber H, Beuthner C. A guideline on how to recruit respondents for online surveys using Facebook and Instagram: using hard-to-reach health workers as an example [Preprint]. Survey Guidelines. 2023. Available from: https://doi.org/10.15465/GESIS-SG_EN_045

Galasso V, Pons V, Profeta P, Becher M, Brouard S, Foucault M. Gender differences in COVID-19 attitudes and behavior: panel evidence from eight countries. Proc Natl Acad Sci U S A. 2020;117(44):27285–27291. doi:10.1073/pnas.2012520117

Oreffice S, Quintana-Domeque C. Gender inequality in COVID-19 times: evidence from UK prolific participants. J Demogr Econ. 2021;87(2):261–287. doi:10.1017/dem.2021.2

Maxfield M, Pituch KA. COVID-19 worry, mental health indicators, and preparedness for future care needs across the adult lifespan. Aging Ment Health. 2021;25(7):1273–1280. doi:10.1080/13607863.2020.1828272

Lüdecke D, von dem Knesebeck O. Protective behavior in course of the COVID-19 outbreak—survey results from Germany. Front Public Health. 2020;8:572561. doi:10.3389/fpubh.2020.572561

Perrotta D, Fava ED, Zagheni E. The role of vulnerability in mediating the relationship between threat perception and the use of face masks in the context of COVID-19. Proceedings of the International AAAI Conference on Web and Social Media. 2021. Available from: https://workshop-proceedings.icwsm.org/pdf/2021_43.pdf

Yuri Bruinen de Bruin, Anne-Sophie Lequarre, Josephine McCourt, Peter Clevestig, Filippo Pigazzani, Maryam Zare Jeddi, et al. Initial impacts of global risk mitigation measures taken during the combatting of the COVID-19 pandemic. Saf Sci. 2020;128:104773. doi:10.1016/j.ssci.2020.104773

Woskie LR, Hennessy J, Espinosa V, Tsai TC, Vispute S, Jacobson BH, et al. Early social distancing policies in Europe, changes in mobility & COVID-19 case trajectories: insights from spring 2020. PLoS ONE. 2021;16(6):e0253071. doi:10.1371/journal.pone.0253071

de Saint-Phalle E. Masks and social distancing during COVID-19. In: Covid-19 containment policies in Europe. International Series on Public Policy. Chem: Springer Nature; 2024. Available from: https://link.springer.com/book/10.1007/978-3-031-52096-9

Hart PS, Chinn S, Soroka S. Politicization and polarization in COVID-19 news coverage. Sci Commun. 2020;42(5):679-697.doi:10.1177/1075547020950735

Hubner A. How did we get here? A framing and source analysis of early COVID-19 media coverage. Commun Res Rep. 2021;38(2):112-120. doi:10.1080/08824096.2021.1894112

Zindel Z, Kühne S, Zagheni E, Perrotta D. Polarizing ads to recruit survey respondents: A comparative study of Facebook ads and their impact on sample composition. Presented at: European Survey Research Association Conference; 2023. Available from: https://www.europeansurveyresearch.org/conf2023/prog.php?sess=52#229

**Reviewer(s)' Comments to Author:**

**Reviewer 3:**

I appreciate the authors’ thoughtful and thorough revisions to the manuscript. The updated version demonstrates meaningful improvements in clarity, methodological transparency, and contextualization of findings. The manuscript now offers a more precise contribution to the literature on online survey recruitment and selection bias, particularly as it pertains to Facebook.

We thank the reviewer for the positive feedback and recognition of our efforts in revising the manuscript. We appreciate that the revisions were seen as meaningful improvements in clarity, methodological transparency, and contextualization of our findings. We appreciate the reviewers' time and careful review throughout the revision process.

**Strengths of the Revision:**

The updated title and discussion now appropriately reflect that findings are specific to Facebook-recruited samples. The authors have added valuable discussion of the limitations introduced by Facebook’s ad delivery system, including a new analysis of ad impressions by demographic group. The paper now clearly acknowledges the absence of a direct measure of pre-survey interest and offers reasonable suggestions for future studies to address this gap. The presentation of the modeling strategy, outcomes, and supplementary tables has been improved and is easier to follow. The authors have responded well to concerns about the generalizability of findings across countries and health contexts, noting the potential influence of media coverage and national policies.

We thank the reviewer for their encouraging comments. We appreciate the recognition of the revised title and discussion, of the added analysis on Facebook’s ad delivery system, and of the clarification of limitations, such as the lack of a direct measure of pre-survey interest. We also appreciate the positive feedback on our improved modeling presentation and responses to generalizability concerns.

**Remaining Concerns and Suggestions:**

**Generalizability Beyond Facebook:**

While the authors now frame the study more narrowly, some claims in the abstract and discussion could still suggest broader relevance to “online surveys” generally. I recommend softening these statements further to reinforce that the results may not translate to other platforms with different audience structures or ad delivery systems.

We thank the reviewer for this comment. In response, we have carefully revised the abstract and discussion sections to more clearly reflect the platform-specific nature of our findings. Specifically, we have emphasized that our results pertain to Facebook-based recruitment. The revised version now reads as follow:

- Abstract:

"Overall, our findings do not provide consistent evidence that higher topic salience in our Facebook-based recruitment materials systematically influenced survey responses. However, in specific countries, certain recruitment images were linked to variations in COVID-19 threat perception and uptake of preventive behaviors. These context-specific effects highlight the importance of careful recruitment design for Facebook-based surveys during health crises."

- Lines 587-590:

“Our findings provide limited support for our initial hypothesis that, in the context of Facebook-based recruitment, higher topic salience in the survey recruitment materials would lead to systematically different survey responses.”

- Lines 602-605:

“In conclusion, while topical self-selection had a limited overall impact, context-specific image effects suggest that careful recruitment design remains important for Facebook recruited surveys during health crises.”

**Unmeasured Motivation and Interest:**

The authors acknowledge they did not measure respondents’ interest in COVID-19 or their reaction to specific ad images. This limits interpretability of the observed differences in survey participation across image types. Future studies could address this by including motivation-related questions or pre-randomizing image exposure more rigorously.

We thank the reviewer for highlighting this important point. We agree that the absence of direct measures of respondent motivation and interest limits the interpretability of differences in participation across image types. In response, we have expanded the suggestions of how future research could address this point. In the revised limitation section it now reads:

Lines 543-545:

“Future studies could address this issue by including a question about respondents' interest in the survey topic or motivation to participate. “

Lines 549-550:

“In addition, the A/B testing feature in the campaign setup could be used to randomize the display of images.”

**Opaque Ad Delivery Mechanism:**

Despite additional analyses, Facebook’s ad optimization algorithm remains a non-transparent influence on who sees what ad. While the authors have adjusted for some of this bias through demographic controls and modeling, the underlying ad delivery mechanism still introduces uncertainty into causal interpretation. Running separate ad campaigns per image, as the authors suggest, would be a stronger solution.

We thank the reviewer for this thoughtful observation. We agree that Facebook's opaque ad delivery algorithm remains a significant source of uncertainty. In response, we have now highlighted this limitation more clearly in our limitations section before suggesting separate ad campaigns as a potential strategy for future studies.

Lines 569-570:

“However, it is important to acknowledge that the ad optimization algorithm remains opaque to researchers outside of Meta.”

**Crisis-Specific Context:**

Given the timing of the survey during the COVID-19 pandemic, user engagement with health content was likely heightened. This context should be more clearly noted as limiting generalizability to other public health topics or less urgent health issues.

We thank the reviewer for raising this point. We agree that the heightened public attention to health during the COVID-19 pandemic likely influenced user engagement with our survey content, as we also state at the beginning of our limitations section where we describe the timing of our data collection. In response to the reviewer's comment, we have added a sentence to this section that explicitly states the limited generalizability to other public health topics or less pressing health issues. On lines 527-529 of the revised manuscript, it now reads:

“This may limit the generalizability of our findings to other public health topics or to contexts with less urgent health threats.”

We hope that this addition more clearly communicates the implications of this limitation for interpreting and generalizing our findings.

**Conclusion:**

Overall, this revised manuscript represents a substantial improvement and makes a valuable contribution to the literature on digital recruitment and selection bias. While some methodological limitations remain, they are now better acknowledged and reasonably addressed. I am satisfied with the revisions and would support publication after minor final refinements.

We thank the reviewer for their thoughtful and constructive feedback throughout the review process. We are grateful for the recognition of the improvements made and are pleased that the manuscript is now seen as a valuable contribution to the literature on digital recruitment and selection bias.
